# Generation of Human Induced Pluripotent Stem Cell‐Derived Bona Fide Neural Stem Cells for Ex Vivo Gene Therapy of Metachromatic Leukodystrophy

**DOI:** 10.5966/sctm.2015-0414

**Published:** 2016-09-16

**Authors:** Vasco Meneghini, Giacomo Frati, Davide Sala, Silvia De Cicco, Marco Luciani, Chiara Cavazzin, Marianna Paulis, Wieslawa Mentzen, Francesco Morena, Serena Giannelli, Francesca Sanvito, Anna Villa, Alessandro Bulfone, Vania Broccoli, Sabata Martino, Angela Gritti

**Affiliations:** ^1^ San Raffaele Telethon Institute for Gene Therapy, Division of Regenerative Medicine, Stem Cells and Gene Therapy, Istituto di Ricovero e Cura a Carattere Scientifico San Raffaele, Milan, Italy; ^2^ National Research Council, Milan, Italy; ^3^ Humanitas Clinical and Research Center, Rozzano, Italy; ^4^ BioFlag Ltd., Pula, Cagliari, Italy; ^5^ Biochemistry and Molecular Biology Unit, Department of Chemistry, Biology and Biotechnologies, University of Perugia, Perugia, Italy; ^6^ Division of Neuroscience, Istituto di Ricovero e Cura a Carattere Scientifico San Raffaele, Milan, Italy; ^7^ Anatomy and Histopathology Department, Istituto di Ricovero e Cura a Carattere Scientifico San Raffaele, Milan, Italy

**Keywords:** Pluripotent stem cells, Oligodendrocytes, Intracerebral transplantation, Neural stem cell, Metachromatic leukodystrophy, Gene therapy

## Abstract

Allogeneic fetal‐derived human neural stem cells (hfNSCs) that are under clinical evaluation for several neurodegenerative diseases display a favorable safety profile, but require immunosuppression upon transplantation in patients. Neural progenitors derived from patient‐specific induced pluripotent stem cells (iPSCs) may be relevant for autologous ex vivo gene‐therapy applications to treat genetic diseases with unmet medical need. In this scenario, obtaining iPSC‐derived neural stem cells (NSCs) showing a reliable “NSC signature” is mandatory. Here, we generated human iPSC (hiPSC) clones via reprogramming of skin fibroblasts derived from normal donors and patients affected by metachromatic leukodystrophy (MLD), a fatal neurodegenerative lysosomal storage disease caused by genetic defects of the arylsulfatase A (ARSA) enzyme. We differentiated hiPSCs into NSCs (hiPS‐NSCs) sharing molecular, phenotypic, and functional identity with hfNSCs, which we used as a “gold standard” in a side‐by‐side comparison when validating the phenotype of hiPS‐NSCs and predicting their performance after intracerebral transplantation. Using lentiviral vectors, we efficiently transduced MLD hiPSCs, achieving supraphysiological ARSA activity that further increased upon neural differentiation. Intracerebral transplantation of hiPS‐NSCs into neonatal and adult immunodeficient MLD mice stably restored ARSA activity in the whole central nervous system. Importantly, we observed a significant decrease of sulfatide storage when ARSA‐overexpressing cells were used, with a clear advantage in those mice receiving neonatal as compared with adult intervention. Thus, we generated a renewable source of ARSA‐overexpressing iPSC‐derived bona fide hNSCs with improved features compared with clinically approved hfNSCs. Patient‐specific ARSA‐overexpressing hiPS‐NSCs may be used in autologous ex vivo gene therapy protocols to provide long‐lasting enzymatic supply in MLD‐affected brains. Stem Cells Translational Medicine *2017;6:352–368*

**Significance Statement**

The development of effective and safe neural stem cell‐based therapies will depend on procedures that yield well‐characterized and expandable autologous cell lines suitable for transplantation that could meet stringent safety criteria and bypass ethical concerns. This study showed that bona fide neural stem cells generated via somatic cell reprogramming from fibroblasts of patients affected by a genetic demyelinating lysosomal storage disease can be safely and efficiently engineered and may serve as a stable source of therapeutic enzyme to ameliorate pathology upon intracerebral transplantation and oligodendroglial differentiation in a relevant animal model of the disease.

## Introduction

Neural stem/progenitor cells (NSCs) are self‐renewing, multipotent populations that can be isolated from different regions of the mammalian central nervous system (CNS), expanded in vitro, genetically manipulated, differentiated, and reintroduced into a developing, adult, or pathologically altered CNS 1, 2. Despite their limited potential to replace damaged or lost cells, their ability to secrete therapeutic molecules coupled to potent immunomodulatory and neuroprotective properties 3, 4, 5 places these cells as promising therapeutics for CNS disorders. Based on solid preclinical data proving the safety and efficacy of NSCs isolated from the human fetal CNS (hfNSCs) 6, 7 upon transplantation in animal models of several neurodegenerative diseases 8, 9, 10, 11, 12, clinical‐grade hfNSC therapeutic products are currently being assessed in phase I/II clinical trials for different neurodegenerative disorders. Although preliminary results suggest safety of the procedure and of the cell product, the efficacy has yet to be determined 13, 14. The allogeneic nature of these cells, potential ethical concerns, and issues related to clinical‐grade, large‐scale production of hfNSCs still represent major caveats that limit the full clinical exploitation of hfNSC‐based therapies.

In the search for alternative cell sources, human induced pluripotent stem cells (hiPSCs) represent a superior candidate when considering their extensive self‐renewal ability and wide developmental potential 15, 16, 17. The availability of patient‐specific hiPSC‐derived NSCs (hiPS‐NSCs) 18, 19, 20, 21, 22, 23 may overcome immunological issues as well as ethical concerns. In addition, combining iPSC technology with novel gene transfer/gene editing strategies has great potential for developing autologous cell‐based treatments for genetic diseases, including rare metabolic diseases with severe CNS involvement 24, 25, 26. A number of issues need to be addressed before high‐quality patient‐specific hiPS‐NSCs can be derived for future clinical applications. The steps toward a safe human hiPS‐NSC‐based cell therapy would include the development of footprint‐free iPSCs (no genome‐sequence modifications), robust gene transfer/editing protocols, and, most importantly, differentiation and selection of iPSC‐derived bona fide NSC populations that share with allogeneic hfNSCs phenotype and functional identity, including low propensity toward reactivation of proliferation program. Finally, safety and efficacy of these hiPS‐NSC populations need to be tested in relevant diseases models.

Metachromatic leukodystrophy (MLD) is a lysosomal storage disorder (LSD) caused by mutations in the gene coding for the lysosomal enzyme arylsulfatase A (ARSA) (EC 3.1.6.8), a key enzyme in the catabolism of myelin‐enriched sphingolipids 27, 28. The progressive accumulation of sulfatides in the lysosomes of neurons and myelinating cells results in severe demyelination and neurodegeneration of the CNS and peripheral nervous system (PNS) 29, progressive neurologic deficits, and premature death in the infantile forms. Enzymatic reconstitution mediated by hematopoietic stem cell (HSC) gene therapy (GT) provides supraphysiological enzymatic activity in cerebrospinal fluid (CSF) and leads to prevention of CNS pathology in the treated asymptomatic patients 30. However, this mechanism takes a few months to establish and might not benefit the fast‐developing disease in the majority of already‐symptomatic patients 31, 32. Several preclinical studies suggest that NSC transplantation might overcome this limit, providing CNS tissues with rapid and widespread enzymatic supply as well as immunomodulatory/neuroprotective factors that might counteract the progressive neuroinflammation and tissue damage 4, 9, 10, 33, 34, 35, 36.

Here, we have initially established protocols to efficiently differentiate normal and MLD hiPSCs into hiPS‐NSCs that are phenotypically and functionally comparable to hfNSCs and share indistinguishable global gene expression profiling. Subsequently, we have optimized gene‐transfer protocols to overexpress a functional human *ARSA* gene (*hARSA*) in hiPSCs derived from MLD patients. Upon intracerebral transplantation in immunodeficient MLD mice, ARSA‐overexpressing MLD hiPS‐NSCs displayed stable engraftment and a favorable safety profile, providing an early (in the case of neonatal intervention), robust, and long‐lasting ARSA supply that resulted in significant prevention/reduction of sulfatide storage. These results show that bona fide NSCs generated via reprogramming from MLD patients can be safely and efficiently engineered and may serve as autologous cell‐based vehicle for long‐lasting ARSA supply to provide therapeutic benefit in MLD‐affected brains.

## Materials and Methods

### Vectors

#### Reprogramming Vector

The reprogramming lentiviral vector (LV.OSK) carries a monocistronic Cre‐excisable expression cassette encoding for the recoded sequences of human *OCT4*, *SOX2*, and *KLF4* genes separated by small 2A self‐cleaving peptide sequences downstream to the retroviral spleen focus‐forming virus (*SFFV*) promoter (kindly provided by A. Lombardo and L. Naldini). Titer of concentrated vector was 1.6 × 10^9^ transducing units (TU)/ml, and infectivity was 1.7 × 10^4^ TU/ng of p24.

#### ARSA‐Expressing Vectors

The bdLV.hARSA.GFP is a vesicular stomatitis virus (VSV)‐pseudotyped third‐generation bidirectional lentiviral vector (bdLV) allowing the coordinate expression of the *hARSA* coding sequence C‐terminally tagged with the influenza hemagglutinin (HA) epitope and *green fluorescent protein* (*GFP*) reporter gene driven by the *human phosphoglycerate kinase promoter*. Production and titration were previously described 37, 38. Titer of concentrated vector was 4 × 10^9^ TU/ml, and infectivity was 4 × 10^4^ TU/ng of p24. The LV.hARSA is a VSV‐pseudotyped third‐generation LV encoding for codon optimized recoded sequence of the *hARSA* gene 30. Titer of concentrated vector was 4.86 × 10^8^ TU/ml, and infectivity was 7.28 × 10^4^ TU/ng of p24.

### Cell Culture

Composition of all culture media is detailed in the supplemental online data. Human cells were used according to the guidelines on human research issued by the institution's ethics committee, in the context of the protocol TIGET‐HPCT.

#### Reprogramming of Human Fibroblasts

Skin fibroblasts derived from MLD patients and from normal donors (ND) were obtained from the Cell Line and DNA Bank of Patients affected by Genetic Diseases (Institute Gaslini, Genova, Italy, http://www.gaslini.org). Fibroblasts (10,000 cells per cm^2^) were exposed to LV.OSK (multiplicity of infection [MOI] 1–3) in human fibroblasts medium (HFM) supplemented with 8 µg/ml Polybrene (Sigma‐Aldrich, St. Louis, MO, http://www.sigmaaldrich.com) for 24 hours. Then, fresh HFM was added for additional 24 hours. On day 3, fibroblasts were enzymatically detached and plated on mitomycin C (Sigma‐Aldrich)‐inactivated mouse embryonic fibroblasts (MEFs). On day 8, cells were exposed to hiPSC medium. The medium was changed daily, until the appearance of hiPSC clones (1–3 months) that were manually picked and plated on mitomycin C‐inactivated MEFs. Stable hiPSC lines were then expanded by enzymatic dissociation with collagenase IV (1 mg/ml; Thermo Fisher Scientific Life Sciences, Waltham, MA, https://www.thermofisher.com) on mitomycin C‐inactivated MEFs. hiPSCs were characterized for pluripotency by means of immunohistochemistry (expression of pluripotency markers), alkaline phosphatase staining, and molecular (gene expression studies, promoter methylation by bisulfite sequencing), and functional analysis (embryoid body and teratoma assay).

#### hiPSC‐Derived NSCs

hiPSCs were detached with dispase (Thermo Fisher Scientific Life Sciences) and cultured as embryoid bodies (EBs) in EB medium. On day 4, EBs were plated on Matrigel (BD Biosciences, San Jose, CA, http://www.bdbiosciences.com)‐coated dishes and grown in EB medium supplemented with NOGGIN (250 ng/ml, R&D Systems, Minneapolis, MN, https://www.rndsystems.com). At day 10, medium was replaced with EB medium supplemented with Sonic Hedgehog (SHH; 20 ng/ml, R&D Systems) and fibroblast growth factor 8 (FGF8; 100 ng/ml, R&D Systems). Upon appearance of rosette‐like structures (day 14), medium was changed to BASF medium (brain‐derived neurotrophic factor [BDNF], ascorbic acid, SHH, and FGF8). At day 22, FGF8 was withdrawn, and cells were maintained in BAS medium (BDNF, ascorbic acid, and SHH). At day 29, cells were detached with Accutase (Thermo Fisher Scientific Life Sciences) and plated on poly‐l‐ornithine (20 μg/ml, Sigma‐Aldrich)/laminin (10 μg/ml, Thermo Fisher Scientific Life Sciences)‐coated dishes in hiPS‐NSC proliferation medium (NPM), and were then expanded up to 10 passages.

#### Fetal‐Derived Human NSCs

We used two independent hfNSC lines that were previously described 39. Cells were expanded in mitogen‐supplemented serum‐free medium and used between passages 18 and 25. The individual hfNSC lines behaved similarly in all the experimental conditions tested.

#### hiPS‐NSC‐Derived Neurons and Glial Cells

hiPS‐NSCs were detached with 0.5 mM EDTA (Thermo Fisher Scientific Life Sciences), mechanically dissociated, and plated on Matrigel‐coated dishes in neuronal differentiation medium (NDM) supplemented with 20 ng/ml FGF2 (PeproTech, Rocky Hill, NJ, http://www.peprotech.com) or in NPM (for glial differentiation). After 2/3 days, cells were (a) detached with Accutase, plated on Matrigel‐coated dishes (20,000 cells per cm^2^) and grown in NDM for 25 or 50 days of neuronal differentiation; and (b) exposed every second day to increased amounts (25%, 50%, and 75%) of glial differentiation medium (GDM) supplemented with 20 ng/ml FGF2. Cells were maintained in GDM supplemented with 20 ng/ml FGF2 until day 18, when FGF2 was withdrawn. At day 38, medium was changed in glial maturation medium up to day 100.

#### LV‐Mediated Gene Transfer

MLD hiPSC colonies were expanded in feeder‐free condition in hiPSC medium (hiPSCM) supplemented with 10 µM Y‐27632 (Sigma‐Aldrich). After 3 days, hiPSCs were dissociated at single cell with Accutase and plated on Matrigel‐coated 12‐well plates (20,000 cells per well). At day 4, cells were transduced with LVs (MOI 100) in hiPSCM supplemented with 10 µM Y‐27632 and 8 µg/ml polybrene. Medium was changed 24 hours later. At confluence, cells were detached with dispase and expanded on mitomycin C‐inactivated MEFs in hiPSCM.

### Mice

NOD SCID gamma mice *Rag^−/−^;γ-chain^−/−^;As2^−/−^* mice, and *Rag^−/−^;γ-chain^−/−^;As2^+/+^* littermates were maintained in the animal facility at the San Raffaele Scientific Institute. Mice were housed in microisolators under sterile conditions and supplied with autoclaved food and water. All procedures were performed according to protocols approved by the internal Institutional Animal Care and Use Committee and reported to the Italian Ministry of Health.

### Cell Transplantation

hiPS‐NSCs (ND and MLD^bdLV.hARSA.GFP^) and hfNSCs were dissociated to single cells and resuspended in culture medium + 0.1% DNase I (125,000 cells per µl). Cells (250,000 cells per 2 µl) were slowly injected in the right hemisphere of postnatal day 60 (PND60) *Rag^−/−^;γ-chain^−/−^;As2^−/−^* mice by using a 33G needle‐Hamilton syringe. Stereotactic coordinates for the injection site (corpus callosum) were anteroposterior 1.1, medial lateral 1.1, and dorsal ventral 2.0 (mm from Bregma, according to the Paxinos mouse brain atlas). For neonatal transplantation, hiPS‐NSCs prepared as described above were bilaterally injected in the lateral ventricles of PND1 mice (200,000 cells per 2 µl per injection site) as previously described 35, 36. At 3 and 6 months after transplantation, mice were deeply anesthetized and intracardially perfused with 0.9% NaCl plus 1 µl/ml heparin, followed by 4% paraformaldehyde to collect brain and tissues. Untreated *Rag^−/−^;γ-chain^−/−^;As2^−/−^* and *Rag^−/−^;γ-chain^−/−^;As2^+/+^* age‐matched littermates were used as controls.

### Immunocytochemistry

Immunocytochemistry on fixed cells and tissues was performed as previously described 39, 40. Details on the protocol and a list of antibodies used can be found in the supplemental online data.

### Cell Counts

#### Cell Characterization In Vitro

The extent of neuronal and glial differentiation was assessed using antibodies against lineage‐ and stage‐specific markers. Five to 10 blind selected fields per sample (>200 cells per field) were counted for each time point.

#### Cell Engraftment, Differentiation, and Migration In Vivo

Human cells were identified by using anti‐human antibodies. The number of engrafted cells was assessed in 40‐µm‐thick coronal brain sections (15–18 sections per mouse, corresponding to one out of six series) by using anti‐hNuclei. The number of hNuclei‐positive cells per section was multiplied by 6 to obtain an estimate of the total number of engrafted cells per brain. The percentage of engraftment was expressed as: (total number of engrafted cells/total number of transplanted cells) × 100. The cell type composition of engrafted cells was assessed by using immunofluorescence followed by confocal microscopy analysis in coronal brain sections (three fields per section; three slices per mouse) using anti‐hNuclei or anti‐mitochondria antibodies in combination with antibodies against lineage‐specific markers and nuclear counterstaining.

### Statistical Analysis

Data were analyzed with GraphPad Prism for Macintosh (version 5.0a; GraphPad Software Inc., La Jolla, CA, http://www.graphpad.com) and expressed as mean ± SEM, unless otherwise stated. Student's *t* test and one‐ or two‐way analysis of variance (ANOVA) followed by post test were used for statistical analyses according to datasets (statistical significance: *p* < .05). The number of samples and the statistical test used are indicated in the figure legends.

Details regarding karyotype analysis, embryoid body and teratoma assays, evaluation of vector copy number, bisulfite sequencing, microarrays, Cre‐recombinase excision, Western blot, enzymatic activity, flow cytometry, and Alcian blue staining can be found in the supplemental online data.

## Results

### Generation and Quality Assessment of Normal Donor hiPSC Lines

**Figure 1:**
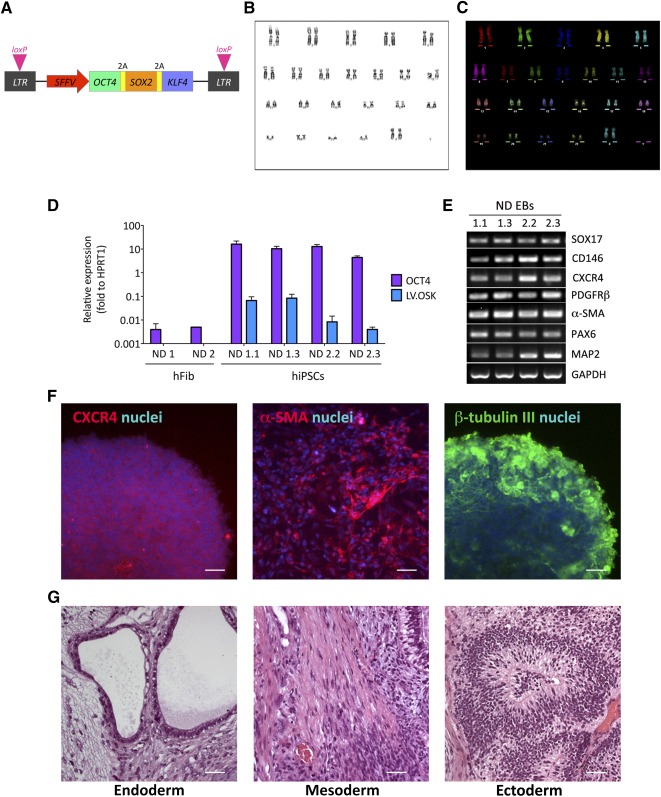
Characterization of hiPSC lines obtained upon reprogramming of skin‐derived fibroblasts from normal donors. **(A):** The reprogramming vector (LV.OSK) is a self‐inactivating lentiviral vector (LV) containing *LoxP* sites in the *LTR* and expressing the human *OCT4*, *KLF4*, and *SOX2* genes from a monocistron recoded for robust expression. **(B, C):** Normal karyotype (46, XX) in hiPSC clone ND1.3 assessed by traditional G‐band karyotype analyses **(B)** and multicolor fluorescence in situ hybridization analyses **(C)**. Analyses were performed on at least 15 metaphases per clone in all hiPSC lines reported in supplemental online Table 1. **(D):** Bar graph showing mRNA expression of endogenous OCT4 and of the reprogramming cassette (LV.OSK) (assessed by quantitative reverse‐transcriptase polymerase chain reaction [qRT‐PCR]) in ND1 and ND2 fibroblast cell lines (hFib) and in the relative hiPSC clones. Data are normalized on *HPRT1* expression (housekeeping gene) and are expressed as the mean ± SEM (*n* = 3 experiments in triplicate). **(E):** mRNA expression of endodermal (SOX17, CD146, and CXCR4), mesodermal (PDGFRβ and α‐SMA), and ectodermal (PAX6 and MAP2) markers (assessed by RT‐PCR) in embryoid bodies derived from hiPSC clones. *GAPDH* is used as a housekeeping gene. **(F):** Representative immunofluorescence images showing the presence of cells expressing endodermal (CXCR4), mesodermal (α‐SMA), and ectodermal (β‐tubulin III) markers within hiPSC‐derived EBs. Nuclei were counterstained with 4′,6‐diamidino‐2‐phenylindole (blue). Scale bars = 100 µm. **(G):** hiPSC‐derived teratomas characterized by the presence of tissues derived from the three germinal layers: endoderm (glandular epithelium), mesoderm (smooth muscle), and ectoderm (neural tube‐like structures). Scale bars = 50 µm. Abbreviations: 2A, self‐processing sequence; EBs, embryoid bodies; *GAPDH*, glyceraldehyde‐3‐phosphate dehydrogenase; hFib, fibroblast cell line; hiPSCs, human induced pluripotent stem cells; *LTR*, long terminal repeat; ND, normal donor; *SFFV*, spleen focus‐forming virus.

We transduced normal donor‐derived adult (ND1) and neonatal (ND2) skin fibroblasts at MOI = 1 or 3 using a monocistronic Cre‐excisable lentiviral vector (LV) in which the expression of the reprogramming factors *OCT4*, *SOX2*, and *KLF4* was driven by the *SFFV* retroviral promoter, known to be silenced in pluripotent cells (referred to as LV.OSK) (Fig. 1A). We obtained 10–12 hiPSC clones with vector copy number (VCN) ranging from 1 to 4. We selected two clones for each donor cell line (supplemental online Table 1) based on genomic stability assessed by G‐band karyotype analyses (Fig. 1B) and fluorescence in situ hybridization analyses (Fig. 1C). These clones were used for all the subsequent analysis.

Residual expression of reprogramming factors may predispose iPSCs to genomic instability and alter their differentiation potential 41, 42, 43. Taqman assays revealed that LV.OSK expression levels in stable hiPSC clones were 10^2^‐ to 10^3^‐fold lower when compared with endogenous OCT4 mRNA levels (Fig. 1D). The activation of the endogenous pluripotency gene network was confirmed by bisulfite genomic sequencing of CpG islands in *OCT4* and *NANOG* promoters, which indicated the transition from an inactive methylated state in fibroblasts to an active demethylated profile in hiPSC lines (supplemental online Fig. 1A). ND hiPSC lines expressed markers of stem cell pluripotency, as assessed by reverse‐transcriptase polymerase chain reaction (RT‐PCR) (NANOG, SOX2, and KLF4) and immunofluorescence analysis (OCT4, NANOG, and TRA1‐60), and displayed alkaline phosphatase (AP) activity (supplemental online Fig. 1B–1F). The negligible contribution of exogenous reprograming factors in maintaining the pluripotency program was finally confirmed by stable expression levels of OCT4 and KLF4 assessed in selected hiPSC clones, in which the reprogramming cassette was efficiently excised through Cre‐mediated recombinase strategies (supplemental online Fig. 1G, 1H).

ND hiPSC lines differentiated into embryoid bodies containing cells expressing endodermal, mesodermal, and ectodermal markers (Fig. 1E, 1F). Finally, ND hiPSCs gave rise to teratomas upon subcutaneous transplantation in immunodeficient mice (Fig. 1G). Together, these data confirm that the selected ND hiPSC clones derived from neonatal and adult skin fibroblasts display molecular, morphological, and functional features of bona fide pluripotent stem cells.

### hiPSC‐Derived Neural Stem Cells Are Molecularly, Morphologically, and Functionally Similar to Human Fetal Neural Stem Cells

**Figure 2:**
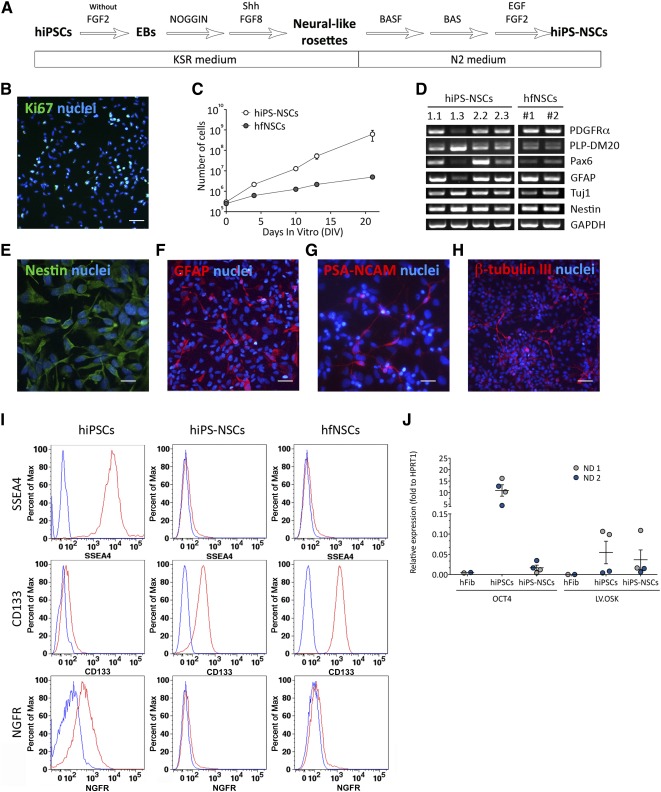
Differentiation of hiPSCs into bona fide neural stem cells. **(A):** Schematic representation of the differentiation protocol. **(B, C):** hiPS‐NSCs express Ki67 **(B)** (green) and are characterized by extensive proliferating ability, as assessed by the steady increase in the number of cells upon several subculturing passages **(C)**; allogeneic hfNSCs are shown for comparison (data are expressed as the mean ± SEM; *n* = 4 hiPS‐NSC clones and *n* = 2 hfNSC lines in duplicate). **(D):** mRNA expression of neural stem cell (NSC) markers (assessed by reverse‐transcriptase polymerase chain reaction [RT‐PCR]) in four hiPS‐NSC lines (derived from hiPSC clones ND1.1, ND1.3, ND2.2, and ND2.3) and two hfNSC lines (numbers 1 and 2). *Glyceraldehyde‐3‐phosphate dehydrogenase* is used as housekeeping gene. **(E–H):** Representative immunofluorescence pictures showing hiPS‐NSC‐derived immature neuroepithelial cells (NESTIN) **(E)**, astrocytes (GFAP) **(F)**, and neurons (PSA‐NCAM **[G]**, β‐tubulin III **[H]**); nuclei were counterstained with 4′,6‐diamidino‐2‐phenylindole (blue). Scale bars = 50 µm **(B, H)**, 10 µm **(E, G)**, and 20 µm **(F)**. **(I):** Flow cytometry histograms (percentage of the maximum) showing expression of SSEA4 (pluripotency marker), CD133 (NSC marker), and nerve growth factor receptor (neural crest cell marker) in hiPSCs, their differentiated hiPS‐NSC counterparts, and hfNSCs. Blue lines, unstained cells; red lines, stained cells. One representative histogram for each antigen is shown out of three independent experiments performed on each cell line. **(J):** Dot plot graph showing downregulation of OCT4 mRNA expression (assessed by quantitative RT‐PCR) to levels of parental fibroblasts (hFib) during the differentiation of normal donor 1 (ND1) (gray) and ND2 (blue) hiPSC clones into hiPS‐NSCs. Comparable residual expression of the reprogramming vector (LV.OSK) is detected in hiPSCs and hiPS‐NSCs. Data are normalized on *HPRT1* expression (housekeeping gene). *n* = 2 hiPS‐NSC lines and parental hiPSC clones per donor. Each dot represents the mean of three independent experiments for each clone (hiPSCs and hiPS‐NSCs) or the mean of two replicates (hFib). Abbreviations: BAS, mix of brain‐derived neurotrophic factor, ascorbic acid, and Sonic hedgehog; BASF, mix of brain‐derived neurotrophic factor, ascorbic acid, Sonic hedgehog, and fibroblast growth factor 8; d, day; DIV, days in vitro; EBs, embryoid bodies; EGF, epidermal growth factor; FGF, fibroblast growth factor; GFAP, glial fibrillary acidic protein; hfNSCs, fetal‐derived human neural stem cells; hiPSCs, human induced pluripotent stem cells; hiPS‐NSCs, human induced pluripotent stem cell‐derived neural stem cells; KSR, knockout serum replacement; Max., maximum; Pax6, paired box 6; PDGFRα, platelet‐derived growth factor receptor α; PLP‐DM20, myelin proteolipid protein DM20; Shh, Sonic hedgehog; Tuj1, β‐tubulin III.

We next sought to differentiate hiPSC clones in self‐renewable, multipotent populations of *bona‐fide* human neural stem/progenitor cells displaying functional features similar to human allogeneic fetal neural stem cells 12, 39, 44, 45. To this end, we optimized a protocol based on the sequential exposure to morphogens and/or growth factors 18 (Fig. 2A). ND hiPSC lines were differentiated into EBs in the presence of the SMAD inhibitor NOGGIN (days 3–9) and SHH+FGF8 (days 9–14) until the appearance of neural‐like rosettes (supplemental online Fig. 2). Switching to N2‐based medium supplemented with SHH, FGF8, BDNF, and ascorbic acid (days 14–28) promoted the generation and expansion of NSC‐like cells. At day 28, cells were enzymatically detached, plated on polyornithin/polylaminine‐coated dishes, and maintained in N2‐based medium supplemented with FGF2 and epidermal growth factor (neural proliferation medium [NPM]). The hiPS‐NSC populations generated by using this protocol proliferated (26.6% ± 11.1% of Ki67^+^ cells; *n* = 3) (Fig. 2B) and self‐renewed, as assessed by the steady expansion rate maintained for at least 10 subculturing passages (Fig. 2C). All hiPS‐NSC lines expressed typical hfNSC markers 39 (Fig. 2D) and were composed of Nestin^+^ cells (>80%; Fig. 2E) with subpopulations of glial fibrillary acidic protein (GFAP; Fig. 2F) and neuronal progenitors (PSA‐NCAM and β‐tubulin III; Fig. 2G, 2H). Neural induction resulted in robust expression of CD133 (Fig. 2I), a surface marker used for hfNSC prospective isolation 7, 12. Importantly, hiPS‐NSCs showed downregulation of pluripotency genes (*SSEA4* and *OCT4*; Fig. 2I, 2J). Expression of p75/NGFR was detected in <1% of hiPS‐NSCs (Fig. 2I), suggesting negative selection of putative neural crest cells driven by differentiation protocol. Overall these data suggest that the neural induction protocol applied here efficiently differentiates hiPSCs into proliferating and self‐renewing NSC‐like populations that are phenotypically similar to hfNSCs.

**Figure 3:**
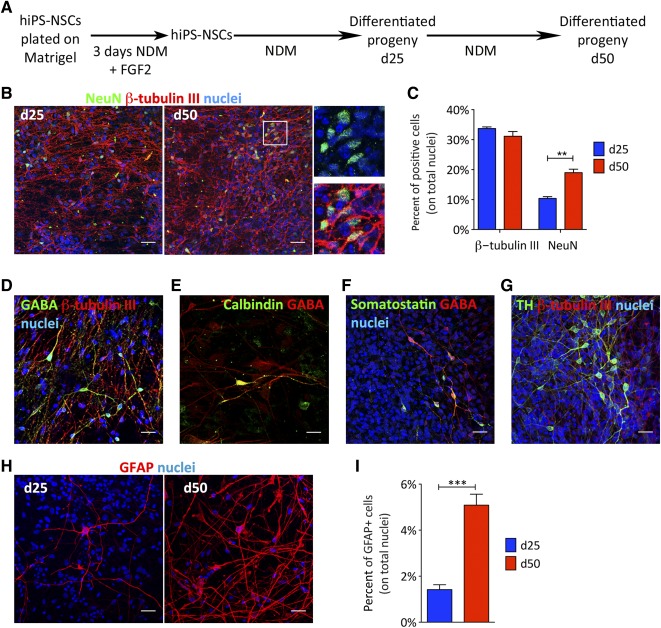
Differentiation of hiPS‐NSCs into neurons and astrocytes. **(A):** Schematic representation of the differentiation protocol. **(B):** Representative confocal images showing hiPS‐NSC‐derived neurons (NeuN, green; β‐tubulin III, red) at days 25 and 50. **(C):** Bar graph showing percentages of cells expressing neuronal markers (NeuN and β‐tubulin III) in hiPS‐NSC‐differentiated cultures at d25 and d50. **(D–G):** Representative confocal pictures showing hiPS‐NSC‐derived neurons expressing GABAergic markers (GABA **[D]**, Calbindin **[E]**, Somatostatin **[F]**) and the dopaminergic marker tyrosine hydroxylase **(G)**. **(H, I):** Representative confocal pictures and quantification performed at d25 and d50 showing the time‐dependent increase of hiPS‐NSC‐derived astrocytes (GFAP). Data in **(C)** and **(I)** are expressed as the mean ± SEM, *n* = 2 hiPS‐NSC clones analyzed in two to four independent experiments, with two coverslips per experiment per antigen. Nuclei were counterstained with ToPro‐3 (blue). ∗∗, *p* < .01; ∗∗∗, *p* < .001 (unpaired Student *t* test). Scale bars = 20 µm **(B, F–H)** or 10 µm **(D, E)**. Abbreviations: d, day; FGF2, fibroblast growth factor 2; GFAP, glial fibrillary acidic protein; hiPS‐NSCs, human induced pluripotent stem cell‐derived neural stem cells; NDM, neuronal differentiation medium; NeuN, neuronal nuclei; TH, tyrosine hydroxylase.

We next assessed the ability of hiPS‐NSC populations to generate large numbers of neurons, astrocytes, and oligodendrocytes. Progressive removal of mitogens from the culture medium is known to drive preferentially neuronal differentiation 46. We exposed hiPS‐NSCs to growth factor‐ and serum‐free neuronal differentiating medium (NDM; Fig. 3A) and analyzed cultures after 25 and 50 days in vitro (DIV). We found similar percentages of β‐tubulin III^+^ cells (∼30%–35%) at both time points, whereas percentages of neuronal nuclei‐positive (NeuN^+^) cells increased over time (10.4% ± 1.1% and 19.0% ± 2.0% at 25 and 50 DIV, respectively; Fig. 3B, 3C), suggesting progressive neuronal maturation. hiPS‐NSC‐derived cultures contained GABA‐expressing neurons (4.9% ± 3.4% and 9.0% ± 2.0% on total number of cells at 25 and 50 DIV, respectively; Fig. 3D), as well as calbindin^+^ and somatostatin^+^ cells (Fig. 3E, 3F; supplemental online Fig. 3A). In addition, we observed a time‐dependent increase of tyrosine hydroxylase (TH)‐expressing neurons (3.3% ± 2.2% and 6.9% ± 4.8% on total number of cells at 25 and 50 DIV, respectively; Fig. 3G). Expression of GABAergic and dopaminergic markers was confirmed by quantitative RT‐PCR (qRT‐PCR) analyses, which also suggested the presence of glutamatergic and, to minor extent, cholinergic and serotoninergic neurons (supplemental online Fig. 3B). The serum‐free culture conditions hampered hiPS‐NSC gliogenic differentiation (1.4% ± 0.3% and 5.1% ± 1.1% GFAP^+^ astrocytes at 25 and 50 DIV, respectively; Fig. 3H, 3I). In line with results of earlier studies using hfNSCs 12, 39, few, if none, oligodendrocytes were observed in hiPS‐NSC‐derived cultures under these differentiation conditions.

**Figure 4:**
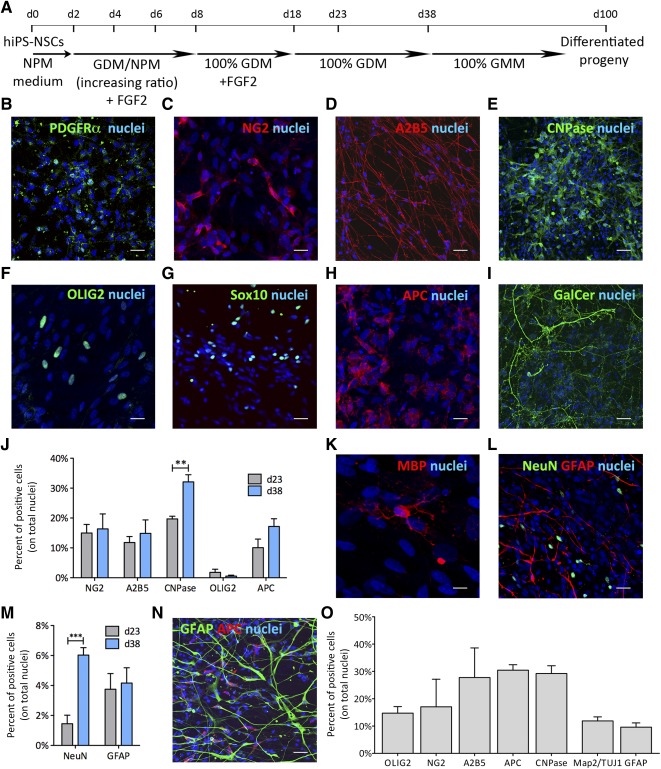
Differentiation of hiPS‐NSCs into oligodendrocytes. **(A):** Schematic representation of the differentiation protocol. **(B–I):** Representative confocal pictures showing the presence in GDM‐differentiated hiPS‐NSC cultures (d23) of cells expressing the oligodendroglial markers PDGFRα **(B)**, NG2 **(C)**, A2B5 **(D)**, CNPase **(E)**, OLIG2 **(F)**, SOX10 **(G)**, APC **(H)**, and GalCer **(I)**. **(J):** Time‐course quantification of hiPS‐NSC‐derived cells expressing oligodendroglial markers at d23 and d38 of GDM differentiation. **(K):** Representative confocal picture showing a myelin basic protein‐expressing cell in GDM‐differentiated hiPS‐NSC cultures at d100. **(L, M):** Representative confocal picture **(L)** and time‐course quantification **(M)** (d23 and d38**)** showing minor proportions of cells expressing neuronal (NeuN) and astroglial (GFAP) markers in GDM‐differentiated hiPS‐NSC cultures. **(N):** hiPS‐NSC‐derived oligodendroglial cells (APC, red) do not coexpress astroglial (GFAP, green) markers. **(O):** Bar graph showing the percentages of neuronal (Map2/TUJ1), astroglial (GFAP), and oligodendroglial cells (A2B5, NG2, OLIG2, APC, and CNPase) in GDM‐differentiated fetal‐derived human neural stem cell cultures analyzed at d23. In all the confocal pictures, nuclei were counterstained with ToPro‐3 (blue). Scale bars = 20 µm **(B, D, E, G, I, L, N)**, 10 µm **(C, F, H)**, and 5 µm **(K)**. Data in **(J), (M)**, and **(O)** are expressed as the mean ± SEM (*n* = 2 or 3 hiPS‐NSC clones, two coverslips per clone per time point per antigen; and *n* = 1 hfNSC lines (number 1), *n* = 3 independent experiments, four to six coverslips per antigen. ∗∗, *p* < .01; ∗∗∗, *p* < .001 (unpaired Student *t* test). Abbreviations: APC, adenomatous polyposis coli; d, day; GDM, glial differentiation medium; GFAP, glial fibrillary acidic protein; GMM, glial maturation medium; hiPS‐NSCs, human induced pluripotent stem cell‐derived neural stem cells; MBP, myelin basic protein; NeuN, neuronal nuclei; NPM, neural proliferation medium; PDGFR α, platelet‐derived growth factor receptor α.

In order to develop experimental conditions favoring oligodendroglial lineage commitment and enhancement of proliferation/survival of oligodendroglial progenitors (OPCs), we exploited results from earlier studies reporting the generation of OPCs from iPSC and hfNSCs 39, 47. We progressively replaced standard NPM medium with glial differentiation medium (GDM)+FGF2 (days 0–8) and exposed hiPS‐NSC cultures to GDM+FGF2 for additional 10 days (days 9–18). FGF2 was then withdrawn to promote OPC maturation. Cultures were maintained for an additional 5 or 20 days (23 and 38 DIV) (Fig. 4A) and then analyzed for cell‐type composition. Immunofluorescence analyses followed by qualitative and quantitative assessment revealed the consistent presence of cells expressing OPC markers (10%–20%; Fig. 4B–4D, 4J), as well as markers of more mature oligodendroglial cells (5%–30%; Fig. 4E–4J). Although percentages of 2,3‐phosphodiesterase cyclic nucleotide (CNPase) and adenomatous polyposis coli‐expressing cells increased over time, expression of all other markers remained stable (Fig. 4J), and we did not detect myelin basic protein‐positive (MBP^+^) cells at both time points. These data indicate that GDM differentiation resulted in efficient generation of oligodendroglial cells at different stages of commitment/differentiation, but was not sufficient for inducing a full myelinating phenotype. Indeed, only removal of FGF2, platelet‐derived growth factor receptor α (PDGFRα), and NT3 from GDM and maintenance of cultures up to 100 DIV (Fig. 4A) promoted expression of MBP in few cells (Fig. 4K). In GDM‐differentiated hiPS‐NSC cultures, we detected NeuN^+^ neurons (∼2% and ∼6% at 23 and 38 DIV, respectively) and GFAP^+^ astrocytes (<5%, stable over time) (Fig. 4L, 4M). Absence of cells coexpressing GFAP and oligodendrocyte markers suggested robust lineage specification in GDM differentiation conditions (Fig. 4N). The cell type composition of GDM‐differentiated hiPS‐NSC cultures was similar to that observed in hfNSC cultures after 23 days of differentiation in GDM (Fig. 4O). Overall, these data suggest that the neural induction protocol applied here efficiently differentiated hiPSCs into bona fide multipotent NSCs that are phenotypically and functionally similar to hfNSCs.

### hiPS‐NSCs and hfNSCs Display Similar Transcriptional Profiles

**Figure 5:**
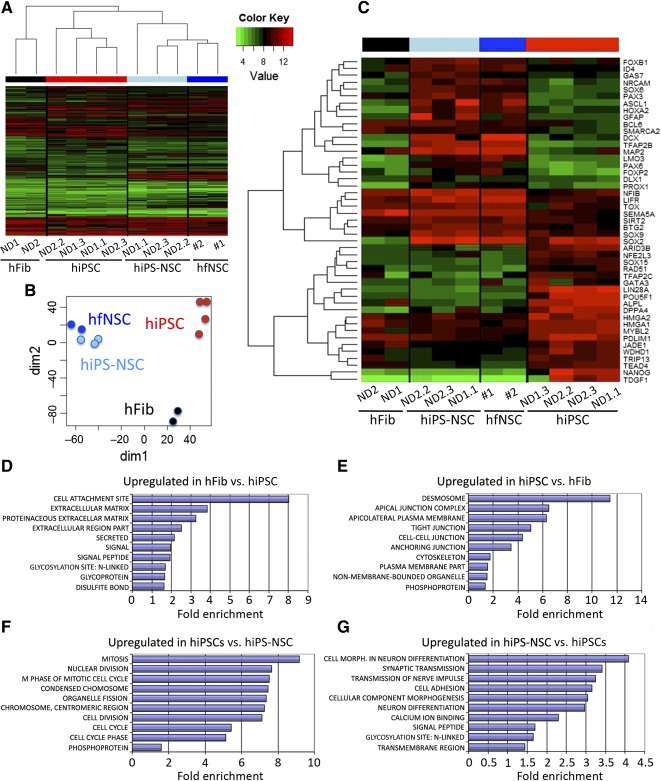
hiPS‐NSCs and hfNSCs display similar gene expression profiles. **(A):** Hierarchical clustering showing samples in columns (fibroblasts, black; hiPSCs, red; hiPS‐NSCs, light blue; and hfNSCs, blue) and genes in rows. Gene expression values are depicted according to the color scale (green, low; red, high). hiPS‐NSCs cluster separately from the parental hiPSC lines, displaying a transcriptional profile that is superimposable to that of hfNSCs. **(B):** Multidimensional scaling plot of distances between samples reveals clear separation of fibroblasts, hiPSC, and hNSC cell populations, with overlap of hiPS‐NSC and hfNSC samples. Each dot depicts an individual sample. Color code is as in **(A)** and **(C)**. **(C):** Heat map showing expression of selected genes. Genes in the upper cluster are upregulated in hiPS‐NSCs and hfNSCs, whereas genes in the lower cluster are upregulated in hiPSCs. Samples are shown in columns; rows represent color‐scaled expression values (green, low; red, high). **(D–G):** Functional enrichment within differentially expressed genes between cell populations. For each comparison, the top significantly overrepresented functional terms are shown (Benjamini *p* < .5), along with their fold enrichment (horizontal bars). Abbreviations: hFib, fibroblast cell line; hfNSC, fetal‐derived human neural stem cell; *hIPSC*, human induced pluripotent stem cell; hiPS‐NSC, human induced pluripotent stem cell‐derived neural stem cell; ND, normal donor.

In order to investigate transcriptome changes associated with the transition from hiPSCs to hiPS‐NSCs, and to assess whether hiPS‐NSCs display a bona fide NSC molecular signature, we performed expression profiling on total RNA extracted from hiPSC clones and the correspondent hiPS‐NSCs and hfNSCs (*n* = 2 independent lines) using microarray technology. Sample classification with hierarchical clustering (Fig. 5A) and multidimensional scaling (Fig. 5B) revealed highly divergent transcriptional profiles in fibroblasts (included as controls) as compared with hiPSC clones. Importantly, hiPS‐NSCs clustered separately from the parental hiPSC lines, displaying a transcriptional profile that was indistinguishable from that of hfNSCs.

We retrieved 1,185 genes differentially expressed in hiPSCs as compared with fibroblasts (supplemental online Table 2), and 817 genes differentially expressed in hiPS‐NSCs as compared with hiPSCs (supplemental online Table 3), whereas there were no genes differentially expressed in hiPS‐NSCs as compared with hfNSCs (false discovery rate < 0.1; absolute fold change > 1.5), demonstrating that these two cell populations are comparable at the transcriptional level.

Genes significantly upregulated in hiPSCs (false discovery rate < 0.05; absolute fold change > 1.5) encoded for pluripotency key factors (i.e., *POU5F1*, *NANOG*, and *LIN28A*; Fig. 5C). Genes significantly upregulated in hiPS‐NSCs (false discovery rate < 0.05 and absolute fold change > 1.5; Fig. 5C) encoded for transcription factors and genetic modifiers involved in neural tube formation and definition of dorsal‐ventral patterning (i.e., *PAX6*, *ASCL1*, and *HOXA2*), NSC proliferation/homeostasis (i.e., *TFAP2B* and *PAX3*), and neuronal/glial commitment (i.e., *GFAP*, *S100b*, *DCX*, and *MAP2*). Genes coding for pluripotency key factors or regulators of cell cycle/division (i.e., *CDC* and *KIF* families) were significantly downregulated in hiPS‐NSCs (false discovery rate < 0.01). *SOX2*, encoding a transcription factor essential for both iPSC pluripotency and hiPS‐NSCs, was similarly expressed in both cell types. Interestingly, transcription factors and genetic modifiers involved in brain cancer and tumor formation were significantly downregulated in hiPS‐NSC lines as compared with parental hiPSCs (supplemental online Table 4).

Gene Ontology (GO) functional enrichment showed that the subsets of upregulated genes in hiPSCs were significantly enriched (Benjamini test *p* < .01) in GO terms consistent with the reprogramming process (Fig. 5D, 5E), and the subset of genes upregulated in hiPS‐NSCs were enriched in GO terms consistent with neural development and function (Fig. 5F, 5G). Differential expression of several pluripotency and NSC markers was confirmed by qRT‐PCR analyses on independent samples (supplemental online Fig. 4).

### Generation of MLD hiPS‐NSCs Lines With Supraphysiological ARSA Activity

Metachromatic leukodystrophy is a fatal neurodegenerative lysosomal storage disease caused by genetic defects of the ARSA enzyme that currently lacks definitive treatment and might benefit from the development of novel gene/NSC‐based therapeutics. We took advantage of the optimized protocols described above to generate MLD hiPS‐NSCs, aiming to test the efficacy of LV‐mediated gene transfer in achieving ARSA‐(over)expressing MLD neural populations to be tested for safety and biological efficacy in relevant MLD murine models.

Patient 1 (MLD1) was compound heterozygous for c.459+1G > A/c.1216del9 and c.1049A > G/1620A > G mutations in the *ARSA* gene, which causes ARSA mRNA instability 27, 48, abolition of protein dimerization 49, and improper lysosomal localization and secretion 50, finally resulting in null enzymatic activity. Patient 2 (MLD2) fibroblasts were isolated from an aborted fetus affected by the most frequent homozygous *ARSA* mutation in Europe (c.459+1G > A), resulting in alteration of a canonical splicing site in intron 2 and absence of ARSA activity 27, 48. This mutation is usually associated with the late infantile form of the disease 48, 51. ARSA activity assessed by *p*‐nitro catechol sulfate assay 52 was undetectable in MLD1 and MLD2 fibroblasts, whereas it was 2,501.6 ± 335 nmol/h⋅mg and 749.7 ± 115.2 nmol/h⋅mg in ND1 and ND2 fibroblasts, respectively (*n* = 3 samples per cell line, at different subculturing passages).

We efficiently reprogrammed MLD1 and MLD2 fibroblasts using LV.OSK, as described for ND hiPSC clones, achieving similar VCN and a trend for increased reprogramming efficiency in fetal‐derived fibroblasts (supplemental online Table 5). Among the several clones generated, we initially selected three and four hiPSC clones from MLD1 and MLD2, respectively, that were expanded in culture. Three MLD2 hiPSC clones were then discarded because of karyotype abnormalities. The remaining MLD hiPSC lines (1.1, 1.2, 1.3, and 2.1; supplemental online Table 5) were identified as bona fide pluripotent stem cells (supplemental online Fig. 5A–5D) and subsequently used for all the analysis.

**Figure 6:**
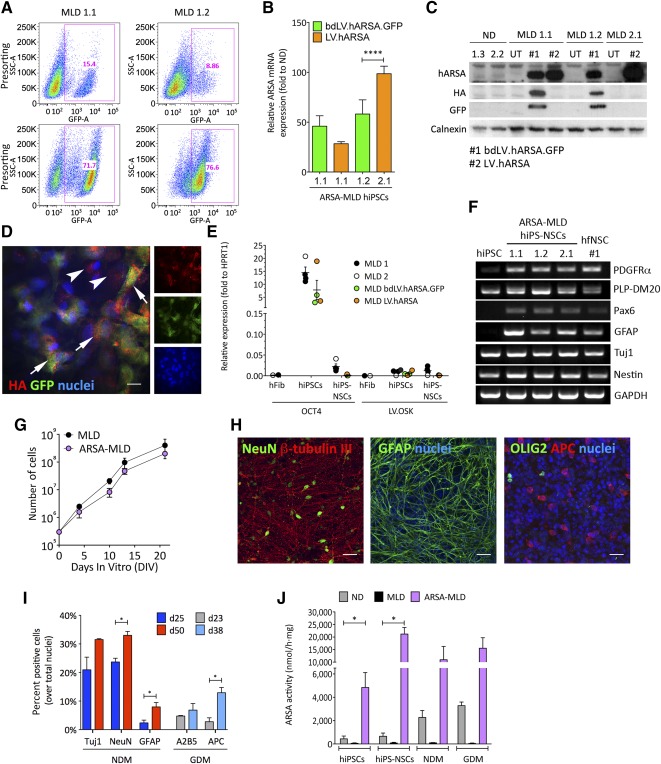
Lentiviral vector (LV)‐mediated gene transfer leads to supraphysiological ARSA activity in MLD hiPSCs and differentiated progeny. **(A):** Fluorescence‐activated cell sorting plots showing GFP^+^ (ARSA‐expressing) cells in MLD1.1 and MLD1.2 hiPSC clones transduced with bdLV.hARSA.GFP assessed pre‐ (bulk population; 9%–15% GFP^+^ cells) and postsorting (>70% GFP^+^ cells). **(B):** ARSA mRNA overexpression (assessed by quantitative reverse‐transcriptase polymerase chain reaction [qRT‐PCR]) in three ARSA‐MLD clones. Data (mean ± SEM) are expressed as fold to normal donors (*n* = 2) after normalization on *HPRT1*; *n* = 2–5 replicates per clone. ∗∗∗∗, *p* < .0001, unpaired Student *t* test. **(C):** Western blot showing ARSA overexpression in MLD hiPSC clones transduced with LV.hARSA or bdLV.hARSA.GFP (also expressing HA and GFP) as compared with undetectable expression in untransduced counterparts and physiological expression in ND hiPSC clones. Calnexin is used as loading control; 20 µg of total proteins per lane. **(D):** Representative picture (merged and individual channels) showing HA^+^GFP^+^ transduced cells (arrows) and HA^+^GFP^−^ (putative cross‐corrected cells; arrowheads) in bdLV.hARSA.GFP‐transduced hiPSCs. Nuclei are counterstained with 4′,6‐diamidino‐2‐phenylindole (blue). Scale bar = 10 µm. **(E):** Downregulation of OCT4 mRNA expression and of the reprogramming cassette (LV.OSK) (assessed by qRT‐PCR) to levels of parental fibroblasts (hFib) in MLD and ARSA‐MLD hiPS‐NSCs (bdLV.hARSA.GFP or LV.hARSA) as compared with parental hiPSCs. Data are normalized on the housekeeping gene *HPRT1*. *n* = 3 and 1 hiPS‐NSC line per second and parental hiPSC clone per second for MLD 1 and MLD 2, respectively. Each dot represents the mean of three independent experiments for each clone (hiPSCs and hiPS‐NSCs) or the mean of two replicates (hFib). **(F):** mRNA expression of neural stem cell (NSC) markers (assessed by RT‐PCR) in three ARSA‐MLD hiPS‐NSC lines and one hfNSC line (number 1). *GAPDH* is used as a housekeeping gene. **(G):** MLD and ARSA‐MLD hiPS‐NSCs display similar proliferation rate upon serial subculturing passages. **(H, I):** Representative confocal pictures **(H)** and quantification **(I)** of neurons (NeuN and β‐tubulin III), astrocytes (GFAP), and oligodendrocytes (OLIG2 and APC) derived from MLD and ARSA‐MLD hiPS‐NSCs differentiated for several days in NDM (blue and red bars) or GDM conditions (light blue and gray bars). Nuclei are counterstained with ToProIII (blue). Scale bars = 20 µm **(H)**. Data are expressed as the mean ± SEM; *n* = 2 clones (MLD1.1, MLD2.1) and 2 or 3 coverslips per time point. ∗, *p* < .05, unpaired Student *t* test. **(J):** Supraphysiological ARSA activity (measured using *p*‐nitrocatechol assay) in ARSA‐MLD hiPS‐NSCs and their differentiated progeny enriched in neurons (NDM) and oligodendrocytes (GDM) as compared with physiological levels assessed in ND hiPS‐NSCs. Untransduced MLD lines (black bars) showed undetectable ARSA activity. Data are expressed as the mean ± SEM; *n* = 2 or 3 clones per line in 2 or 3 independent experiments. ND clones: ND1.1, ND1.3; MLD clones: MLD1.1, MLD1.2, MLD2.1; MLD‐ARSA clones: MLD 1.1^bdLV.hARSA.GFP^, MLD 1.2^bdLV.hARSA.GFP^, MLD 2.1^LV.hARSA^. Abbreviations: APC, adenomatous polyposis coli; ARSA, arylsulfatase A; DIV, days in vitro; GAPDH, glyceraldehyde‐3‐phosphate dehydrogenase; GDM, glial differentiation medium; GFAP, glial fibrillary acidic protein; GFP, green fluorescent protein; HA, hemagglutinin; hARSA, human arylsulfatase A; hFib, fibroblast cell line; hfNSC, fetal‐derived human neural stem cell; hiPSC, human induced pluripotent stem cell; hiPS‐NSCs, human induced pluripotent stem cell‐derived neural stem cells; MLD, metachromatic leukodystrophy; ND, normal donor; NDM, neuronal differentiation medium; NeuN, neuronal nuclei; PLP‐DM20, myelin proteolipid protein DM20; UT, untransduced.

In order to transfer a functional human *ARSA* gene (*hARSA*) in MLD hiPSCs, we used two LV constructs: (a) a laboratory grade/scale bidirectional LV 38 coding for the *hARSA* gene tagged with HA peptide and the *GFP* gene (bdLV.hARSA.GFP); and (b) a LV batch carrying a codon optimized *hARSA* coding sequence and manufactured according to the same process used for the current ex vivo HSC gene therapy trial of MLD 30, although the procedure was not performed under strict good manufacturing practices (LV.hARSA). We achieved efficient transduction of MLD1.1, MLD1.2, and MLD2.1 hiPSC clones using both LV constructs (supplemental online Table 6). The percentage of GFP^+^ cells in bdLV.hARSA.GFP‐transduced cells (MLD^bdLV.hARSA.GFP^) ranged between 9% and 15%, reaching ∼70% upon fluorescence‐activated cell sorting (FACS) (Fig. 6A). The homogeneous populations of GFP^+^ cells overexpressed hARSA mRNA (40‐ to 100‐fold the physiological levels; Fig. 6B) and protein (Fig. 6C). ARSA overexpression correlated to enzymatic activity, which reached fourfold the physiological levels (supplemental online Table 6). Notably, immunofluorescence analyses performed on presorted MLD^bdLV.hARSA.GFP^ cells revealed the presence of GFP^−^HA^+^ putative cross‐corrected cells (Fig. 6D). The absence of GFP in LV.hARSA‐transduced hiPSCs (MLD^LV.hARSA^) hampered the possibility to enrich for transduced cells. However, ARSA activity in bulk MLD^LV.hARSA^ hiPSCs was 8‐ to 17‐fold higher as compared with ND hiPSCs (supplemental online Table 6), suggesting enhanced protein expression and/or activity due to codon optimization. LV transduction did not alter the pluripotency program, as suggested by similar expression of pluripotency genes in untransduced (UT) and ARSA‐overexpressing MLD (MLD^bdLV.hARSA.GFP^ and MLD^LV.hARSA^) hiPSCs, collectively called ARSA‐MLD hiPSCs· (supplemental online Fig. 5E).

We then generated UT and ARSA‐MLD hiPS‐NSCs using the protocol previously applied to ND hiPSCs (Fig. 2A). All MLD hiPS‐NSC lines displayed downregulation of pluripotency genes, upregulation of NSC markers (Fig. 6E, 6F; supplemental online Fig. 5E), and bona fide NSC functional features (Fig. 6G–6I). Importantly, ARSA‐MLD hiPS‐NSCs and their oligodendroglial‐ and neuronal‐enriched progeny displayed consistent supraphysiological ARSA activity along the transition from hiPSCs to neural precursors to differentiated progeny (Fig. 6J). Of note, ARSA overexpression was not affected in Cre‐recombinase‐treated MLD iPSCs and neural progeny, indicating high specificity of the Cre‐mediated excision of the reprogramming cassette (supplemental online Fig. 6).

### Widespread and Stable ARSA Activity Upon Intracerebral Transplantation of hiPS‐NSCs in Adult and Neonatal MLD Immunodeficient Mice

**Figure 7:**
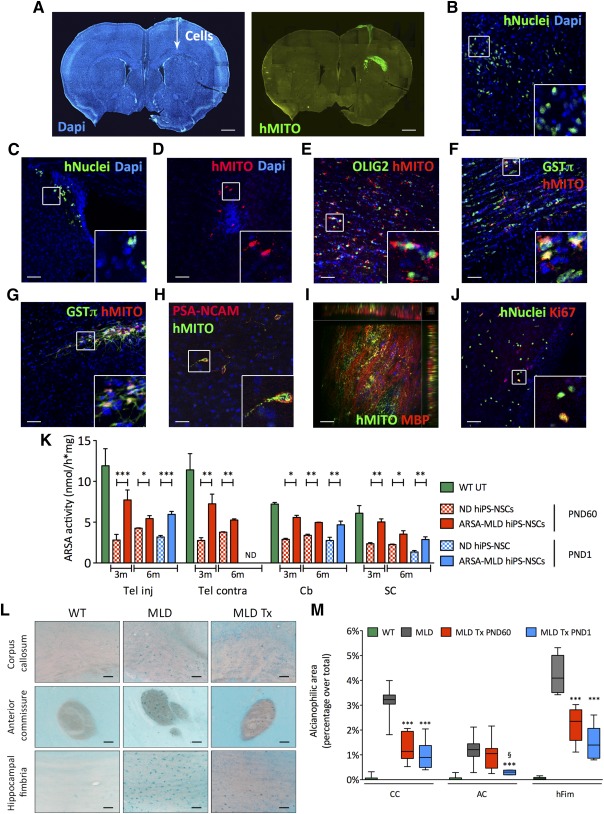
Stable engraftment of hiPS‐NSCs upon intracerebral transplantation in immunodeficient MLD mice provides widespread ARSA supply and amelioration of sulfatide storage. **(A–D):** ND or ARSA‐MLD hiPS‐NSCs (250,000 cells per mouse) were transplanted unilaterally in the corpus callosum of 2‐month‐old immunodeficient MLD mice (*n* = 12 and 9 mice transplanted with ND and ARSA‐MLD hiPS‐NSCs, respectively). Three months after transplant engrafted hiPS‐NSCs (hMITO‐ or hNuclei‐positive cells) were present close to the injection site **(A)**, in the striatum **(B)**, in the subventricular zone of the lateral ventricles **(C)**, or in the olfactory bulbs **(D)**. **(A–D):** Nuclei counterstained with DAPI. **(E–G):** Engrafted hiPS‐NSCs **(E, F)** and fetal‐derived human neural stem cells **(G)** (hMITO, red) expressed OLIG2 and GST‐π (oligodendroglial markers). **(H):** Few human‐derived cells (hMito, green) expressed PSA‐NCAM (neuronal marker, red). **(I):** Z‐stack confocal image showing human‐derived cells (hMito, green) engrafted in the corpus callosum and expressing myelin basic protein (marker of myelinating oligodendrocytes, red). Side views show the *xz* and *yz* planes of the stack. **(J):** Scattered hiPS‐NSCs (hNuclei, green) in or close to the subventricular zone expressed the proliferation marker Ki67 (red). Insets show high‐magnification of boxed areas. Scale bars = 600 µm **(A)**, 50 µm **(B–G, J)**, or 25 µm **(H, I** **)**. **(K):** ARSA activity in the telencephalon (injected and contralateral), cerebellum and spinal cord of MLD mice transplanted at PND60 or PND1 with ND or ARSA‐MLD hiPS‐NSCs and analyzed at 3 months (*n* = 4 mice per group) and 6 months after treatment (*n* = 2 and 3 mice per group for PND60 and PND1 transplantation, respectively). Data are expressed as the mean ± SEM. ∗, *p* < .05; ∗∗, *p* < .01; ∗∗∗, *p* < .001 (two way analysis of variance [ANOVA] followed by Bonferroni post test). **(L):** Sulfatide storage (Alcian blue staining) in the corpus callosum, anterior commissure, and hippocampal fimbria of 5‐month‐old untreated WT and MLD mice and of age‐matched MLD mice transplanted at PND60 with ARSA‐overexpressing hiPS‐NSCs. Scale bars = 50 µm. **(M):** Quantification of Alcian blue‐positive areas revealed reduced sulfatide storage in CC, AC, and hFim of MLD mice transplanted at PND60 (red bars) and PND1 (blue bars) with ARSA‐overexpressing hiPS‐NSCs and analyzed at 6 months of age. Data are mean ± SEM; *n* = 2 mice per group; 4 images per section and 2 sections per mouse. ∗∗∗, *p* < . 001 versus MLD; §, *p* < .05 versus MLD Tx PND60 (one‐way ANOVA followed by Bonferroni post test). Abbreviations: AC, anterior commissure; ARSA, arylsulfatase A; Cb, cerebellum; CC, corpus callosum; DAPI, 4′,6‐diamidino‐2‐phenylindole; GST‐π, glutathione *S*‐transferase‐π; hFim, hippocampal fimbria; hiPS‐NSCs, human induced pluripotent stem cell‐derived neural stem cells; hMITO, human mitochondrial protein; m, months; MLD, metachromatic leukodystrophy; ND, normal donor; PND60, postnatal day 60; SC, spinal cord; Tel, telencephalon; Tx, transplanted; UT, untreated; WT, wild type.

In order to assess the potential of hiPS‐NSCs to engraft, survive, and provide a source of functional ARSA enzyme in MLD CNS tissues, we transplanted ND and ARSA‐MLD hiPS‐NSCs into the corpus callosum of postnatal day 60 immunodeficient MLD mice (*Rag^−/−^;γ‐chain^−/−^;As2^−/−^*; 250,000 cells per animal; one unilateral injection; *n* = 9–12 animals per group). MLD mice (*n* = 4) transplanted with hfNSCs were used as controls (Fig. 7A; supplemental online Table 7). Human cells engrafted in MLD brains were detected by means of antibodies that specifically recognize human mitochondrial (hMito) and nuclear (hNuclei) proteins 39 (Fig. 7A). Analysis performed at 3 months after treatment revealed variable cell engraftment in mice transplanted with ND and MLD‐ARSA hiPS‐NSCs (12.57% ± 3.25%; range 1.65%–25.54%; *n* = 7) and in hfNSC‐transplanted controls (2.41% ± 0.53%; range 1.11%–3.78%; *n* = 4; supplemental online Fig. 7A). Similar yield of engraftment was detected in two hiPS‐NSC‐transplanted mice analyzed at 6 months after treatment (1.5% and 5%; supplemental online Fig. 7A). Engrafted cells migrated from the injection site in the ipsilateral hemisphere, distributing in white and gray matter areas, including the striatum (Fig. 7B), the subventricular zone (SVZ) of the lateral ventricles (Fig. 7C), and the olfactory bulbs (Fig. 7D). Comparable distance and pattern of cell migration were observed in MLD mice transplanted with hiPS‐NSCs (2,573.0 ± 206.9 μm; *n* = 9) and hfNSCs (2,160.0 ± 353.3 μm; *n* = 4; supplemental online Fig. 7A). Occasionally, engrafted cells were detected in the contralateral corpus callosum (supplemental online Fig. 7B). A relevant proportion of engrafted cells expressed the oligodendroglial markers OLIG2 (Fig. 7E) and glutathione *S*‐transferase‐π (GST‐π; 55.6% ± 8.8% of the total hNuclei‐positive cells; *n* = 9; Fig. 7F, 7G). Only a small percentage of human‐derived cells expressed neuronal markers (PSA‐NCAM), whereas higher numbers of engrafted cells expressed markers of astrocytes (S100β) and oligodendrocytes (GST‐π) (Fig. 7H; supplemental online Fig. 7F), indicating a preferential glial commitment (particularly toward the oligodendroglial fate) upon injection in immunodeficient MLD mice. Importantly, in mice analyzed at 3 and 6 months after transplant, we observed a small subpopulation of MBP‐expressing hiPS‐NSCs embedded in or close to myelin‐dense areas, suggesting differentiation of hiPS‐NSCs in myelinating oligodendrocytes (Fig. 7I). We detected 4.91% ± 4.46% (*n* = 3) and 4.49% ± 1.74% (*n* = 9) of engrafted hfNSCs and iPS‐NSCs, respectively, expressing the proliferation marker Ki67 (supplemental online Fig. 7A). In line with earlier studies 39, these cells were preferentially located within or close to the neurogenic SVZ region (Fig. 7J), and their proportion was similar over time. Importantly, we did not detect human cells expressing the pluripotency marker NANOG in all hiPS‐NSC‐treated mice. In addition, we did not detect abnormal proliferation or tumor formation in the brain of hiPS‐NSC‐injected mice up to 10 months after transplant. In contrast, extensive teratomas were observed in MLD mice already at 1 month after transplant with undifferentiated parental hiPSC lines (not shown).

We then performed intraventricular transplantation 35, 36 of ND and ARSA‐MLD hiPS‐NSCs in neonatal (PND1) immunodeficient MLD mice (200,000 cells per injection site; bilateral; *n* = 6 mice per group), with the aim of testing an experimental setting that could better challenge the feasibility and safety of hiPS‐NSC transplantation while being more relevant to assess the therapeutic potential of the strategy (supplemental online Table 7; supplemental online Fig. 7C). Mice were analyzed 6 months after transplantation. The yield of cell engraftment was comparable to that measured in mice transplanted at PND60 (8.91% ± 2.24%; *n* = 6; supplemental online Fig. 7A). Engrafted cells displayed a widespread distribution (3,780.0 ± 190.4 µm; *n* = 6; supplemental online Fig. 7A) and a preferential migration toward caudal brain regions, reaching the cerebellum (supplemental online Fig. 7D, 7E). The majority of engrafted hiPS‐NSCs expressed oligodendroglial (GST‐π) and astroglial markers (S100‐β; supplemental online Fig. 7F). Importantly, we did not observe abnormal proliferation, as also indicated by the minimal percentage of donor‐derived cells expressing Ki67 (1.62% ± 0.38%; *n* = 6; supplemental online Fig. 7A).

Engrafted iPS‐NSCs provided a robust source of ARSA enzyme in CNS tissues. At 3 months after transplant, ARSA activity reached ∼30% of physiological levels (assessed in *Rag^−/−^;γ-chain^−/−^;As2^+/+^* mice; *n* = 4) in MLD mice treated at PND60 with ND iPS‐NSCs (*n* = 4) (Fig. 7K). Reconstitution of ARSA activity was consistent throughout the CNS, including regions where few or no engrafted cells were found (supplemental online Fig. 7D). In addition, ARSA activity was stable over time, as assessed in mice transplanted at PND1 (*n* = 3 mice per group) or at PND60 (*n* = 2 mice per group) and analyzed at 6 months after transplant (Fig. 7K). Regardless of the age at transplant or the time of analysis, MLD mice transplanted with ARSA‐overexpressing hiPS‐NSCs (*n* = 2‐4 mice per group) consistently displayed ≥ twofold ARSA activity (∼70% of physiological levels), as compared with MLD mice transplanted with ND iPS‐NSCs (Fig. 7K). These results show that ARSA‐MLD hiPS‐NSCs displayed stable engraftment and a favorable safety profile, providing long‐lasting ARSA enzymatic supply to MLD brains.

### Amelioration of Sulfatide Storage in MLD Mice Transplanted With ARSA‐MLD hiPS‐NSCs

Similar to what observed in immunocompetent MLD mice 53, sulfatide storage (assayed with Alcian blue staining) was detectable in immunodeficient MLD mice at 3 months of age, preferentially in white matter regions, and increased progressively with age (supplemental online Fig. 8). MLD mice transplanted with ARSA‐MLD hiPS‐NSCs showed a significant decrease of the alcianophilic area in white matter regions (corpus callosum, anterior commissure, and hippocampal fimbria) when compared with age‐ and genotype‐matched untreated mice (Fig. 7L, 7M), with an indication of increased sulfatide reduction in mice transplanted at PND1 as compared with those transplanted at PND60.

## Discussion

Here we generated and characterized a population of human iPSC‐derived NSCs that share functional properties and global molecular signature with somatic NSCs isolated from the human fetal brain—the only source of NSCs available so far for clinical testing. In addition, we demonstrated the feasibility of ex vivo gene therapy using patient‐specific ARSA‐overexpressing hiPS‐NSCs for the long‐lasting metabolic correction and clearance of CNS storage in a mouse model homolog of MLD, a severe lysosomal storage disease affecting the nervous system.

The immunomodulation and neuroprotection provided by NSC‐based therapies 54 in animal models of different CNS diseases 10, 11, 12, 34, 55 indicate NSCs as reasonable cell therapeutics for neurodegenerative pathologies 56, 57. The development of effective and safe NSC‐based therapies will depend on procedures that yield well‐characterized and expandable lines suitable for transplantation. Transplantation of clinical‐grade NSCs derived from human fetuses is an accepted and safe technique 14, 58, 59, but is often met with ethical issues and still requires immunosuppression. Thus, efforts are ongoing in investigating alternative cell sources to obtain large numbers of autologous, transplantable, authentic human NSCs that could meet similar quality criteria and bypass concerns.

We optimized a published protocol 18 to generate and expand iPSC‐derived NSCs sharing molecular, phenotypic, and functional identity with hfNSCs 6, 7, which we used as a valuable gold standard in a side‐by‐side comparison when validating the phenotype of the resulting iPSC‐derived NSC populations in vitro and predicting their performance upon intracerebral transplantation. In the first step of the differentiation protocol, we observed the formation of rosette structures typical of early stage ES‐ or iPSC‐derived neural precursors 60, 61 that are progressively lost upon exposure to neural inducers. Ultimately, we obtained a cell population consistently expressing typical hfNSC markers (i.e., Pax6, Nestin, and CD133) 12, 39, as well as markers of long‐term expandable PSC‐derived NSCs (lt‐NES; i.e., ASCL1, AQP4, and S100β) 62, 63. The molecular heterogeneity of hiPS‐NSCs achieved using our protocol likely accounts for their sustained expansion in vitro (up to 10‐15 passages) and the concomitant maintenance of multipotency (capability to generate glial cells, including relevant numbers of oligodendrocytes, as well as different neuronal subtypes), two properties that are often mutually exclusive in more homogeneous, lineage‐committed iPSC‐derived neural populations 61, 62. The downregulation of pluripotency‐ and cancer‐related genes highlighted by phenotypic and genome‐wide transcriptomic analyses further confers to hiPS‐NSCs a robust “NSC signature” that might avoid further enrichment from bulk cultures, a procedure that has been proposed to decrease the tumorigenic risk upon transplantation 64. Accordingly, the integrated viral reprogramming cassette was silenced in hiPSCs and it was not reactivated in hiPS‐NSCs upon differentiation, adding a favorable safety trait.

Lysosomal storage diseases are promising candidates for cell and/or gene therapy strategies, because of the peculiar characteristics of intercellular and intracellular sorting and trafficking of lysosomal enzymes 65, which can be secreted by donor cells and mediate efficient cross‐correction in all CNS compartments. This rationale is at the basis of GT approaches that have been evaluated in preclinical models of several LSDs 66, 67, including globoid cell leukodystrophy (GLD) 68 and MLD 69. Supraphysiological levels of ARSA activity in CNS tissues provided through autologous genetically modified HSCs are required to halt disease progression in presymptomatic children with late infantile MLD 30, a LSD for which allogeneic hematopoietic stem cell transplantation failed to prove unambiguous benefit 32. Similarly, transplantation of enzyme‐overexpressing NSCs ensures higher enzymatic activity in CNS tissues of LSD mice, enhancing therapeutic benefit 9, 35, 55. Based on this rationale, we generated ARSA‐overexpressing MLD hiPS‐NSCs by differentiating MLD hiPSC clones that had been previously transduced with lentiviral vectors carrying a functional *hARSA* gene. Importantly, we used a lentiviral vector carrying the codon‐optimized *hARSA* coding sequence that was developed for the HSC GT clinical trial in MLD patients 30, 70. Contrary to a previous report 46, we did not experience LV‐mediated toxicity in hiPSCs, which were viable and functional for several passages after transduction and expressed stable supraphysiological ARSA activity. Despite clonal selection of hiPSCs and integration, site analyses would be required for stringent quality controls in the perspective of clinical testing. In this study, efficient enzymatic correction was achieved with low numbers of copies of integrated vectors, pointing to a reduced genotoxic risk. Indeed, VCN ranging from 1 to 3 have been reported in autologous HSCs transduced with the LV.hARSA used in the MLD clinical trial, with no indication of clonal expansion after 6‐year follow‐up 30, 70.

The yield of hiPS‐NSC engraftment in the CNS of MLD mice was highly variable (1%–25%) and apparently not dependent from the cell type (ND and ARSA‐overexpressing MLD) or the age at treatment (adult or neonate). Also, the average yield of iPS‐NSC engraftment was in the range of that observed for hfNSCs in this and earlier studies 36, 39. The majority of engrafted cells quickly lost proliferation ability and immature phenotype, showing robust oligodendroglial commitment and terminal differentiation, as demonstrated by MBP expression.

Engrafted hiPS‐NSCs provided robust, widespread, and stable distribution of a functional ARSA enzyme in CNS tissues of adult‐ and neonatal‐transplanted MLD mice, supporting the occurrence of enzyme circulation in the CSF and cross‐correction 38. We documented reduced sulfatide storage in all MLD mice transplanted with ARSA‐overexpressing MLD hiPS‐NSCs. The clear advantage observed in those mice that received neonatal treatment as compared with those transplanted in the adult age was likely the consequence of delayed sulfatide accumulation provided by precocious enzymatic supply. The sulfatide clearance observed in densely engrafted regions likely relied on the local supply of functional enzyme available for cross‐correction of surrounding affected cells. In the perspective of clinical development, this effect might be enhanced by increasing the ratio between engrafted and host cells. Multiple intracerebral cell injections delivering up to 10^9^ purified hNSCs 7 are feasible and apparently safe in LSD patients 13. Accurate preclinical studies should assess the safety profile upon transplantation of comparable numbers of hiPS‐NSCs (normalized to the brain volume of the animal model tested). Ex vivo GT leading to enzyme overexpression might conceivably provide similar or enhanced therapeutic benefit with lower numbers of transplanted cells.

## Conclusion

We show here that MLD patient‐specific hiPSCs can be safely and efficiently engineered to express supraphysiological levels of the ARSA enzyme, differentiate into hfNSC‐like neural stem cells, and may serve as autologous source for stable ARSA supply and amelioration of sulfatide storage in MLD‐affected brains. Compared with recent studies that assessed the potential benefit of ARSA‐overexpressing iPSC‐derived NSCs 26, 46 and lt‐NES‐derived astroglial progenitors 26 in LSD mice, our work presents several lines of novelty: (a) We validated for the first time bona fide hiPS‐NSCs in a side‐by‐side comparison with their somatic counterpart, which is currently under clinical testing; (b) we characterized four hiPSC clones from two MLD patients, demonstrating consistent and efficient gene transfer and ARSA overexpression using a clinically relevant vector 30; and (c) differently from lt‐NES, ARSA‐MLD hiPS‐NSCs maintain their myelinogenic potential in vivo, upon neonatal and adult transplantation. All these features are relevant for clinical development of cell‐based approaches because CNS pathology in MLD would potentially benefit from transplantation of a cell type that could provide both enzymatic reconstitution and replacement of damaged or lost cells.

Our in vivo data obtained in the adult transplantation setting as well as in the more challenging setting (in terms of feasibility and safety) represented by neonatal transplantation strongly point against the potential tumorigenicity of hiPS‐NSCs. In addition, the particular design of the reprogramming LV used here allowed for efficient and specific excision of the reprogramming cassette without compromising iPSC functions or expression of the therapeutic gene. The generation of insertion‐free hiPSCs that could be differentiated into bona fide hiPS‐NSCs will facilitate the clinical development of the strategy.

Given the time‐ and region‐dependent CNS and PNS involvement that characterize MLD and similar LSDs, combined approaches that could ensure efficient enzyme delivery to all affected tissues at due time will be needed to prevent/counteract disease progression. We have recently shown that NSC GT synergizes with HSC transplants, providing dramatic extension of lifespan and global clinical‐pathological rescue in a murine model of GLD 36, a LSD sharing several clinical‐pathological features with MLD, suggesting NSCs as a cellular model potentially suitable for combined ex vivo gene therapy strategies.

## Author Contributions

V.M. and G.F.: conception and design, collection and/or assembly of data, data analysis and interpretation, manuscript writing; D.S., S.D.C., C.C., M.P., F.M., and S.G.: collection and/or assembly of data; M.L., W.M., F.S., A.V., A.B., and S.M.: collection and/or assembly of data, data analysis and interpretation; V.B.: data analysis, scientific discussion, critical review of the manuscript; A.G.: conception and design, data analysis and interpretation, financial support, manuscript writing, final approval of manuscript.

## Disclosure of Potential Conflicts of Interest

The authors indicated no potential conflicts of interest.

## Supporting information

Supporting Information
