# Supplementary material for: Generation of Human Induced Pluripotent Stem Cell‐Derived Bona Fide Neural Stem Cells for Ex Vivo Gene Therapy of Metachromatic Leukodystrophy
Source: Stem Cells Transl Med. 2016 Sep 16;6(2):352–68. doi: 10.5966/sctm.2015-0414 (PMC5442804; doi:10.5966/sctm.2015-0414)
Supplement: Supplementary file 1 — Supporting Information [file SCT3-6-352-s001.pdf]

<u>Supplemental methods</u>

Cell culture media

<u>Human fibroblasts medium</u> (**HFM**): DMEM (Life Technologies), 10% Fetal Bovine Serum (Euroclone), 0.1 mM nonessential amino acids (Life Technologies), 1 mM Sodium Piruvate (Life Technologies), 2 mM L-Glutamine (Lonza), 100 U/ml penicillin/streptomycin (Lonza).

<u>hiPSC medium</u> (hiPSCM): DMEM/F12 (Gibco), 20% Knock-out serum replacement (Life Technologies), 0.1 mM non-essential amino acids, 1 mM Sodium Piruvate, 0.1 mM β-mercaptoethanol (Life Technologies), 2 mM L-Glutamine, 100 U/ml penicillin/streptomycin, 10 ng/ml of FGF2.

<u>EB medium</u>: DMEM/F12, 20% Knock-out serum replacement, 0.1 mM non-essential amino acids, 1 mM Sodium Piruvate, 0.1 mM β-mercaptoethanol, 2 mM L-Glutamine, 100 U/ml penicillin/streptomycin.

<u>N2 medium</u>: Neurobasal Medium (Invitrogen), 1x N2 supplement (Invitrogen), 2 mM L-Glutamine, 100 U/ml penicillin/streptomycin].

<u>BASF medium</u>: N2 medium supplemented with 20 ng/ml SHH, 100 ng/ml FGF8, 20 ng/ml Brain-Derived Neurotrophic Factor (BDNF, PeproTech), and 0.2 mM Ascorbic Acid (AA, Sigma-Aldrich).

<u>BAS medium:</u> N2 medium containing BDNF, AA and SHH.

<u>hiPS-NSC proliferation medium</u> (NPM): N2 medium supplemented with 20 ng/ml of FGF2 and 20 ng/ml of epidermal growth factor (EGF, PeproTech).

<u>Neuronal differentiation medium</u> (NDM): DMEM/F12-Neurobasal medium (1:1) mixture supplemented with 0.5x N2 supplement, 0.5x B27 supplement (Invitrogen), 2 mM L-Glutamine, 100 U/ml penicillin/streptomycin, 2.5 μg/ml insulin (Sigma-Aldrich), 100 μM 2-mercaptoethanol, 0.1 mM non-essential amino acids.

<u>Glial differentiation medium</u> (GDM): DMEM/F12, with 100 ng/ml Biotin (Sigma-Aldrich), 1 μM dibutyryl cyclic AMP (Sigma-Aldrich), 10 ng/ml insulin growth factor 1 (IGF1; PeproTech), 60 ng/ml triiodothyronine (T3; Sigma-Aldrich), 10 ng/ml PDGFAA, 10 ng/ml NT3.

<u>Glial maturation medium (GMM</u>): GDM medium without PDGFAA and NT3

<u>hfNSC medium</u>: serum-free, mitogen-supplemented DMEM/F12 medium [1].

Karyotype analyses

hiPSC cultures grown in feeder-free conditions were treated with KaryoMAX colcemid (0.1 mg/ml, Life Technologies) for 2 h at 37°C. After hypotonic treatment with 0.075 M KCl and fixation in methanol:acetic acid (3:1 v/v), cell suspension was dropped onto a slide and air dried. Chromosome counts and G-banding karyotype analyses were done on metaphases stained with Vectashield mounting medium with DAPI (Vector Laboratories). Multicolor FISH analyses were performed by using SKY paint probe mixtures (Applied Spectral Imaging, Santa Clara, CA) according to the manufacturer instructions [2]. Images were captured using an Olympus BX61 Research Microscope equipped with a cooled CCD camera and analyzed with Applied Imaging Software CytoVision (CytoVision Master System).

Embryoid body assay

hiPSC colonies (80% confluence) were detached using dispase and plated in Ultralow attachment plates (Corning) in EB medium for 10 days. After that EBs were plated on matrigel-coated dishes for additional 10 days and then fixed with 4% paraformaldehyde or detached and collected for IF and RNA extraction, respectively.

Teratoma assay

hiPSC colonies were dissociated by using accutase and suspended in hiPSCM. Cells were then mixed 1:1 with undiluted matrigel on ice and injected subcutaneously into immunodeficient NSG mice. Teratomas were allowed to grow till reaching a size of ≥ 1 cm^3 (4–8 weeks), isolated by dissection and fixed in formalin. Fixed tissues were embedded in paraffin, sectioned at 10μm thickness and stained with hematoxylin and eosin (H&E).

Immunocytochemistry

Fixed cells and tissues (free-floating vibratome-cut sections) were rinsed with PBS, incubated in blocking solution (10% NGS + 0.1% Triton X-100 in PBS) for 30-60 minutes and then overnight at 4°C with primary antibodies. Samples were then incubated for 1h at RT with species-specific fluorophore-conjugated secondary antibodies (PBS +1% NGS). Primary and secondary antibodies are listed in **Supplemental Table 8.** Nuclear counterstaining was performed with ToPro-3 (Invitrogen) or DAPI (Roche). Coverslips and tissue sections were mounted on glass slides using Fluorsave (Calbiochem). No detectable signal was observed in samples in which the primary antibodies were omitted. Samples were visualized with Zeiss Axioskop2 microscope using double laser confocal microscopy with Zeiss Plan-Neofluar objective lens (Zeiss, Arese, Italy). Images were acquired using a Radiance 2100 camera (Bio-Rad, Segrate, Italy) and LaserSharp 2000 acquisition software (Bio-Rad) at 40x with oil magnification.

Evaluation of the Vector Copy Number (VCN)

Genomic DNA (gDNA) was extracted from cell pellets (1-2x10^6 cells) by using Maxwell 16 Cell DNA Purification Kit (Promega) following the manufacturer instructions. gDNA was quantified by 260/280 nm OD reading on the NanoDrop ND-1000 Spectrophotometer. VCN were quantified by Taqman analysis in Optical 96-well Fast Thermal Cycling Plates (Applied Biosystem) on ABI PRISM 7900 Sequence Detector System (Applied Biosystem) by using Universal PCR Master Mix (Applied Biosystems). For each reaction, 100 ng of gDNA and probe+primers mixture of custom TaqMan Gene Expression Assays (Applied Biosystems) were used. LV.OSK and LV.hARSA integrated backbone have been detected by using the following set of primers and probe: forward primer, 5′-TACTGACGCTCTCGCACC-3′ at 300nM final concentration; reverse primer, 5′-TCTCGACGCAGGACTCG-3′ at 300nM final concentration; probe 5′-FAM-ATCTCTCTCCTTCTAGCCTC-MGBNFQ-3′ at 200nM final concentration. bdLV.hARSA.GFP integrated backbone has been detected by using the following set of primers and probe annealing GFP sequence: forward primer, 5′-CAGCTCGCCGACCACTA-3′ at 300nM final concentration; reverse primer, 5′-GGGCCGTCGCCGAT-3′ at 300nM final concentration; probe 5′-FAM-CCAGCAGAACACCCCC-MGBNFQ-3′ at 200nM final concentration. As internal reference for normalization, we amplified a fragment of the human telomerase gene detected by

using the following set of primers and probe: forward primer, 5′-GGCACACGTGGCTTTTCG-3′ at 300nM final concentration; reverse primer, 5′-GGTGAACCTCGTAAGTTTATGCAA-3′ at 300nM final concentration; probe 5′-VIC-TCAGGACGTCGAGTGGACACGGTG-TAMRA-3′ at 200nM final concentration. For standard curve, gDNA was extracted from a human lymphoblast cell line carrying 1 copy of integrated LV.GFP, validated by Southern blot analysis. The standard curve, based on different dilutions of DNA (from 0.32 to 40 ng) and, accordingly, of LV copies was used as standard for LV, GFP and telomerase amplification. The SDS 2.2.1 software was used to analyze data. Experiments were run in triplicated for at least n=3 experiments. VCN was calculated as follow: VCN = (ng LV/ng endogenous DNA) × (number of LV integration in the standard curve) x sample ploidy.

Bisulfite Sequencing

Genomic DNA (gDNA) was extracted from cellular pellets (2-3 x 10^6 cells) using QiAMP DNA Blood Mini Kit (Qiagen, cat. 51104) following the manufacturer instructions and quantified by 260/280nm OD reading. gDNA bisulfite conversion of unmethylated Cytosine was performed with EpiTect Bisulfite kit (Qiagen, cat. 59104) following the manufacturer instructions. Bisulfite converted NANOG and OCT4 endogenous promoter regions were amplified by PCR using Taq DNA Polymerase (Roche) without proof-reading activity. For each reaction 100 ng of gDNA and 1µM of custom primer were used (primers recognizing bisulfite converted gDNA are listed on **Supplemental Table 9**). PCR products are then cloned into One Shot TOP10 Chemically Competent E.Coli cells (Invitrogen) with TOPO TA Cloning Kit (Life Technologies, cat. 450640) using pCRTM II-TOPO Vector plasmid with single 3′-thymidine (T) overhangs for TA cloning, according to the manufacturer instructions. Selected colonies (n=10 per PCR product) were picked and let grown. Plasmid containing the PCR product was extracted with Wizard Plus SV Minipreps DNA Purification System (Promega, cat. A1330) according to the manufacturer instructions, digested with 20U/µg DNA EcoRI (NewEngland Biolabs) and resolved on agarose gel to assess PCR product integration. The plasmid was sequenced (PrimmBiotech) by using M13 primers flanking the PCR product. Raw sequences were then analyzed with BiQ Analyzer (Max Planck Institute).

Gene expression studies

mRNA was extracted from cell pellets (1-2x10^6 cells) with RNeasy mini Kit (Qiagen, cat. 74104) according to the manufacturer instructions. Quantification of mRNA was determined by 260/280 nm OD reading on the NanoDrop ND-1000 Spectrophotometer. Reverse transcriptase reactions were performed by using QuantiTect reverse transcription kit (Qiagen), according to the manufacturer instructions.

RT-PCR analyses were performed by using FastStart™ Taq DNA Polymerase PCR KIT (Roche, cat. 04738381001). For each reaction, 100 ng of template cDNA and 1mM of primers (reported in **Supplemental Table 10**) were used. PCR products were then resolved by electrophoresis in a 1.5% agarose gel.

Taqman analyses were performed in Optical 96-well Fast Thermal Cycling Plates on ABI PRISM 7900 Sequence Detector System by using Universal PCR Master Mix. For each reaction, 100 ng of template cDNA and 1x probe+primers mixture of custom or commercial TaqMan Gene Expression Assays (Applied Biosystems) were used. LV.OSK expression has been detected by using the following set of primers and probe: forward primer, 5′- GGACTGATGGGCAAGTTCGT-3′ at 300 nM final concentration; reverse primer, 5′-GCCCTTGGACACGCTGAT-3′ at 300 nM final concentration; probe 5′-FAM- TCAGGCTGGCTTTCAG-MGBNFQ-3′ at 200 nM final concentration. Human OCT4 and ARSA expression were detected by using Hs03005111_g1 and Hs04185629_g1 TaqMan Gene Expression Assays, respectively. As internal reference for normalization, we amplified a fragment of the human HPRT1 mRNA by using Hs01003267_m1 TaqMan Gene Expression Assays. The SDS 2.2.1 software was used to analyze data. Experiments were run in triplicate for at least n=3 experiments.

Sybr green analyses were performed in Optical 96-well Fast Thermal Cycling Plates on ViiA™ 7 Real-Time PCR System (Applied Byosistem) by using Power SYBR Green PCR Master Mix (Applied Byosistem, cat. 4367659). For each reaction, 10 ng of template cDNA and 10 µM of custom primers were used.

As internal reference for normalization GAPDH was used. Data were analyzed with ViiA™ 7 Software. Experiments were run in duplicated for n=3 experiment. Primers used for Sybr Green qRT-PCR analyses are listed in **Supplemental Table 11.**

Microarrays

Raw Affymetrix GeneChip Human Genome 1.0 ST Array data were preprocessed using Robust Multichip Analysis (RMA) [3] implemented in R package oligo v1.30.0 [4] in R statistical environment v3.1.3 [5]. The sets of probes were mapped to gene symbols with the hugene10sttranscriptcluster.db annotation package. Hierarchical clustering and multidimensional scaling of samples were performed on 5000 genes with the largest variance, using functions in R packages stats v3.3.0 and gplots v2.17.0. In order to identify differentially expressed genes, a linear model was fitted to the normalized data, and the empirical Bayes moderated t-statistics were obtained using the R package limma v3.6.9 [6] [7]. Genes with fdr-controlled p-value<0.05 were regarded as differentially expressed. Groups of genes found to be differentially expressed were analyzed for functional enrichment with DAVID Functional Annotation Tool [8, 9]

Cre-recombinase-mediated excision of the reprogramming cassette

Single cell-derived hiPSCs were incubated overnight with 150ng p24/µl of integrase-defective LV (IDLV) expressing the Cre-recombinase from the EF1[illegible] promoter in hiPSCM + 10µM Y27632 (Sigma). Single cell-derived clones were then expanded. Copies of integrated LV genome and expression of selected genes were assess by qPCR on genomic DNA and RNA.

Western blot

Protein extracts from cellular pellets (2-3 x 10^6 cells) were obtained as previously described [10]. 20 µg of samples were prepared and loaded on 4-12% Novex NuPAGE SDS-PAGE system according to manufacturer instructions. The PVDF membranes were stained with rabbit polyclonal anti-GFP antibody (1:2000; A11122, Molecular Probes; expected MW: 30 kDa), rabbit polyclonal anti-HA antibody (1:1000; Ab-13834-100, AbCAM), goat polyclonal anti-ARSA antibody (1:1000 PAB7518, ABNOVA expected MW: 53 kDa); and rabbit polyclonal IgG anti-Calnexin antibody (1:3000; C4731, Sigma Aldrich; expected MW: 90 kDa), revealed by HRP-conjugated goat anti-rabbit (1:10.000; AP132P, Chemicon), chicken anti-goat (1:10 000; sc2153, Santa Cruz) and incubated with chemiluminescent substrate (EMD Millipore Immobilion Western Chemiluminescent HRP Substrate).

Quantification of ARSA activity

In cellular protein extracts, ARSA activity was determined by p-nitrocatechol assay, as described [11]. hiPSC lines were grown and expanded on matrigel-coated dishes (feeder-free conditions) in hiPSCM + 10μM Y27632 (Sigma) for at least 12 days before the analyses. In tissue protein extracts, ARSA activity was determined by using the 4-methylumbelliferyl-sulfate substrate, as described [10, 12].

Flow cytometry

hiPSC colonies were manually picked on matrigel-coated dishes and expanded for one passage in hiPSCM + 10μM Y27632 (Sigma). hiPSCs and hiPS-NSCs were detached with accutase, whereas hfNSC neurospheres were mechanically disgregated to single-cell suspension by pipetting. Cells were centrifuged at 600 x g for 5 min and collected for FACS analyses. For FACS sorting of GFP-positive cells, cells were analyzed by using an argon laser excitation at 488 nm and emission at 530 nm. Untransduced cells were used as negative control to define the gate strategy (SSC, FSC, laser intensity parameters). For flow cytometry analyses the following antibodies were used: anti-CD133 PE-conjugated antibody (130-080-801, Miltenyi Biotech), anti-NGFR APC-conjugated antibody (560326 BD Pharmigen), anti-SSEA4 APC-conjugated antibody (FAB1435a, R&D Systems).

Analysis of sulfatide storage

Histochemical staining of sulfatides in brain tissues was performed using the cationic dye Alcian Blue (A-5268, Sigma-Aldrich, Germany). Briefly, 40μm fixed brain slices were post-fixed for 3h with a mix of 2% paraformaldehyde/3% glutaraldehyde, and then incubated with the dye (0.025% dye in 0.025M sodium acetate buffer, pH 5.7, containing 0.3M $MgCl_2$ and 2.5% glutaraldehyde) for 3 hours. After thorough rinsing with PBS, specimens were mounted on glass coverlips with glycerol:PBS (1:1).

For quantification of sulfatide storage, four pictures for each selected brain region (corpus callosum, anterior commissure and hippocampal fimbria) were sequentially acquired at 20X magnification (threshold defined on the signal of WT tissues). The percentage of alcianophilic areas over the total image area was quantified on selected images using the ImageJ software.

References

1. Neri M, Maderna C, Ferrari D et al. Robust generation of oligodendrocyte progenitors from human neural stem cells and engraftment in experimental demyelination models in mice. PLoS ONE. 2010;5:e10145.
2. Paulis M, Castelli A, Susani L et al. Chromosome transplantation as a novel approach for correcting complex genomic disorders. Oncotarget. 2015.
3. Irizarry RA, Hobbs B, Collin F et al. Exploration, normalization, and summaries of high density oligonucleotide array probe level data. Biostatistics. 2003;4:249-264.
4. Carvalho BS, Irizarry RA. A framework for oligonucleotide microarray preprocessing. Bioinformatics. 2010;26:2363-2367.
5. Dean CB, Nielsen JD. Generalized linear mixed models: a review and some extensions. Lifetime Data Anal. 2007;13:497-512.
6. Smyth GK. Linear models and empirical bayes methods for assessing differential expression in microarray experiments. Stat Appl Genet Mol Biol. 2004;3:Article3.
7. Ritchie ME, Phipson B, Wu D et al. limma powers differential expression analyses for RNA-sequencing and microarray studies. Nucleic acids research. 2015;43:e47.
8. Huang da W, Sherman BT, Lempicki RA. Systematic and integrative analysis of large gene lists using DAVID bioinformatics resources. Nature protocols. 2009;4:44-57.
9. Huang da W, Sherman BT, Lempicki RA. Bioinformatics enrichment tools: paths toward the comprehensive functional analysis of large gene lists. Nucleic acids research. 2009;37:1-13.
10. Lattanzi A, Neri M, Maderna C et al. Widespread enzymatic correction of CNS tissues by a single intracerebral injection of therapeutic lentiviral vector in leukodystrophy mouse models. Human molecular genetics. 2010;19:2208-2227.
11. Biffi A, Montini E, Lorioli L et al. Lentiviral hematopoietic stem cell gene therapy benefits metachromatic leukodystrophy. Science. 2013;341:1233158.
12. Martino S, Consiglio A, Cavalieri C et al. Expression and purification of a human, soluble Arylsulfatase A for Metachromatic Leukodystrophy enzyme replacement therapy. J Biotechnol. 2005;117:243-251.

Supplemental Figures

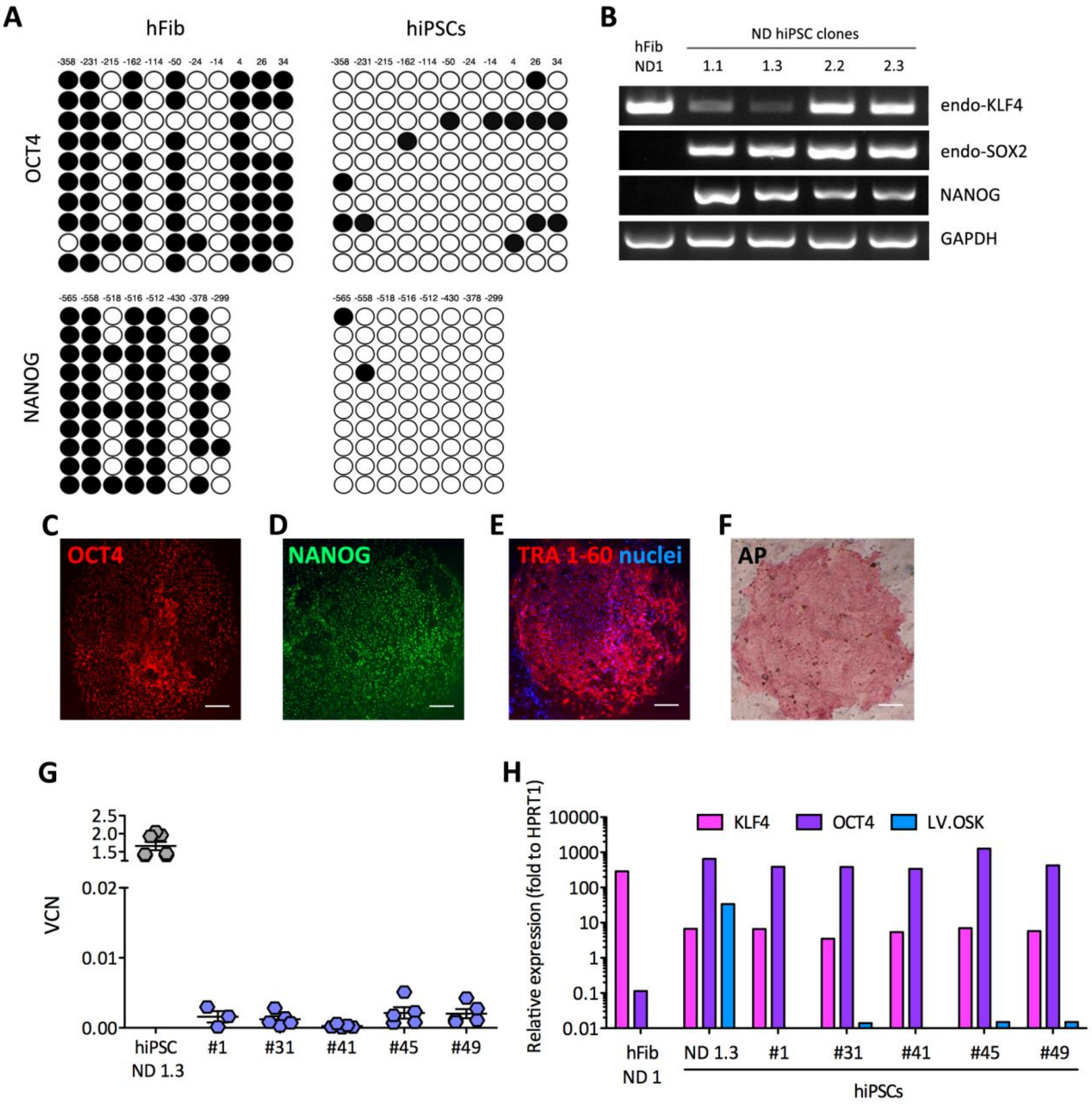

Figure S1. Characterization of hiPSCs from normal donor fibroblasts. (**A**) The methylation state of OCT4 and NANOG promoters was analyzed in hiPSCs and parental fibroblasts by bisulfite sequencing. Representative diagrams of hiPSC ND1.1 clone and its parental fibroblast cell line are reported. Columns indicate CpG islands (numbers refer to the position of the CpG island from the TSS)[1] analyzed in each cloned PCR product (rows). Ten PCR products for each cell line were analyzed. Open and filled circles correspond to unmethylated and methylated CpG islands, respectively. (**B**) mRNA expression levels of NANOG and endogenous SOX2 (assessed by RT-PCR) in hiPSC clones and one fibroblast cell line. Endogenous KLF4 is expressed in both cell types. (**C-E**) Representative immunofluorescent pictures showing cells expressing OCT4 (red; C), NANOG (green; D) and TRA 1-60 (red; E) in hiPSC clones. Nuclei were counterstained with DAPI (blue). (**F**) Representative bright-field picture showing alkaline phosphatase (AP) activity in hiPSCs. **(G**) Copies of integrated LV.OSK genome/host genome (vector copy number, VCN; assessed by qRT-PCR) in hiPSC clone ND1.3 and in 5 clones (#1, #31, #41, #45, #49) derived from ND1.3 after Cre-recombinase treatment. Data are expressed as the mean±SEM, n=2 replicates from 2-3 samples at different subculturing passages. (**H**) mRNA expression levels (by qRT-PCR) of OCT4 and KLF4 and of the reprogramming cassette (LV.OSK) assessed in Cre-recombinase treated clones as compared to untreated hiPSC clone ND1.3 and parental fibroblasts (hFib). Scale bars: 20µm (C, D, E); 400 µm (F).

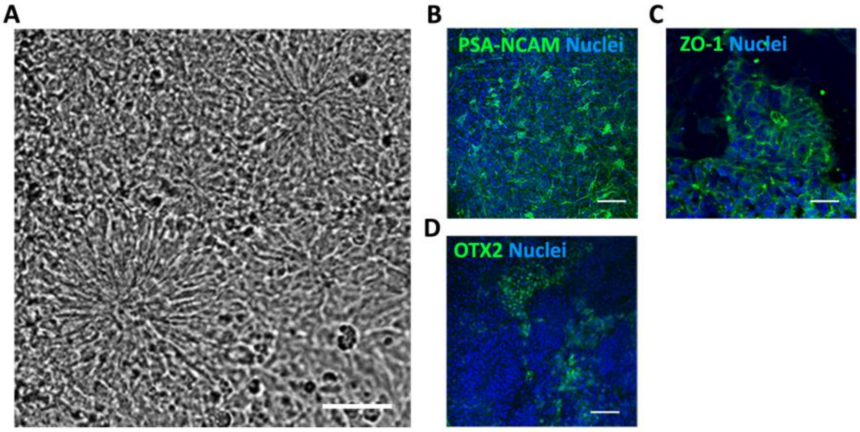

Figure S2. Formation of rosette-like structures during hiPSC to neural transition. (**A**) Bright-field image of radial structures resembling rosettes expressing the neuronal marker PSA-NCAM (**B**), the marker for adherent junctions ZO1 (**C**) and the anterior CNS marker OTX2 (**C**). In B-D nuclei are counterstained with ToPro-3 (blue). Scale bars: 30 µm (A); 50µm (B); 10µm (C, D).

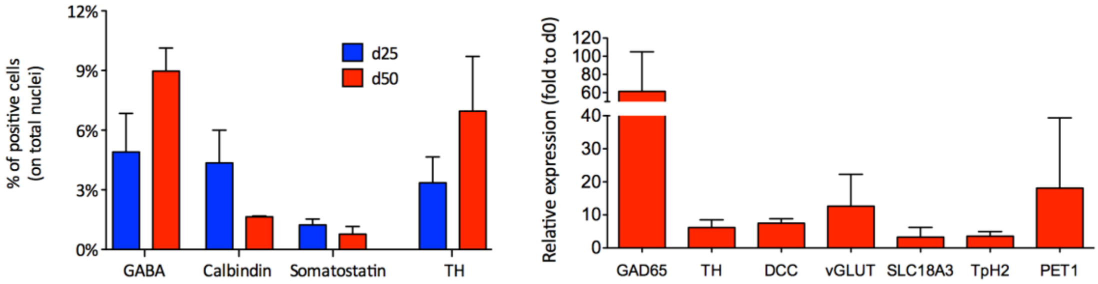

Figure S3. hiPS-NSC-derived neurons express GABAergic and dopaminergic markers. (**A**) Percentages of cells expressing GABA, Calbindin, Somatostatin and the dopaminergic marker Tyrosine Hydroxylase (TH) assessed by immunofluorescence analysis in hiPS-NSC cultures differentiated in NDM conditions for 25 and 50 days (d25, d50). Data are expressed as mean ± SEM; n= 2 hiPS-NSC lines; 2-3 independent experiments in triplicate. (**B**) mRNA expression (assessed by qRT-PCR) of GABAergic (glutamate decarboxylase, GAD65), dopaminergic (TH and deleted in colorectal cancer, DCC), glutamatergic (vesicular glutamate transporter, vGLUT), cholinergic (Solute Carrier family 18 member 3, SLC183A) and serotoninergic (Tryptophan Hydroxylase 2, TpH2; PET1) in hiPS-NSC cultures differentiated in NDM conditions for 50d. Data are expressed as fold to undifferentiated hiPS-NSCs (d0). Mean ± SEM; n= 4 hiPS-NSC clones; 4 independent experiments.

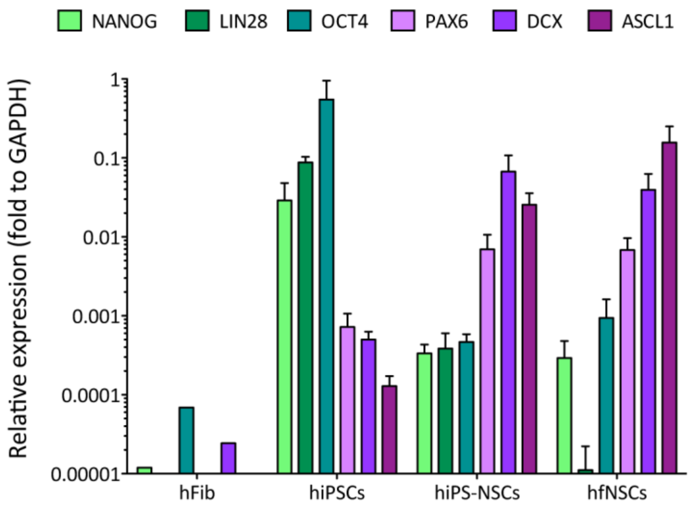

Figure S4. Differential expression of pluripotency and neural markers in normal donor hiPS-NSCs and parental hiPSCs. Transition from hiPSCs to hiPS-NSCs is characterized by downregulation of mRNA expression levels of pluripotency-associated genes (NANOG, LIN28, OCT4) and upregulation of neural stem/progenitor-associated genes (PAX6, DXC, ASCL1) to levels comparable to those detected in allogeneic hfNSCs (by qRT-PCR). Fibroblasts (hFib) are included as control. Data are expressed as the mean±SEM; n= 4 hiPSC clones and correspondent hiPS-NSC lines. PAX6, Paired box 6; DCX, Doublecortin; ASCL1, Achaete-scute homolog1.

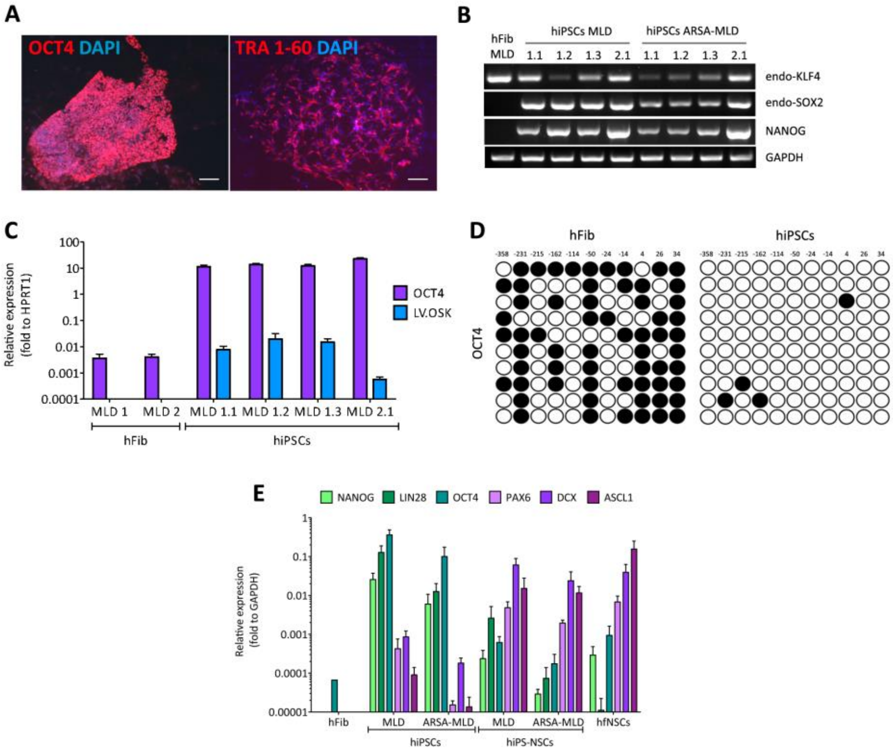
A
OCT4 DAPI
TRA 1-60 DAPI
B
hFib MLD
hiPSCs MLD
hiPSCs ARSA-MLD
1.1 1.2 1.3 2.1 1.1 1.2 1.3 2.1
endo-KLF4
endo-SOX2
NANOG
GAPDH
C
Relative expression (fold to HPRT1)
OCT4
LV.OSK
MLD 1
MLD 2
MLD 1.1
MLD 1.2
MLD 1.3
MLD 2.1
hFib
hiPSCs
D
hFib
hiPSCs
OCT4
-358 -231 -215 -162 -114 -50 -24 -14 4 26 34
E
NANOG LIN28 OCT4 PAX6 DCX ASCL1
Relative expression (fold to GAPDH)
hFib
MLD
ARSA-MLD
MLD
ARSA-MLD
hfNSCs
hiPSCs
hiPS-NSCs

Figure S5. *In vitro* characterization and LV-mediated transduction of MLD hiPSCs. (**A**) Cells expressing OCT4 and TRA1-60 (red) in hiPSC clone MLD1.1 as assessed by indirect immunofluoresecence analysis. Nuclei were counterstained with DAPI (blue). Scale bar: 50µm. (**B**) mRNA expression of NANOG and endogenous SOX2 (assessed by RT-PCR) in four MLD hiPSC clones before and after gene transfer; ARSA-MLD iPSC) and in one parental fibroblast cell line. Expression of KLF4 is present in both cell types. (**C**) Bar graph showing mRNA expression (assessed by qPCR) of endogenous OCT4 and of the reprogramming cassette (LV.OSK) assessed in MLD1 and MLD2 fibroblasts (hFib) and relative hiPSC clones. Data are normalized on HPRT1 expression (housekeeping gene) and are expressed as the mean±SEM (n=3 experiments in triplicates). (**D**) The methylation state of the OCT4 promoter was analyzed in MLD hiPSC clones and parental fibroblasts by bisulfite sequencing. Representative diagrams of hiPSC MLD1.1 clone and parental fibroblasts are shown. Columns indicate CpG islands (the numbers refer to the position of the CpG island from the TSS) analyzed in each cloned PCR products (rows). Ten PCR products for each cell line were analyzed. Open and filled circles correspond to unmethylated and methylated CpG islands, respectively. (**E**) Downregulation of mRNA expression (assessed by qRT-PCR) of pluripotency-associated genes (NANOG, LIN28, OCT4) and upregulation of neural stem/progenitor-associated genes (PAX6, DXC, ASCL1), which show levels comparable to those detected in hfNSCs, upon differentiation of in MLD hiPSCs clones (before and after gene transfer; ARSA-MLD) into hiPS-NSCs. Fibroblasts (hFib) are included as control. Data are expressed as the mean±SEM; n= 4 hiPSC clones/group and correspondent hiPS-NSC lines.

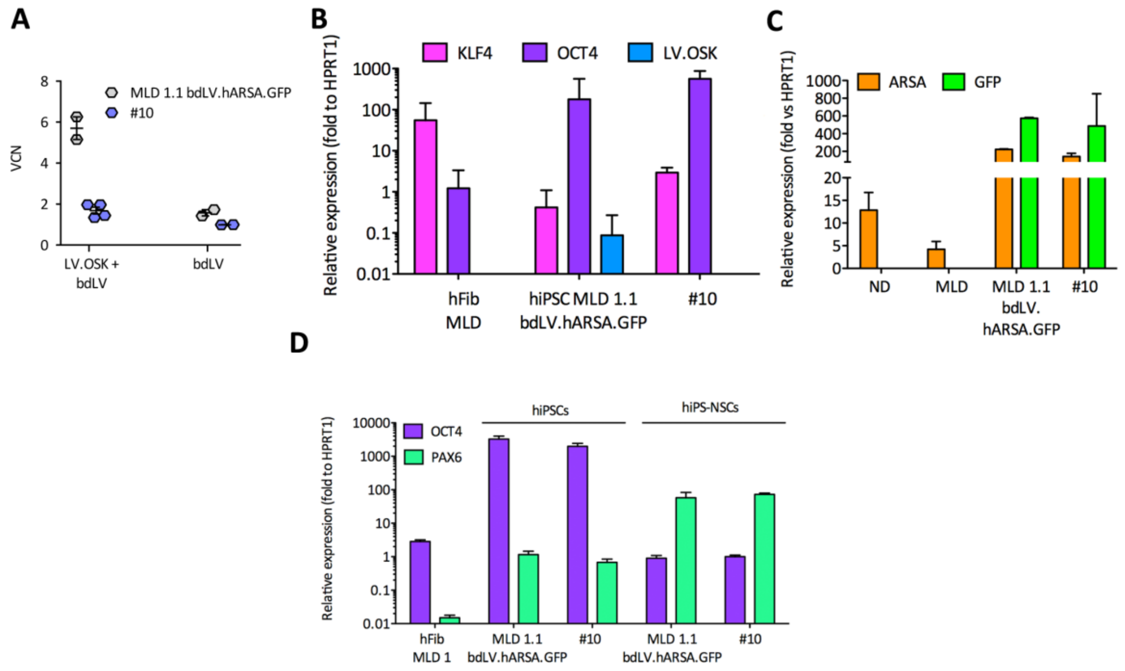

Figure S6. **Cre-mediated excision of the reprogramming cassette does not affect iPSC pluripotency and ARSA over-expression.** (**A**) Copies of integrated LV genome/host genome (vector copy number, VCN) in clone MLD1.1 transduced with bdLV.hARSA.GFP (MLD1.1$^{bdLV.hARSA.GFP}$) and in one clone (#10) derived from MLD 1.1$^{bdLV.hARSA.GFP}$ after Cre-recombinase treatment. The dot plot shows VCN of the therapeutic bdLV.hARSA.GFP (bdLV) and the total VCN (LV.OSK+bdLV.hARSA.GFP). n=2 experiments with samples at two different subculturing passages**. (B)** mRNA expression (by qRT-PCR) of the pluripotency genes OCT4 and KLF4 and of the reprogramming cassette (LV.OSK) assessed in Cre-recombinase-treated clone #10, untreated clone MLD 1.1$^{bdLV.hARSA.GFP}$ and parental fibroblasts. **(C)** Similar GFP and ARSA mRNA expression (assessed by qRT-PCR) in Cre-recombinase-treated clone #10 and untreated clone MLD 1.1$^{bdLV.hARSA.GFP}$. mRNA levels in untransduced/untreated MLD and ND iPSC clones are shown for comparison**.** Data in B and C are expressed as the mean±SEM; n=2 experiments. **(D)** Downregulation of mRNA expression (assessed by qRT-PCR) of the pluripotency master regulator OCT4 and upregulation of the neural marker PAX6 during iPSC to neural transition (iPS-NSC) in untreated and Cre-recombinase-treated hiPSCs. Fibroblasts (hFib) are included as control. Data are expressed as the mean±SEM; n=2 experiments with samples at two different subculturing passages.

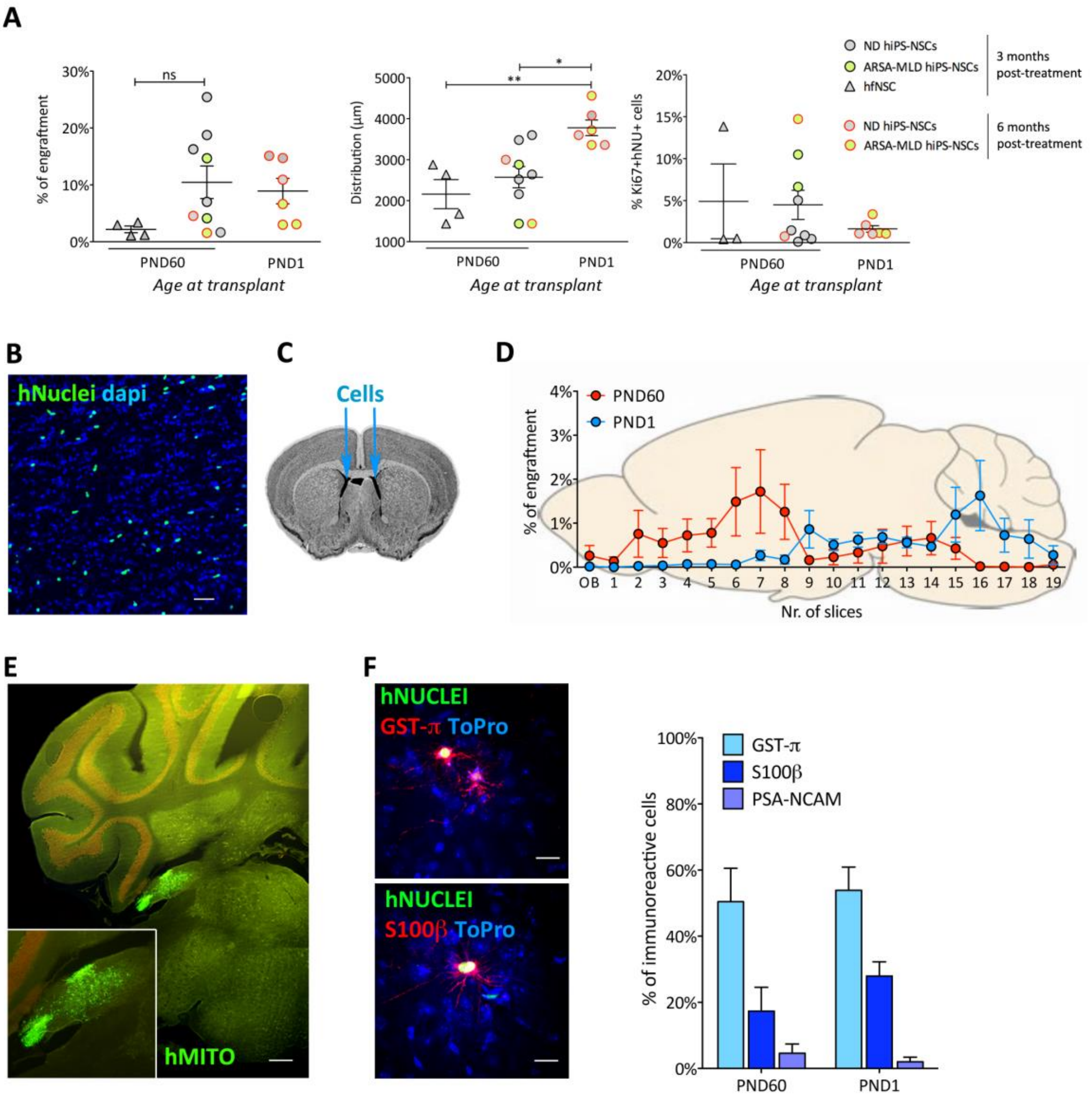

A
ns
% of engraftment
PND60
PND1
Age at transplant
*
**
Distribution (µm)
% Ki67+hNU+ cells
ND hiPS-NSCs
ARSA-MLD hiPS-NSCs
hfNSC
3 months post-treatment
6 months post-treatment
B
hNuclei dapi
C
Cells
D
Nr. of slices
E
hMITO
F
hNUCLEI
GST-π ToPro
S100β ToPro
GST-π
S100β
PSA-NCAM
% of immunoreactive cells

Figure S7. hiPS-NSC trasplantation in neonatal MLD mice. (**A**) Dot plots showing the percentage of engrafted cells, the extent of rostro-caudal cell distribution and the percentage of engrafted cells (hNuclei) expressing the proliferation marker Ki67 (Ki67+hNu+) in MLD immunodeficient mice transplanted at PND60 or PND1 with ND hiPS-NSCs (grey circles), ARSA-MLD hiPS-NSCs (green circles) or hfNSCs (grey triangles), and analyzed at 3 months (black border) and 6 months (red border) after treatment. Data are expressed as the mean±SEM; n=3-9. Each dot represents a transplanted animal. *$p<0.05$, **$p<0.01$, Oneway ANOVA followed by Bonferroni post-test. NS, not significant ($p>0.05$). (**B**) Representative confocal picture of hiPS-NSC (hNuclei) engrafted in the controlateral hemisphere 3 months after transplantation in PND60 mice. Nuclei counterstained with dapi; scale bar: 50µm. (**C**) Schematic representation of the bilateral intraventricular transplantation in the neonatal murine brain (200.000 cells/injection site). (**D**) Graph showing the distribution of engrafted cells in 5-6 month-old MLD mice transplanted at PND60 (red) or PND1 (blue) assessed in serial coronal slices along the rostro-caudal brain axis, from the olfactory bulb to the cerebellum. Data are expressed as percentage of hNuclei+ cells on the total transplanted cells; mean±SEM; n=9 and n=6 for mice transplanted at PND60 and PND1, respectively. (**E**) Representative composite picture of hiPS-NSCs (hMito, green) in the cerebellum of a MLD mouse transplanted at PND1 and analyzed at 6 months of age. Inset shows higher magnification of engrafted cells. Scale bar: 100µm. (**F**) Representative confocal images and quantification of hiPS-NSC-derived neuronal and glial cells in MLD mice transplanted at PND60 or PND1 and analyzed at 5-6 months of age. Data are expressed as percentage of lineage-positive cells (oligodendrocytes, GST-π; astrocytes, S100-β; and neuronal precursors, PSA-NCAM) on hNuclei-positive cells. Nuclei counterstained with ToPro (blue). Data are the mean±SEM; n=2-7 mice/group. Scale bar: 20µm

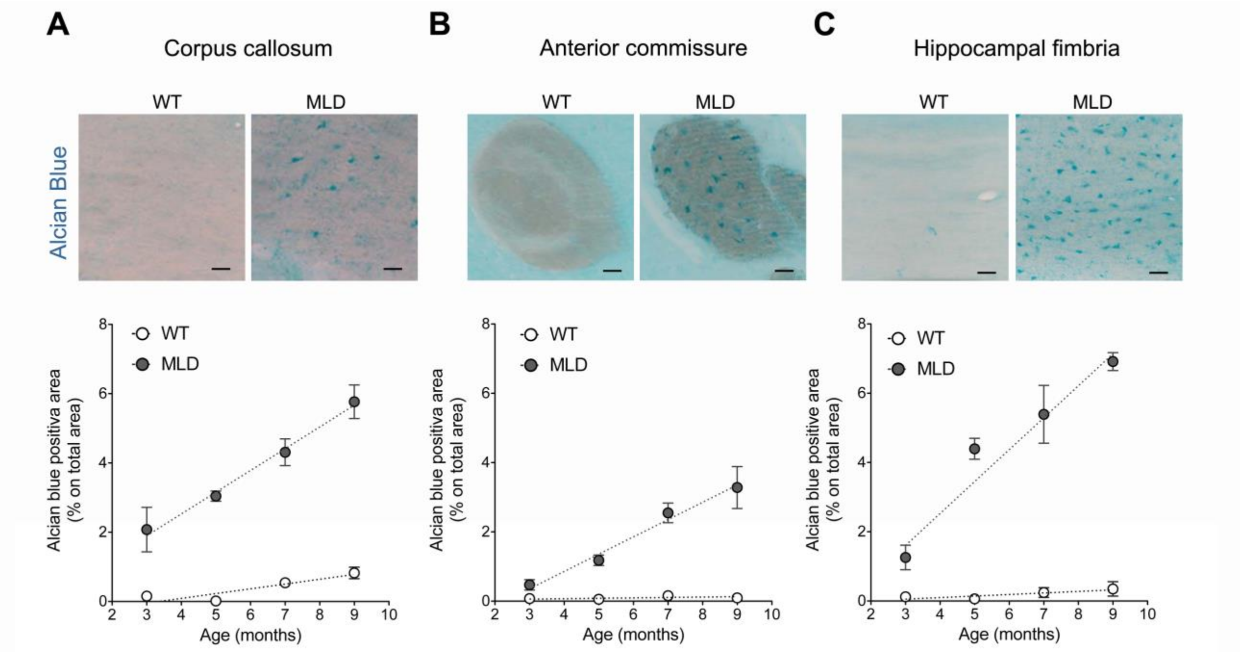

Figure S8. Age-dependent increase of sulfatide storage in immunodeficient MLD mice. Brain tissue sections including the corpus callosum (**A**), anterior commissure **(B**) and hippocampal fimbria (**C**) of *Rag*$^{-/-}$;γ-*chain*$^{-/-}$;*As2*$^{-/-}$ (MLD) and of *Rag*$^{-/-}$;γ-*chain*$^{-/-}$;*As2*$^{+/+}$ (WT) (3, 5, 7 and 9 month-old) were stained with Alcian blue to detect sulfatide accumulation. Representative pictures from 5 month-old mice are shown. Scale bar: 50µm. Time-course quantification of Alcian blue-positive areas revealed an age-dependent sulfatide accumulation in MLD mice. Sulfatide storage was almost undetectable in WT littermates at any age considered. Data are the mean±SEM. n=2 mice/group; 4 images/section; 2 sections/mouse.

References

1. Freberg CT, Dahl JA, Timoskainen S et al. Epigenetic reprogramming of OCT4 and NANOG regulatory regions by embryonal carcinoma cell extract. Mol Biol Cell. 2007;18:1543-1553.

Table S1. Summary of hiPSC clones obtained after reprogramming of human fibroblasts from normal donors (ND). Normal donor-derived adult (ND1) and neonatal (ND2) skin fibroblasts were transduced at MOI=1 or MOI=3 using a monocistronic Cre-excisable lentiviral vector (LV) in which the expression of the master reprograming factors OCT4, SOX2 and KLF4 is driven by the human SFFV promoter (LV.OSK). Reprogramming efficiency was calculated as the percentage of primary hiPSC colonies on the number of plated fibroblasts. The copies of integrated LV genome/host genome (vector copy number, VCN) are expressed as the mean ± SEM, n=3 experiments in triplicates. hiPSC clones highlighted in grey were discarded due to the presence of abnormal karyotype (ND1.2) or excessively high VCN (ND2.1).

Normal donors (fibroblasts)	LV.OSK MOI	Reprogramming efficiency	hiPSC clones	LV.OSK VCN
ND1	1	0.009%	ND1.1	1.9 ± 0.4
			ND1.2	0.8 ± 0.5
			ND1.3	1.6 ± 0.1
ND2	3	0.030%	ND2.1	30.2 ± 1.3
			ND2.2	1.7 ± 0.1
			ND2.3	3.8 ± 0.4

Supplemental table 2: List of genes up-regulated (red) and down-regulated (green) in hiPSCs with respect to donor fibroblasts (Absolute fold change > 1,5; False discovery rate < 0,05)

Gene symbol	Gene Name	Fold change	False discovery rate
CD24	CD24 molecule	6,84	1,27E-04
LRRN1	leucine rich repeat neuronal 1	6,63	1,35E-04
DSG2	desmoglein 2	5,94	1,52E-05
EPCAM	epithelial cell adhesion molecule	5,86	2,52E-03
LIN28A	lin-28 homolog A (C. elegans)	5,80	6,21E-05
TDGF1	teratocarcinoma-derived growth factor 1	5,78	2,68E-02
SPP1	secreted phosphoprotein 1	5,71	3,16E-04
CXADR	coxsackie virus and adenovirus receptor	5,67	3,92E-05
SEMA6A	sema domain, transmembrane domain (TM), and cytoplasmic domain, (semaphorin) 6A	5,64	1,15E-03
CLDN6	claudin 6	5,55	1,94E-04
DPPA4	developmental pluripotency associated 4	5,50	2,38E-04
TM4SF18	transmembrane 4 L six family member 18	5,45	2,29E-03
CRABP1	cellular retinoic acid binding protein 1	5,25	7,35E-04
DSC2	desmocollin 2	5,14	9,82E-06
EDNRB	endothelin receptor type B	5,09	3,27E-03
LIN28B	lin-28 homolog B (C. elegans)	5,07	1,38E-05
PTPRZ1	protein tyrosine phosphatase, receptor-type, Z polypeptide 1	5,04	9,16E-03
SULT1C4	sulfotransferase family, cytosolic, 1C, member 4	5,03	2,63E-03
CST1	cystatin SN	4,97	1,19E-03
KAL1	Kallmann syndrome 1 sequence	4,93	7,12E-03
SOX2	SRY (sex determining region Y)-box 2	4,89	9,76E-04
IGF2BP1	insulin-like growth factor 2 mRNA binding protein 1	4,80	3,61E-05
FZD3	frizzled homolog 3 (Drosophila)	4,79	2,33E-06
RGS5	regulator of G-protein signaling 5	4,78	5,74E-03
LIN28A	lin-28 homolog A (C. elegans)	4,70	6,15E-04
CNTNAP3	contactin associated protein-like 3	4,70	4,33E-04
B3GALT1	UDP-Gal:betaGlcNAc beta 1,3-galactosyltransferase, polypeptide 1	4,58	4,13E-03
NANOG	Nanog homeobox	4,56	2,77E-02
ZIC3	Zic family member 3 (odd-paired homolog, Drosophila)	4,53	1,17E-02
EPCAM	epithelial cell adhesion molecule	4,51	5,04E-03
CT45A6	cancer/testis antigen family 45, member A6	4,48	1,69E-02
KRT8	keratin 8	4,47	6,28E-06
PROM1	prominin 1	4,45	1,00E-04
PRTG	protogenin	4,37	4,83E-03
IQGAP2	IQ motif containing GTPase activating protein 2	4,34	4,17E-03
MAL2	mal, T-cell differentiation protein 2	4,34	2,31E-02
GPC4	glypican 4	4,32	1,86E-03
CCDC88C	coiled-coil domain containing 88C	4,29	2,38E-05
F2RL1	coagulation factor II (thrombin) receptor-like 1	4,25	2,28E-03
NANOG	Nanog homeobox	4,21	2,22E-02
PODXL	podocalyxin-like	4,21	1,72E-03
KLHL4	kelch-like 4 (Drosophila)	4,17	9,12E-04
CHD7	chromodomain helicase DNA binding protein 7	4,14	2,39E-06
PAR5	Prader-Willi/Angelman syndrome-5	4,14	6,49E-04
CD200	CD200 molecule	4,11	8,78E-04
POU5F1	POU class 5 homeobox 1	4,09	8,04E-03
SLAIN1	SLAIN motif family, member 1	4,08	1,00E-04
SYT1	synaptotagmin I	4,07	8,15E-04
KIF5C	kinesin family member 5C	4,02	4,49E-04
SLC7A3	solute carrier family 7 (cationic amino acid transporter, y+ system), member 3	3,98	9,48E-03
KDR	kinase insert domain receptor (a type III receptor tyrosine kinase)	3,90	1,00E-04
GRID2	glutamate receptor, ionotropic, delta 2	3,88	3,29E-02
USP44	ubiquitin specific peptidase 44	3,87	2,14E-02
EPHA7	EPH receptor A7	3,87	1,18E-03
FAM60A	family with sequence similarity 60, member A	3,86	7,10E-05
CDH1	cadherin 1, type 1, E-cadherin (epithelial)	3,79	2,75E-02
ELMO1	engulfment and cell motility 1	3,79	2,12E-04
FAM169A	family with sequence similarity 169, member A	3,79	1,10E-03
CRISPLD1	cysteine-rich secretory protein LCCL domain containing 1	3,79	1,63E-04
CYP2S1	cytochrome P450, family 2, subfamily S, polypeptide 1	3,76	2,02E-03
SNORD116-21	small nucleolar RNA, C/D box 116-21	3,75	1,74E-03
B4GALT6	UDP-Gal:betaGlcNAc beta 1,4- galactosyltransferase, polypeptide 6	3,75	3,61E-04
NRK	Nik related kinase	3,70	1,18E-02
SALL4	sal-like 4 (Drosophila)	3,70	1,62E-03
FABP5	fatty acid binding protein 5 (psoriasis-associated)	3,69	8,31E-03
NR6A1	nuclear receptor subfamily 6, group A, member 1	3,67	2,13E-03
RBPMS2	RNA binding protein with multiple splicing 2	3,66	1,74E-03
CENPV	centromere protein V	3,64	5,01E-04
VAMP8	vesicle-associated membrane protein 8 (endobrevin)	3,63	5,78E-05
RNASE1	ribonuclease, RNase A family, 1 (pancreatic)	3,58	4,29E-02
FAM71F1	family with sequence similarity 71, member F1	3,57	1,32E-02
NTS	neurotensin	3,57	2,30E-02
SLC35F1	solute carrier family 35, member F1	3,55	9,12E-04
TNFRSF21	tumor necrosis factor receptor superfamily, member 21	3,54	1,46E-03
DNMT3B	DNA (cytosine-5-)-methyltransferase 3 beta	3,52	1,61E-02
LPAR4	lysophosphatidic acid receptor 4	3,51	1,54E-03
LPHN3	latrophilin 3	3,48	5,26E-03
PKP2	plakophilin 2	3,48	3,71E-04
GABRB3	gamma-aminobutyric acid (GABA) A receptor, beta 3	3,47	2,04E-02
IL1A	interleukin 1, alpha	3,45	3,69E-02
FABP5	fatty acid binding protein 5 (psoriasis-associated)	3,44	2,98E-03
HEY2	hairy/enhancer-of-split related with YRPW motif 2	3,43	2,85E-02
CNTNAP3	contactin associated protein-like 3	3,42	4,90E-04
KIF1A	kinesin family member 1A	3,41	5,01E-03
ACTC1	actin, alpha, cardiac muscle 1	3,37	2,95E-02
MYEF2	myelin expression factor 2	3,35	3,71E-04
VAT1L	vesicle amine transport protein 1 homolog (T. californica)-like	3,35	6,32E-03
PLP1	proteolipid protein 1	3,34	1,85E-03
PCDH11Y	protocadherin 11 Y-linked	3,33	4,96E-02
KRT18	keratin 18	3,33	1,47E-03
FBXO16	F-box protein 16	3,30	6,38E-04
CDH2	cadherin 2, type 1, N-cadherin (neuronal)	3,28	1,20E-02
CALB1	calbindin 1, 28kDa	3,28	2,80E-02
FREM2	FRAS1 related extracellular matrix protein 2	3,27	3,08E-02
LYN	v-yes-1 Yamaguchi sarcoma viral related oncogene homolog	3,26	1,02E-02
GAL	galanin prepropeptide	3,25	3,81E-03
CDH3	cadherin 3, type 1, P-cadherin (placental)	3,25	1,95E-02
ARID3B	AT rich interactive domain 3B (BRIGHT-like)	3,25	1,77E-04
NFE2L3	nuclear factor (erythroid-derived 2)-like 3	3,21	2,50E-04
CECR2	cat eye syndrome chromosome region, candidate 2	3,20	8,22E-03
SNORD109A	small nucleolar RNA, C/D box 109A	3,20	2,64E-03
SLCO4C1	solute carrier organic anion transporter family, member 4C1	3,19	4,16E-02
MST4	serine/threonine protein kinase MST4	3,17	5,22E-04
POU5F1B	POU class 5 homeobox 1B	3,17	3,83E-02
F11R	F11 receptor	3,15	1,79E-03
ZNF114	zinc finger protein 114	3,15	1,20E-02
SERPINB9	serpin peptidase inhibitor, clade B (ovalbumin), member 9	3,15	2,83E-02
RND2	Rho family GTPase 2	3,15	1,35E-03
TRIML2	tripartite motif family-like 2	3,14	6,78E-03
ALX1	ALX homeobox 1	3,12	6,30E-04
SNORD116-13	small nucleolar RNA, C/D box 116-13	3,11	7,70E-03
ARSE	arylsulfatase E (chondrodysplasia punctata 1)	3,11	9,30E-03
BEX1	brain expressed, X-linked 1	3,10	8,63E-04
SFRP2	secreted frizzled-related protein 2	3,10	2,98E-02
SNORD25	small nucleolar RNA, C/D box 25	3,09	3,13E-03
CHRNA5	cholinergic receptor, nicotinic, alpha 5	3,09	5,79E-04
ALPL	alkaline phosphatase, liver/bone/kidney	3,08	2,11E-02
GYG2	glycogenin 2	3,08	3,50E-03
TUBB2B	tubulin, beta 2B	3,06	1,30E-03
ZNF483	zinc finger protein 483	3,05	1,34E-02
OCLN	occludin	3,03	2,92E-02
GATM	glycine amidinotransferase (L-arginine:glycine amidinotransferase)	3,03	4,24E-03
ST6GAL1	ST6 beta-galactosamide alpha-2,6-sialyltranferase 1	3,03	1,72E-02
ANXA3	annexin A3	3,02	5,38E-03
EPB41L4A	erythrocyte membrane protein band 4.1 like 4A	3,02	3,69E-03
CYTSB	cytospin B	2,98	5,57E-05
SYTL5	synaptotagmin-like 5	2,98	8,12E-03
CRMP1	collapsin response mediator protein 1	2,96	2,30E-05
KIAA1324L	KIAA1324-like	2,96	5,01E-03
ETV4	ets variant 4	2,96	2,47E-02
C7orf46	chromosome 7 open reading frame 46	2,95	6,74E-04

FRMD4B	FERM domain containing 4B	2,95	1,38E-02
ZNF711	zinc finger protein 711	2,94	1,20E-04
JUP	junction plakoglobin	2,94	2,32E-04
PCDH10	protocadherin 10	2,93	8,60E-03
TRIM24	tripartite motif-containing 24	2,92	3,63E-05
ZNF204P	zinc finger protein 204, pseudogene	2,89	4,64E-02
CTSL2	cathepsin L2	2,89	7,60E-03
SLC35F2	solute carrier family 35, member F2	2,85	5,68E-03
E2F5	E2F transcription factor 5, p130-binding	2,85	1,92E-05
SNORD116-22	small nucleolar RNA, C/D box 116-22	2,84	1,74E-03
PLS1	plastin 1	2,83	1,57E-03
OCIAD2	OCIA domain containing 2	2,83	4,55E-03
PDK3	pyruvate dehydrogenase kinase, isozyme 3	2,82	1,23E-02
C10orf58	chromosome 10 open reading frame 58	2,82	7,87E-05
GNA14	guanine nucleotide binding protein (G protein), alpha 14	2,82	1,63E-02
PTPRD	protein tyrosine phosphatase, receptor type, D	2,81	2,26E-03
psiTPTE22	TPTE pseudogene	2,81	4,26E-02
ITGA6	integrin, alpha 6	2,80	3,98E-02
ST6GALNAC3	ST6 (alpha-N-acetyl-neuraminyl-2,3-beta-galactosyl-1,3)-N-acetylgalactosaminide alpha-2,6-sialyltransferase 3	2,80	6,54E-03
RNFT2	ring finger protein, transmembrane 2	2,79	1,21E-05
ELMO1	engulfment and cell motility 1	2,78	3,64E-04
VANGL2	vang-like 2 (van gogh, Drosophila)	2,78	1,42E-05
PLA2G16	phospholipase A2, group XVI	2,77	4,47E-03
RASGRF2	Ras protein-specific guanine nucleotide-releasing factor 2	2,77	2,70E-02
MLLT11	myeloid/lymphoid or mixed-lineage leukemia (trithorax homolog, Drosophila); translocated to, 11	2,77	2,38E-04
ADD2	adducin 2 (beta)	2,77	2,54E-02
IGFBP2	insulin-like growth factor binding protein 2, 36kDa	2,76	2,34E-03
KRT18	keratin 18	2,75	5,91E-04
MYCN	v-myc myelocytomatosis viral related oncogene, neuroblastoma derived (avian)	2,74	7,66E-03
LEPREL1	leprecan-like 1	2,73	5,85E-03
SALL2	sal-like 2 (Drosophila)	2,73	5,92E-03
GCA	grancalcin, EF-hand calcium binding protein	2,71	9,86E-04
POLR3G	polymerase (RNA) III (DNA directed) polypeptide G (32kD)	2,70	2,21E-02
ARRB1	arrestin, beta 1	2,69	7,50E-03
FIGN	fidgetin	2,69	7,67E-03
CRHBP	corticotropin releasing hormone binding protein	2,69	4,04E-02
L1CAM	L1 cell adhesion molecule	2,69	1,15E-02
NETO2	neuropilin (NRP) and tolloid (TLL)-like 2	2,67	2,74E-02
SCARNA9L	small Cajal body-specific RNA 9-like (retrotransposed)	2,67	8,92E-03
FAM83B	family with sequence similarity 83, member B	2,67	1,23E-02
OVOS	ovostatin	2,67	2,77E-03
FIGNL2	fidgetin-like 2	2,65	1,09E-03
VSNL1	visinin-like 1	2,62	4,37E-02
JARID2	jumonji, AT rich interactive domain 2	2,62	8,18E-03
BUB1B	budding uninhibited by benzimidazoles 1 homolog beta (yeast)	2,62	1,34E-02
GPRC5B	G protein-coupled receptor, family C, group 5, member B	2,62	2,62E-02
SNRPN	small nuclear ribonucleoprotein polypeptide N	2,62	9,28E-03
RNF125	ring finger protein 125	2,62	4,33E-02
KRT18	keratin 18	2,61	1,30E-03
PLCH1	phospholipase C, eta 1	2,61	4,02E-04
LIPG	lipase, endothelial	2,60	2,58E-03
GPM6B	glycoprotein M6B	2,60	1,34E-02
WNK3	WNK lysine deficient protein kinase 3	2,59	5,69E-03
GPR50	G protein-coupled receptor 50	2,59	2,71E-03
SLC7A2	solute carrier family 7 (cationic amino acid transporter, y+ system), member 2	2,59	4,02E-04
SILV	silver homolog (mouse)	2,59	2,41E-02
PIM2	pim-2 oncogene	2,58	3,99E-02
SORBS1	sorbin and SH3 domain containing 1	2,58	1,18E-04
TSPAN7	tetraspanin 7	2,58	1,95E-03
ATF7IP2	activating transcription factor 7 interacting protein 2	2,58	4,33E-03
DEPDC1B	DEP domain containing 1B	2,56	4,13E-03
PIPOX	pipecolic acid oxidase	2,53	2,80E-02
DTNA	dystrobrevin, alpha	2,53	1,27E-02
KCTD14	potassium channel tetramerisation domain containing 14	2,52	1,93E-03
MTF2	metal response element binding transcription factor 2	2,52	2,23E-04
TNIK	TRAF2 and NCK interacting kinase	2,52	1,76E-02
NSUN7	NOP2/Sun domain family, member 7	2,51	4,84E-03
SORL1	sortilin-related receptor, L(DLR class) A repeats-containing	2,51	2,18E-02
INA	internexin neuronal intermediate filament protein, alpha	2,51	1,02E-02
SNORD26	small nucleolar RNA, C/D box 26	2,51	1,06E-03
EMB	embigin	2,50	1,49E-03
RASL11B	RAS-like, family 11, member B	2,49	3,50E-02
ST6GAL2	ST6 beta-galactosamide alpha-2,6-sialyltranferase 2	2,49	1,55E-02
NUP210	nucleoporin 210kDa	2,49	3,31E-03
XRCC2	X-ray repair complementing defective repair in Chinese hamster cells 2	2,47	3,02E-03
MCTP1	multiple C2 domains, transmembrane 1	2,47	7,73E-03
FLVCR1	feline leukemia virus subgroup C cellular receptor 1	2,47	2,11E-02
DCDC2	doublecortin domain containing 2	2,46	2,41E-02
NKAIN1	Na+/K+ transporting ATPase interacting 1	2,46	9,18E-03
HELLS	helicase, lymphoid-specific	2,45	1,04E-02
GCNT2	glucosaminyl (N-acetyl) transferase 2, I-branching enzyme (I blood group)	2,44	1,82E-02
GULP1	GULP, engulfment adaptor PTB domain containing 1	2,44	1,65E-03
SNORD30	small nucleolar RNA, C/D box 30	2,44	7,06E-03
KIF21A	kinesin family member 21A	2,44	1,82E-03
ATP1B1	ATPase, Na+/K+ transporting, beta 1 polypeptide	2,44	3,19E-03
MMD	monocyte to macrophage differentiation-associated	2,43	4,10E-03
CYFIP2	cytoplasmic FMR1 interacting protein 2	2,43	2,38E-04
DOCK4	dedicator of cytokinesis 4	2,42	1,75E-02
IGF2BP3	insulin-like growth factor 2 mRNA binding protein 3	2,42	3,26E-03
TRIM71	tripartite motif-containing 71	2,42	2,48E-02
JPH1	junctophilin 1	2,42	1,75E-02
HOOK1	hook homolog 1 (Drosophila)	2,41	2,25E-02
SEPT3	septin 3	2,40	2,49E-02
NES	nestin	2,40	2,55E-03
CARD11	caspase recruitment domain family, member 11	2,40	1,04E-03
B3GNT5	UDP-GlcNAc:betaGal beta-1,3-N-acetylglucosaminyltransferase 5	2,40	1,43E-02
ZNF724P	zinc finger protein 724, pseudogene	2,39	6,70E-03
VASH2	vasohibin 2	2,38	3,36E-02
PEG10	paternally expressed 10	2,38	2,67E-02
ISYNA1	inositol-3-phosphate synthase 1	2,38	2,81E-03
BLM	Bloom syndrome, RecQ helicase-like	2,37	4,10E-03
NAP1L3	nucleosome assembly protein 1-like 3	2,37	1,39E-02
GPR63	G protein-coupled receptor 63	2,37	8,99E-03
HRASLS5	HRAS-like suppressor family, member 5	2,37	2,80E-02
ZNF462	zinc finger protein 462	2,36	2,97E-04
CNTNAP3	contactin associated protein-like 3	2,36	1,36E-02
ITM2A	integral membrane protein 2A	2,35	3,11E-02
CPVL	carboxypeptidase, vitellogenic-like	2,35	4,29E-04
STC1	stanniocalcin 1	2,35	4,38E-02
MRS2	MRS2 magnesium homeostasis factor homolog (S. cerevisiae)	2,35	7,91E-05
MAPK10	mitogen-activated protein kinase 10	2,35	1,36E-02
PRKAR2B	protein kinase, cAMP-dependent, regulatory, type II, beta	2,35	3,00E-02
FAT3	FAT tumor suppressor homolog 3 (Drosophila)	2,34	5,79E-04
PCDHA12	protocadherin alpha 12	2,34	1,69E-03
MEGF10	multiple EGF-like-domains 10	2,34	1,89E-03
SYT14	synaptotagmin XIV	2,34	2,60E-02
BCL11A	B-cell CLL/lymphoma 11A (zinc finger protein)	2,34	1,74E-02
CCNJL	cyclin J-like	2,33	6,84E-04
TSPAN12	tetraspanin 12	2,33	2,19E-04
BUB1	budding uninhibited by benzimidazoles 1 homolog (yeast)	2,33	2,45E-02
FNBP1L	formin binding protein 1-like	2,33	6,69E-04
ZMAT1	zinc finger, matrin type 1	2,32	3,19E-02
IGSF3	immunoglobulin superfamily, member 3	2,32	5,22E-04
CAPN6	calpain 6	2,32	2,11E-02
DYSF	dysferlin, limb girdle muscular dystrophy 2B (autosomal recessive)	2,32	3,67E-02
SOX5	SRY (sex determining region Y)-box 5	2,31	1,68E-02
SNORD28	small nucleolar RNA, C/D box 28	2,31	1,30E-03
ADRBK2	adrenergic, beta, receptor kinase 2	2,30	6,95E-03
C6orf168	chromosome 6 open reading frame 168	2,30	7,66E-03
RAB15	RAB15, member RAS onocogene family	2,29	1,31E-03
PCYT1B	phosphate cytidylyltransferase 1, choline, beta	2,29	1,50E-02
GPR37	G protein-coupled receptor 37 (endothelin receptor type B-like)	2,29	2,50E-04
TTK	TTK protein kinase	2,29	3,57E-02
NEDD4L	neural precursor cell expressed, developmentally down-regulated 4-like	2,28	5,04E-03

C15orf42	chromosome 15 open reading frame 42	2,28	1,05E-02
UNC5D	unc-5 homolog D (C. elegans)	2,28	1,20E-02
ZIC2	Zic family member 2 (odd-paired homolog, Drosophila)	2,28	1,54E-02
LRRC8B	leucine rich repeat containing 8 family, member B	2,26	1,23E-02
PLK1	polo-like kinase 1	2,26	1,64E-02
SPINT2	serine peptidase inhibitor, Kunitz type, 2	2,26	9,74E-03
SNORD27	small nucleolar RNA, C/D box 27	2,25	2,89E-03
RPS6KA1	ribosomal protein S6 kinase, 90kDa, polypeptide 1	2,25	1,72E-03
MUM1L1	melanoma associated antigen (mutated) 1-like 1	2,25	2,69E-02
TMEM88	transmembrane protein 88	2,25	1,26E-02
HIST1H2BH	histone cluster 1, H2bh	2,24	2,09E-02
TMSB15B	thymosin beta 15B	2,23	2,04E-03
SLC25A13	solute carrier family 25, member 13 (citrin)	2,23	8,12E-04
SNORD22	small nucleolar RNA, C/D box 22	2,22	1,27E-02
SNORD116-11	small nucleolar RNA, C/D box 116-11	2,22	6,71E-03
FXYD6	FXYD domain containing ion transport regulator 6	2,21	1,95E-03
MARK1	MAP/microtubule affinity-regulating kinase 1	2,21	2,02E-03
FANCD2	Fanconi anemia, complementation group D2	2,20	1,17E-02
CTPS2	CTP synthase II	2,20	1,19E-03
UCP2	uncoupling protein 2 (mitochondrial, proton carrier)	2,19	5,82E-03
MSI2	musashi homolog 2 (Drosophila)	2,19	1,20E-02
VAV3	vav 3 guanine nucleotide exchange factor	2,19	1,61E-03
HIST1H3F	histone cluster 1, H3f	2,19	1,64E-02
NHSL1	NHS-like 1	2,19	1,88E-02
DLGAP5	discs, large (Drosophila) homolog-associated protein 5	2,19	4,75E-02
GNAL	guanine nucleotide binding protein (G protein), alpha activating activity polypeptide, olfactory type	2,19	1,49E-04
CCND2	cyclin D2	2,19	2,32E-02
ADAMTS9	ADAM metallopeptidase with thrombospondin type 1 motif, 9	2,19	4,06E-02
CDCA7	cell division cycle associated 7	2,18	1,38E-02
RRAGD	Ras-related GTP binding D	2,18	2,76E-02
TSPAN15	tetraspanin 15	2,18	5,22E-04
HIST1H2BF	histone cluster 1, H2bf	2,17	2,98E-02
FAM72D	family with sequence similarity 72, member D	2,17	3,80E-02
SGOL1	shugoshin-like 1 (S. pombe)	2,17	1,56E-02
PELI1	pellino homolog 1 (Drosophila)	2,16	1,52E-02
ORC1L	origin recognition complex, subunit 1-like (S. cerevisiae)	2,16	4,45E-03
ATAD5	ATPase family, AAA domain containing 5	2,16	8,24E-03
HOXA2	homeobox A2	2,16	2,11E-02
C21orf105	chromosome 21 open reading frame 105	2,16	5,74E-03
SNORD116-8	small nucleolar RNA, C/D box 116-8	2,15	5,05E-03
SH3BGRL2	SH3 domain binding glutamic acid-rich protein like 2	2,15	8,12E-04
HMMR	hyaluronan-mediated motility receptor (RHAMM)	2,14	2,22E-02
STRBP	spermatid perinuclear RNA binding protein	2,14	1,19E-02
SLC16A9	solute carrier family 16, member 9 (monocarboxylic acid transporter 9)	2,14	2,10E-02
MYBL2	v-myb myeloblastosis viral oncogene homolog (avian)-like 2	2,13	9,70E-03
GABRA5	gamma-aminobutyric acid (GABA) A receptor, alpha 5	2,13	2,12E-02
TMSB15A	thymosin beta 15a	2,13	2,20E-02
FAM72D	family with sequence similarity 72, member D	2,12	1,55E-02
FAM49B	family with sequence similarity 49, member B	2,12	1,75E-04
MCAM	melanoma cell adhesion molecule	2,11	2,07E-02
PHC1	polyhomeotic homolog 1 (Drosophila)	2,10	1,60E-02
DNA2	DNA replication helicase 2 homolog (yeast)	2,10	3,43E-03
XCL1	chemokine (C motif) ligand 1	2,10	1,03E-02
PARP8	poly (ADP-ribose) polymerase family, member 8	2,10	1,58E-02
ARHGAP42	Rho GTPase activating protein 42	2,09	2,02E-03
SNORD116-29	small nucleolar RNA, C/D box 116-29	2,09	1,37E-02
SLC44A5	solute carrier family 44, member 5	2,09	1,06E-03
GINS1	GINS complex subunit 1 (Psf1 homolog)	2,09	1,56E-02
FAM72D	family with sequence similarity 72, member D	2,09	1,23E-02
PAFAH1B3	platelet-activating factor acetylhydrolase 1b, catalytic subunit 3 (29kDa)	2,08	2,54E-03
SMS	spermine synthase	2,08	7,28E-03
CCNB2	cyclin B2	2,08	1,57E-02
BTBD11	BTB (POZ) domain containing 11	2,08	3,02E-02
MERTK	c-mer proto-oncogene tyrosine kinase	2,08	2,38E-04
KCNMB4	potassium large conductance calcium-activated channel, subfamily M, beta member 4	2,08	8,26E-03
LRRTM4	leucine rich repeat transmembrane neuronal 4	2,07	3,36E-02
SNX10	sorting nexin 10	2,07	1,78E-03
RCOR2	REST corepressor 2	2,07	1,33E-02
ETNK2	ethanolamine kinase 2	2,07	1,20E-02
PREX2	phosphatidylinositol-3,4,5-trisphosphate-dependent Rac exchange factor 2	2,06	1,29E-02
AP1S2	adaptor-related protein complex 1, sigma 2 subunit	2,06	1,27E-02
CDC20	cell division cycle 20 homolog (S. cerevisiae)	2,06	2,25E-02
NAP1L2	nucleosome assembly protein 1-like 2	2,05	5,79E-04
ARHGAP19	Rho GTPase activating protein 19	2,05	1,38E-02
FGD6	FYVE, RhoGEF and PH domain containing 6	2,04	9,74E-03
ELOVL7	ELOVL family member 7, elongation of long chain fatty acids (yeast)	2,04	4,90E-02
CA14	carbonic anhydrase XIV	2,04	2,10E-02
AFF2	AF4/FMR2 family, member 2	2,04	2,62E-02
PIK3R3	phosphoinositide-3-kinase, regulatory subunit 3 (gamma)	2,03	2,69E-02
MED12L	mediator complex subunit 12-like	2,03	8,01E-03
CCNJ	cyclin J	2,03	1,38E-05
SLC2A1	solute carrier family 2 (facilitated glucose transporter), member 1	2,02	1,23E-03
PAIP2B	poly(A) binding protein interacting protein 2B	2,01	4,33E-02
PNP	purine nucleoside phosphorylase	2,01	8,43E-03
PLEKHH1	pleckstrin homology domain containing, family H (with MyTH4 domain) member 1	2,01	4,17E-03
SLITRK4	SLIT and NTRK-like family, member 4	2,01	9,19E-03
PARM1	prostate androgen-regulated mucin-like protein 1	2,01	4,71E-02
TMEM108	transmembrane protein 108	2,00	4,17E-03
PPAT	phosphoribosyl pyrophosphate amidotransferase	2,00	2,57E-03
MT1F	metallothionein 1F	2,00	1,17E-02
ZNF280C	zinc finger protein 280C	2,00	9,78E-03
LMNB1	lamin B1	1,99	1,74E-02
HERC5	hect domain and RLD 5	1,99	1,39E-02
SPAG5	sperm associated antigen 5	1,99	2,32E-02
RAC3	ras-related C3 botulinum toxin substrate 3 (rho family, small GTP binding protein Rac3)	1,99	1,75E-04
SNORD116-26	small nucleolar RNA, C/D box 116-26	1,99	3,46E-02
CCNB1	cyclin B1	1,99	2,06E-02
SNORD116-28	small nucleolar RNA, C/D box 116-28	1,99	1,23E-02
SNCA	synuclein, alpha (non A4 component of amyloid precursor)	1,99	1,85E-03
MND1	meiotic nuclear divisions 1 homolog (S. cerevisiae)	1,98	3,63E-02
IGSF1	immunoglobulin superfamily, member 1	1,98	2,40E-02
CYP26A1	cytochrome P450, family 26, subfamily A, polypeptide 1	1,98	3,57E-02
SHISA9	shisa homolog 9 (Xenopus laevis)	1,98	5,26E-03
MSI1	musashi homolog 1 (Drosophila)	1,98	1,19E-03
SDK2	sidekick homolog 2 (chicken)	1,97	5,79E-03
PRICKLE1	prickle homolog 1 (Drosophila)	1,97	2,70E-02
KIAA1598	KIAA1598	1,97	5,99E-03
NEFL	neurofilament, light polypeptide	1,96	2,30E-03
HMGA2	high mobility group AT-hook 2	1,96	1,92E-02
GALNT7	UDP-N-acetyl-alpha-D-galactosamine:polypeptide N-acetylgalactosaminyltransferase 7 (GalNAc-T7)	1,96	1,49E-03
PRKCZ	protein kinase C, zeta	1,96	1,27E-02
CNIH2	cornichon homolog 2 (Drosophila)	1,96	9,59E-04
TSPAN33	tetraspanin 33	1,95	3,09E-02
LOC283174	hypothetical LOC283174	1,95	1,95E-02
GPR125	G protein-coupled receptor 125	1,95	3,27E-03
ORC6L	origin recognition complex, subunit 6 like (yeast)	1,95	2,68E-03
TMEM178	transmembrane protein 178	1,95	3,62E-02
FGF11	fibroblast growth factor 11	1,95	2,26E-02
CENPF	centromere protein F, 350/400kDa (mitosin)	1,95	3,10E-02
EPHB1	EPH receptor B1	1,95	1,11E-02
C18orf54	chromosome 18 open reading frame 54	1,94	8,91E-03
ZNF280B	zinc finger protein 280B	1,94	3,56E-04
DTL	denticleless homolog (Drosophila)	1,94	3,22E-02
BRIP1	BRCA1 interacting protein C-terminal helicase 1	1,94	4,47E-02
FAM49A	family with sequence similarity 49, member A	1,93	1,82E-02
AFAP1L2	actin filament associated protein 1-like 2	1,93	2,82E-02
TMEM133	transmembrane protein 133	1,92	2,74E-03
FRMD5	FERM domain containing 5	1,92	1,59E-02
KBTBD8	kelch repeat and BTB (POZ) domain containing 8	1,92	6,81E-03
CADM2	cell adhesion molecule 2	1,91	5,71E-03
SNORD116-27	small nucleolar RNA, C/D box 116-27	1,91	1,70E-02
HTR1D	5-hydroxytryptamine (serotonin) receptor 1D	1,91	6,22E-03
ACP6	acid phosphatase 6, lysophosphatidic	1,90	4,33E-04
HS6ST2	heparan sulfate 6-O-sulfotransferase 2	1,90	1,12E-02

CIB2	calcium and integrin binding family member 2	1,90	1,03E-02
AURKB	aurora kinase B	1,90	9,10E-03
SOX11	SRY (sex determining region Y)-box 11	1,90	2,76E-04
RNF122	ring finger protein 122	1,90	1,23E-03
ANO5	anoctamin 5	1,90	2,58E-02
CDC25A	cell division cycle 25 homolog A (S. pombe)	1,90	8,94E-03
AP1G2	adaptor-related protein complex 1, gamma 2 subunit	1,90	2,16E-05
UPF3B	UPF3 regulator of nonsense transcripts homolog B (yeast)	1,90	3,30E-03
TOX3	TOX high mobility group box family member 3	1,89	1,94E-02
SNORD31	small nucleolar RNA, C/D box 31	1,89	5,31E-03
MAD2L1	MAD2 mitotic arrest deficient-like 1 (yeast)	1,89	1,61E-02
CDK1	cyclin-dependent kinase 1	1,89	3,94E-02
IQCA1	IQ motif containing with AAA domain 1	1,88	1,46E-03
SRSF12	serine/arginine-rich splicing factor 12	1,88	1,97E-03
PARD6B	par-6 partitioning defective 6 homolog beta (C. elegans)	1,88	1,19E-03
RFC3	replication factor C (activator 1) 3, 38kDa	1,87	5,09E-03
KIF14	kinesin family member 14	1,87	2,97E-02
IGLON5	IgLON family member 5	1,87	3,86E-03
GPR125	G protein-coupled receptor 125	1,87	2,97E-04
PDE7A	phosphodiesterase 7A	1,87	4,06E-03
DEPDC1	DEP domain containing 1	1,87	2,07E-02
NASP	nuclear autoantigenic sperm protein (histone-binding)	1,87	3,94E-03
RAVER2	ribonucleoprotein, PTB-binding 2	1,87	5,01E-03
MLLT4	myeloid/lymphoid or mixed-lineage leukemia (trithorax homolog, Drosophila); translocated to, 4	1,87	1,19E-03
C11orf82	chromosome 11 open reading frame 82	1,87	1,43E-02
MYO5C	myosin VC	1,86	1,71E-02
PRIM1	primase, DNA, polypeptide 1 (49kDa)	1,86	2,03E-02
PDE3B	phosphodiesterase 3B, cGMP-inhibited	1,86	1,31E-02
PLK4	polo-like kinase 4	1,86	3,89E-02
AIF1L	allograft inflammatory factor 1-like	1,86	1,87E-02
PPP1R9A	protein phosphatase 1, regulatory (inhibitor) subunit 9A	1,86	3,20E-03
PLA2G7	phospholipase A2, group VII (platelet-activating factor acetylhydrolase, plasma)	1,86	2,18E-02
GNG4	guanine nucleotide binding protein (G protein), gamma 4	1,86	1,63E-02
HIST1H3E	histone cluster 1, H3e	1,86	1,10E-02
LINGO2	leucine rich repeat and Ig domain containing 2	1,86	2,72E-02
PMAIP1	phorbol-12-myristate-13-acetate-induced protein 1	1,85	1,03E-02
LRRC61	leucine rich repeat containing 61	1,85	5,01E-04
CHRNB1	cholinergic receptor, nicotinic, beta 1 (muscle)	1,85	2,31E-03
C4orf21	chromosome 4 open reading frame 21	1,84	5,82E-03
FKBP5	FK506 binding protein 5	1,84	2,85E-02
ZNF608	zinc finger protein 608	1,84	1,64E-02
IRS4	insulin receptor substrate 4	1,84	4,95E-02
TTLL4	tubulin tyrosine ligase-like family, member 4	1,83	2,55E-04
SLFN13	schlafen family member 13	1,83	8,98E-03
FAM84B	family with sequence similarity 84, member B	1,83	3,22E-03
TEX15	testis expressed 15	1,82	1,12E-02
ATRNL1	attractin-like 1	1,82	4,98E-03
C11orf9	chromosome 11 open reading frame 9	1,82	1,56E-02
HSPC159	galectin-related protein	1,82	1,39E-02
ILDR2	immunoglobulin-like domain containing receptor 2	1,82	1,78E-02
SOX15	SRY (sex determining region Y)-box 15	1,82	2,75E-02
ELAVL2	ELAV (embryonic lethal, abnormal vision, Drosophila)-like 2 (Hu antigen B)	1,81	3,24E-03
DCC	deleted in colorectal carcinoma	1,80	1,98E-02
SPRY4	sprouty homolog 4 (Drosophila)	1,80	3,95E-02
SNRPN	small nuclear ribonucleoprotein polypeptide N	1,80	1,57E-02
RARB	retinoic acid receptor, beta	1,79	2,45E-02
MYH10	myosin, heavy chain 10, non-muscle	1,79	1,03E-02
CACHD1	cache domain containing 1	1,79	1,53E-02
RAP1GAP2	RAP1 GTPase activating protein 2	1,79	2,14E-02
KIAA0040	KIAA0040	1,79	4,05E-02
GINS2	GINS complex subunit 2 (Psf2 homolog)	1,79	3,62E-02
GPR143	G protein-coupled receptor 143	1,79	3,26E-02
PGM2L1	phosphoglucomutase 2-like 1	1,79	1,33E-02
OIP5	Opa interacting protein 5	1,79	9,08E-03
C3orf52	chromosome 3 open reading frame 52	1,79	7,78E-03
ALPK3	alpha-kinase 3	1,78	5,59E-03
TDRKH	tudor and KH domain containing	1,78	1,59E-02
NEFM	neurofilament, medium polypeptide	1,78	3,48E-02
ATCAY	ataxia, cerebellar, Cayman type	1,78	2,37E-02
HIST1H3J	histone cluster 1, H3j	1,78	1,76E-02
PRKCH	protein kinase C, eta	1,77	4,58E-03
HN1	hematological and neurological expressed 1	1,77	4,68E-03
TPD52	tumor protein D52	1,77	4,26E-02
CADM4	cell adhesion molecule 4	1,77	1,49E-03
C6orf115	chromosome 6 open reading frame 115	1,77	1,90E-02
RASGEF1A	RasGEF domain family, member 1A	1,77	4,59E-02
RAD51AP1	RAD51 associated protein 1	1,77	3,29E-02
NCAPH	non-SMC condensin I complex, subunit H	1,76	4,59E-02
FLJ36840	hypothetical LOC645524	1,76	7,70E-03
ZMYND8	zinc finger, MYND-type containing 8	1,76	2,66E-04
DDX26B	DEAD/H (Asp-Glu-Ala-Asp/His) box polypeptide 26B	1,76	1,73E-03
GRIK5	glutamate receptor, ionotropic, kainate 5	1,76	2,58E-02
APOO	apolipoprotein O	1,76	5,06E-03
PRR11	proline rich 11	1,76	2,11E-02
GALNT13	UDP-N-acetyl-alpha-D-galactosamine:polypeptide N-acetylgalactosaminyltransferase 13 (GalNAc-T13)	1,76	1,69E-02
CPXM1	carboxypeptidase X (M14 family), member 1	1,75	3,95E-02
CTTNBP2	cortactin binding protein 2	1,75	1,98E-02
RHOF	ras homolog gene family, member F (in filopodia)	1,75	7,43E-03
ARG2	arginase, type II	1,75	2,55E-03
RAD54B	RAD54 homolog B (S. cerevisiae)	1,75	8,13E-04
ZNF92	zinc finger protein 92	1,74	7,36E-03
WBSCR17	Williams-Beuren syndrome chromosome region 17	1,73	3,35E-02
N4BP2	NEDD4 binding protein 2	1,73	1,84E-03
STIL	SCL/TAL1 interrupting locus	1,73	4,75E-03
KIF4A	kinesin family member 4A	1,73	2,77E-02
HMGB3	high-mobility group box 3	1,72	9,51E-03
FGF12	fibroblast growth factor 12	1,72	7,82E-03
KIF18A	kinesin family member 18A	1,72	2,23E-02
RAB3IP	RAB3A interacting protein (rabin3)	1,72	1,52E-02
SNORD116-6	small nucleolar RNA, C/D box 116-6	1,72	3,04E-02
SFMBT2	Scm-like with four mbt domains 2	1,71	1,84E-03
PLCB2	phospholipase C, beta 2	1,71	3,34E-02
NUAK2	NUAK family, SNF1-like kinase, 2	1,71	6,22E-03
MUC3B	mucin 3B, cell surface associated	1,71	4,83E-02
TMEFF1	transmembrane protein with EGF-like and two follistatin-like domains 1	1,71	2,54E-02
CDC25C	cell division cycle 25 homolog C (S. pombe)	1,70	1,10E-02
HMGN5	high-mobility group nucleosome binding domain 5	1,70	1,09E-02
EGLN3	egl nine homolog 3 (C. elegans)	1,70	3,32E-02
PLCXD1	phosphatidylinositol-specific phospholipase C, X domain containing 1	1,69	3,15E-03
CD74	CD74 molecule, major histocompatibility complex, class II invariant chain	1,68	1,02E-02
GFPT2	glutamine-fructose-6-phosphate transaminase 2	1,68	3,26E-02
NANOGP1	Nanog homeobox pseudogene 1	1,68	3,53E-02
ACVR2B	activin A receptor, type IIB	1,68	8,05E-03
BCL2L11	BCL2-like 11 (apoptosis facilitator)	1,68	3,27E-03
MSH2	mutS homolog 2, colon cancer, nonpolyposis type 1 (E. coli)	1,67	1,17E-02
INPP5D	inositol polyphosphate-5-phosphatase, 145kDa	1,67	1,48E-02
GPR98	G protein-coupled receptor 98	1,67	4,84E-02
CDCA2	cell division cycle associated 2	1,67	4,90E-02
POU2F1	POU class 2 homeobox 1	1,67	5,62E-03
LRRN4	leucine rich repeat neuronal 4	1,67	4,18E-02
DDR1	discoidin domain receptor tyrosine kinase 1	1,67	5,79E-04
RBP1	retinol binding protein 1, cellular	1,67	1,92E-03
FAM60A	family with sequence similarity 60, member A	1,67	4,86E-02
LSR	lipolysis stimulated lipoprotein receptor	1,67	4,31E-02
LPHN2	latrophilin 2	1,66	4,06E-03
USP54	ubiquitin specific peptidase 54	1,66	5,01E-04
CDC7	cell division cycle 7 homolog (S. cerevisiae)	1,66	1,82E-02
PFKFB4	6-phosphofructo-2-kinase/fructose-2,6-biphosphatase 4	1,66	1,93E-03
VSIG1	V-set and immunoglobulin domain containing 1	1,66	3,22E-02
PLBD1	phospholipase B domain containing 1	1,65	2,11E-02
SLC6A15	solute carrier family 6 (neutral amino acid transporter), member 15	1,65	1,89E-02
MGAT4C	mannosyl (alpha-1,3-)-glycoprotein beta-1,4-N-acetylglucosaminyltransferase, isozyme C (putative)	1,65	3,43E-02
AMT	aminomethyltransferase	1,65	2,13E-02
STK33	serine/threonine kinase 33	1,65	1,19E-02

ATP1A2	ATPase, Na+/K+ transporting, alpha 2 polypeptide	1,65	3,24E-02
SHC3	SHC (Src homology 2 domain containing) transforming protein 3	1,65	4,56E-02
POLE2	polymerase (DNA directed), epsilon 2 (p59 subunit)	1,65	3,13E-02
CECR1	cat eye syndrome chromosome region, candidate 1	1,64	5,05E-03
AUTS2	autism susceptibility candidate 2	1,64	1,07E-02
ZNF678	zinc finger protein 678	1,64	2,06E-02
PRKCQ	protein kinase C, theta	1,63	1,12E-02
SCML2	sex comb on midleg-like 2 (Drosophila)	1,63	4,69E-02
RAMP2	receptor (G protein-coupled) activity modifying protein 2	1,63	4,68E-03
DSC3	desmocollin 3	1,63	2,03E-02
GRB14	growth factor receptor-bound protein 14	1,63	2,27E-03
JAKMIP2	janus kinase and microtubule interacting protein 2	1,63	2,63E-02
EZR	ezrin	1,63	2,79E-02
CCNA2	cyclin A2	1,63	4,24E-02
ABLIM1	actin binding LIM protein 1	1,62	6,22E-03
BCL9	B-cell CLL/lymphoma 9	1,62	2,79E-03
CNKSR1	connector enhancer of kinase suppressor of Ras 1	1,62	1,02E-02
GRIP1	glutamate receptor interacting protein 1	1,62	7,10E-05
CLMN	calmin (calponin-like, transmembrane)	1,61	1,53E-02
HIST1H2AI	histone cluster 1, H2ai	1,61	1,51E-02
KIF2C	kinesin family member 2C	1,61	4,63E-02
TDP1	tyrosyl-DNA phosphodiesterase 1	1,60	1,31E-03
PTBP2	polypyrimidine tract binding protein 2	1,60	2,09E-02
HIST1H2AE	histone cluster 1, H2ae	1,60	4,65E-02
SLC5A6	solute carrier family 5 (sodium-dependent vitamin transporter), member 6	1,60	7,93E-03
TUBB4	tubulin, beta 4	1,60	2,31E-02
PRKCB	protein kinase C, beta	1,59	2,29E-02
PLEKHA7	pleckstrin homology domain containing, family A member 7	1,59	3,19E-02
SALL1	sal-like 1 (Drosophila)	1,59	1,92E-02
POU5F1P4	POU class 5 homeobox 1 pseudogene 4	1,59	4,38E-02
FAM69B	family with sequence similarity 69, member B	1,59	1,29E-03
NCAPG	non-SMC condensin I complex, subunit G	1,59	3,64E-02
NET1	neuroepithelial cell transforming 1	1,58	4,34E-02
BMP7	bone morphogenetic protein 7	1,58	7,92E-04
HIST1H2AM	histone cluster 1, H2am	1,58	1,01E-02
SEPHS1	selenophosphate synthetase 1	1,58	1,74E-02
BRWD3	bromodomain and WD repeat domain containing 3	1,58	2,75E-02
LOC10028841	similar to Uncharacterized protein LP9056	1,57	2,32E-02
VRK1	vaccinia related kinase 1	1,57	8,43E-03
TRIB1	tribbles homolog 1 (Drosophila)	1,57	4,85E-02
C1orf106	chromosome 1 open reading frame 106	1,57	1,97E-03
SLC5A12	solute carrier family 5 (sodium/glucose cotransporter), member 12	1,57	4,36E-02
TAGLN3	transgelin 3	1,57	4,16E-02
CENPW	centromere protein W	1,57	5,68E-03
PITPNC1	phosphatidylinositol transfer protein, cytoplasmic 1	1,56	2,11E-02
HIST1H2AJ	histone cluster 1, H2aj	1,56	4,02E-02
TLE1	transducin-like enhancer of split 1 (E(sp1) homolog, Drosophila)	1,56	8,12E-04
PAK3	p21 protein (Cdc42/Rac)-activated kinase 3	1,56	3,15E-03
CNKSR2	connector enhancer of kinase suppressor of Ras 2	1,56	1,74E-02
CCDC138	coiled-coil domain containing 138	1,56	7,52E-03
SHROOM2	shroom family member 2	1,56	1,49E-02
MCM3	minichromosome maintenance complex component 3	1,56	1,22E-02
MPPED2	metallophosphoesterase domain containing 2	1,56	2,29E-02
ERC2	ELKS/RAB6-interacting/CAST family member 2	1,56	5,65E-04
LOC80154	hypothetical LOC80154	1,56	1,53E-02
H2AFY2	H2A histone family, member Y2	1,55	1,74E-02
CENPH	centromere protein H	1,55	2,10E-02
MDK	midkine (neurite growth-promoting factor 2)	1,55	4,25E-04
GINS4	GINS complex subunit 4 (Sld5 homolog)	1,55	2,57E-02
SEMA4D	sema domain, immunoglobulin domain (Ig), transmembrane domain (TM) and short cytoplasmic domain, (semaphorin) 4D	1,55	1,52E-02
PARP1	poly (ADP-ribose) polymerase 1	1,54	1,42E-02
MBTD1	mbt domain containing 1	1,54	5,01E-03
CKS1B	CDC28 protein kinase regulatory subunit 1B	1,54	1,12E-02
CXorf57	chromosome X open reading frame 57	1,54	1,55E-02
KIF20B	kinesin family member 20B	1,53	3,85E-02
UGT8	UDP glycosyltransferase 8	1,53	4,25E-02
LBR	lamin B receptor	1,53	3,83E-03
HIST2H4A	histone cluster 2, H4a	1,53	3,26E-02
LAPTM4B	lysosomal protein transmembrane 4 beta	1,52	2,81E-04
NDRG4	NDRG family member 4	1,52	2,55E-03
LCTL	lactase-like	1,52	2,89E-03
SORD	sorbitol dehydrogenase	1,52	2,84E-03
PLLP	plasmolipin	1,52	1,62E-02
PM20D2	peptidase M20 domain containing 2	1,51	2,68E-02
CENPE	centromere protein E, 312kDa	1,51	4,02E-02
LYPLA1	lysophospholipase I	1,51	3,38E-03
NDRG2	NDRG family member 2	1,51	1,34E-03
EYA1	eyes absent homolog 1 (Drosophila)	1,51	4,93E-02
LRFN5	leucine rich repeat and fibronectin type III domain containing 5	1,51	4,30E-02
HSPB8	heat shock 22kDa protein 8	1,51	3,22E-02
ZC4H2	zinc finger, C4H2 domain containing	1,51	4,24E-03
MAP2K6	mitogen-activated protein kinase kinase 6	1,51	3,06E-02
CLYBL	citrate lyase beta like	1,50	4,38E-02
PLEKHA2	pleckstrin homology domain containing, family A (phosphoinositide binding specific) member 2	-1,50	1,52E-02
DNAJB4	DnaJ (Hsp40) homolog, subfamily B, member 4	-1,50	3,67E-03
CBLN3	cerebellin 3 precursor	-1,50	1,30E-03
RND3	Rho family GTPase 3	-1,51	4,45E-02
LRRC2	leucine rich repeat containing 2	-1,51	2,76E-02
DOCK11	dedicator of cytokinesis 11	-1,51	1,98E-02
B4GALT1	UDP-Gal:betaGlcNAc beta 1,4- galactosyltransferase, polypeptide 1	-1,51	3,80E-02
TPP1	tripeptidyl peptidase I	-1,51	5,50E-03
H6PD	hexose-6-phosphate dehydrogenase (glucose 1-dehydrogenase)	-1,51	6,97E-03
ANXA4	annexin A4	-1,51	3,93E-02
PARD3B	par-3 partitioning defective 3 homolog B (C. elegans)	-1,51	1,45E-03
CBX6	chromobox homolog 6	-1,52	1,41E-03
C4orf34	chromosome 4 open reading frame 34	-1,52	7,98E-03
JAZF1	JAZF zinc finger 1	-1,52	1,78E-02
C6orf89	chromosome 6 open reading frame 89	-1,52	2,50E-03
ZNFX1	zinc finger, NFX1-type containing 1	-1,52	8,37E-04
ABCC3	ATP-binding cassette, sub-family C (CFTR/MRP), member 3	-1,52	1,44E-02
C5orf53	chromosome 5 open reading frame 53	-1,53	1,47E-03
ZNF677	zinc finger protein 677	-1,53	6,30E-04
FHL1	four and a half LIM domains 1	-1,54	1,96E-02
ZHX3	zinc fingers and homeoboxes 3	-1,54	5,40E-03
TNXB	tenascin XB	-1,54	2,33E-03
ATP2B1	ATPase, Ca++ transporting, plasma membrane 1	-1,54	9,92E-03
SYNPO2	synaptopodin 2	-1,55	1,99E-02
PKD2	polycystic kidney disease 2 (autosomal dominant)	-1,55	2,37E-02
DPP7	dipeptidyl-peptidase 7	-1,55	5,65E-04
IRAK4	interleukin-1 receptor-associated kinase 4	-1,55	7,51E-03
ZNF404	zinc finger protein 404	-1,55	2,09E-02
SERTAD2	SERTA domain containing 2	-1,56	5,56E-03
ITPRIPL2	inositol 1,4,5-triphosphate receptor interacting protein-like 2	-1,56	3,87E-02
MMP19	matrix metallopeptidase 19	-1,56	1,41E-02
SCP2	sterol carrier protein 2	-1,56	4,74E-03
SLFN5	schlafen family member 5	-1,56	3,22E-02
MUC1	mucin 1, cell surface associated	-1,56	1,08E-02
FAS	Fas (TNF receptor superfamily, member 6)	-1,57	4,38E-02
ZNF577	zinc finger protein 577	-1,57	1,43E-02
DOCK5	dedicator of cytokinesis 5	-1,58	1,75E-02
ZNF876P	zinc finger protein 876, pseudogene	-1,58	1,23E-03
QSOX1	quiescin Q6 sulfhydryl oxidase 1	-1,58	1,97E-03
ZNF90	zinc finger protein 90	-1,58	3,69E-02
SYBU	syntabulin (syntaxin-interacting)	-1,58	5,16E-03
LOC10012903	QIQN5815	-1,58	1,04E-02
PODNL1	podocan-like 1	-1,59	1,00E-02
ABCA2	ATP-binding cassette, sub-family A (ABC1), member 2	-1,59	2,50E-03
BACE1	beta-site APP-cleaving enzyme 1	-1,59	1,27E-02
PRUNE2	prune homolog 2 (Drosophila)	-1,59	1,64E-02
ZCCHC24	zinc finger, CCHC domain containing 24	-1,59	2,26E-03
GABPB2	GA binding protein transcription factor, beta subunit 2	-1,60	6,93E-04
NFATC4	nuclear factor of activated T-cells, cytoplasmic, calcineurin-dependent 4	-1,60	6,33E-03
SLC6A9	solute carrier family 6 (neurotransmitter transporter, glycine), member 9	-1,60	1,91E-02
NCOA7	nuclear receptor coactivator 7	-1,60	2,03E-02

PLAGL1	pleiomorphic adenoma gene-like 1	-1,60	4,72E-02
ZNF493	zinc finger protein 493	-1,60	9,12E-04
TMEM140	transmembrane protein 140	-1,60	2,76E-02
CCDC53	coiled-coil domain containing 53	-1,60	6,84E-04
NFIB	nuclear factor I/B	-1,61	1,53E-02
MBNL1	muscleblind-like (Drosophila)	-1,61	1,41E-02
CCRL1	chemokine (C-C motif) receptor-like 1	-1,61	4,45E-02
LOC10012798	hypothetical protein LOC100127980	-1,61	8,37E-04
THRA	thyroid hormone receptor, alpha (erythroblastic leukemia viral (v-erb-a) oncogene homolog, avian)	-1,61	6,40E-03
MAN1A1	mannosidase, alpha, class 1A, member 1	-1,61	9,33E-03
ACOX2	acyl-CoA oxidase 2, branched chain	-1,61	1,89E-03
HOXA11	homeobox A11	-1,61	2,40E-02
ZNF772	zinc finger protein 772	-1,61	1,44E-02
ATP2B4	ATPase, Ca++ transporting, plasma membrane 4	-1,62	3,83E-03
CYR61	cysteine-rich, angiogenic inducer, 61	-1,62	1,47E-02
PRRX2	paired related homeobox 2	-1,62	9,22E-05
ZNF415	zinc finger protein 415	-1,62	1,72E-02
STAT2	signal transducer and activator of transcription 2, 113kDa	-1,63	1,49E-03
OGN	osteoglycin	-1,63	4,46E-02
CTBS	chitobiase, di-N-acetyl-	-1,63	9,18E-03
MRC2	mannose receptor, C type 2	-1,63	4,32E-02
HSD3B7	hydroxy-delta-5-steroid dehydrogenase, 3 beta- and steroid delta-isomerase 7	-1,63	2,80E-03
TAOK3	TAO kinase 3	-1,63	4,44E-03
BTN3A1	butyrophilin, subfamily 3, member A1	-1,64	1,21E-02
FAT4	FAT tumor suppressor homolog 4 (Drosophila)	-1,64	1,63E-02
LOC375295	hypothetical LOC375295	-1,64	3,67E-02
LPPR2	lipid phosphate phosphatase-related protein type 2	-1,65	5,66E-05
PLAC9	placenta-specific 9	-1,65	6,38E-04
FBLN7	fibulin 7	-1,65	6,84E-04
CRIM1	cysteine rich transmembrane BMP regulator 1 (chordin-like)	-1,66	2,27E-02
XPNPEP2	X-prolyl aminopeptidase (aminopeptidase P) 2, membrane-bound	-1,66	1,21E-02
SIAE	sialic acid acetylesterase	-1,66	1,23E-03
HSPG2	heparan sulfate proteoglycan 2	-1,66	9,93E-03
ARSA	arylsulfatase A	-1,66	9,78E-03
ZNF502	zinc finger protein 502	-1,67	7,95E-05
MFSD1	major facilitator superfamily domain containing 1	-1,67	2,17E-02
CEBPD	CCAAT/enhancer binding protein (C/EBP), delta	-1,67	3,65E-04
C1RL	complement component 1, r subcomponent-like	-1,67	3,00E-03
NLRP10	NLR family, pyrin domain containing 10	-1,67	8,67E-03
PDE4DIP	phosphodiesterase 4D interacting protein	-1,67	1,06E-03
KGFLP1	keratinocyte growth factor-like protein 1	-1,68	5,26E-03
KIAA0427	KIAA0427	-1,68	4,44E-03
TRANK1	tetratricopeptide repeat and ankyrin repeat containing 1	-1,69	3,86E-03
SLC2A10	solute carrier family 2 (facilitated glucose transporter), member 10	-1,69	5,56E-04
SGK269	NKF3 kinase family member	-1,70	8,12E-04
CEBPB	CCAAT/enhancer binding protein (C/EBP), beta	-1,70	1,31E-03
ZNF454	zinc finger protein 454	-1,71	1,38E-05
TSHZ1	teashirt zinc finger homeobox 1	-1,71	3,64E-02
NPR3	natriuretic peptide receptor C/guanylate cyclase C (atrionatriuretic peptide receptor C)	-1,71	1,93E-02
KLHDC1	kelch domain containing 1	-1,71	1,39E-02
CNTNAP1	contactin associated protein 1	-1,71	4,40E-03
PIGZ	phosphatidylinositol glycan anchor biosynthesis, class Z	-1,72	6,44E-03
RFX8	regulatory factor X, 8	-1,72	6,21E-03
KIAA1462	KIAA1462	-1,72	4,92E-02
LAMB2	laminin, beta 2 (laminin S)	-1,72	8,12E-04
KDELR3	KDEL (Lys-Asp-Glu-Leu) endoplasmic reticulum protein retention receptor 3	-1,72	1,93E-02
ST6GALNAC(	ST6 (alpha-N-acetyl-neuraminyl-2,3-beta-galactosyl-1,3)-N-acetylgalactosaminide alpha-2,6-sialyltransferase 6	-1,72	5,20E-03
TSHZ3	teashirt zinc finger homeobox 3	-1,72	4,20E-02
GSN	gelsolin	-1,72	3,13E-02
KIAA1644	KIAA1644	-1,72	1,47E-03
BCL6	B-cell CLL/lymphoma 6	-1,73	3,29E-02
ERCC6	excision repair cross-complementing rodent repair deficiency, complementation group 6	-1,73	7,24E-05
AGTR1	angiotensin II receptor, type 1	-1,73	6,61E-04
RGMB	RGM domain family, member B	-1,74	1,45E-02
ZNF461	zinc finger protein 461	-1,74	1,81E-02
C1QTNF5	C1q and tumor necrosis factor related protein 5	-1,74	4,04E-03
FLJ30064	hypothetical protein LOC644975	-1,74	4,49E-03
APPL2	adaptor protein, phosphotyrosine interaction, PH domain and leucine zipper containing 2	-1,74	3,27E-03
KAT2B	K(lysine) acetyltransferase 2B	-1,74	3,15E-02
CD97	CD97 molecule	-1,74	2,16E-02
NQO1	NAD(P)H dehydrogenase, quinone 1	-1,74	4,26E-02
TWIST1	twist homolog 1 (Drosophila)	-1,75	2,11E-02
HOXD8	homeobox D8	-1,75	9,30E-03
KRTAP1-1	keratin associated protein 1-1	-1,75	3,79E-02
LOC402778	CD225 family protein FLJ76511	-1,75	7,51E-03
DSEL	dermatan sulfate epimerase-like	-1,75	8,60E-03
C17orf91	chromosome 17 open reading frame 91	-1,75	2,18E-03
ATP10A	ATPase, class V, type 10A	-1,76	1,71E-03
TSPO	translocator protein (18kDa)	-1,76	1,76E-02
SETD7	SET domain containing (lysine methyltransferase) 7	-1,76	3,34E-02
HGF	hepatocyte growth factor (hepapoietin A; scatter factor)	-1,77	2,97E-02
FADS3	fatty acid desaturase 3	-1,77	1,65E-03
ZNF253	zinc finger protein 253	-1,77	7,73E-04
PARP9	poly (ADP-ribose) polymerase family, member 9	-1,78	4,96E-02
HAPLN3	hyaluronan and proteoglycan link protein 3	-1,78	2,10E-02
ATL3	atlastin GTPase 3	-1,78	5,76E-05
LMCD1	LIM and cysteine-rich domains 1	-1,78	4,70E-02
FOSL2	FOS-like antigen 2	-1,78	1,30E-02
ZNF429	zinc finger protein 429	-1,79	1,44E-02
CTSF	cathepsin F	-1,79	3,01E-03
ZDHHC1	zinc finger, DHHC-type containing 1	-1,79	5,57E-03
RASGRP3	RAS guanyl releasing protein 3 (calcium and DAG-regulated)	-1,79	3,62E-02
TCEA3	transcription elongation factor A (SII), 3	-1,80	1,44E-02
SH3PXD2A	SH3 and PX domains 2A	-1,80	1,06E-03
ADCY3	adenylate cyclase 3	-1,80	4,59E-03
VEGFB	vascular endothelial growth factor B	-1,80	5,90E-04
HIST2H2BF	histone cluster 2, H2bf	-1,80	1,12E-02
TMEM45A	transmembrane protein 45A	-1,80	1,38E-02
GMPR	guanosine monophosphate reductase	-1,80	4,48E-03
APOL1	apolipoprotein L, 1	-1,81	3,63E-05
HFE	hemochromatosis	-1,81	2,55E-04
LTBP1	latent transforming growth factor beta binding protein 1	-1,81	1,03E-02
ZNF25	zinc finger protein 25	-1,82	1,90E-02
ZBTB20	zinc finger and BTB domain containing 20	-1,82	4,44E-02
MVP	major vault protein	-1,82	1,66E-02
P4HA2	prolyl 4-hydroxylase, alpha polypeptide II	-1,82	3,44E-03
NUCB2	nucleobindin 2	-1,83	2,31E-03
SYNE1	spectrin repeat containing, nuclear envelope 1	-1,83	2,31E-03
PTGIR	prostaglandin I2 (prostacyclin) receptor (IP)	-1,84	7,14E-03
COL1A1	collagen, type I, alpha 1	-1,84	3,62E-02
CASP1	caspase 1, apoptosis-related cysteine peptidase (interleukin 1, beta, convertase)	-1,85	2,68E-02
STAT4	signal transducer and activator of transcription 4	-1,85	9,18E-03
DKK3	dickkopf homolog 3 (Xenopus laevis)	-1,85	1,62E-02
S100A4	S100 calcium binding protein A4	-1,85	2,52E-02
TMEM117	transmembrane protein 117	-1,85	1,23E-02
CTSL1	cathepsin L1	-1,86	3,76E-04
HOXC5	homeobox C5	-1,86	7,91E-05
HLA-F	major histocompatibility complex, class I, F	-1,86	6,21E-05
ZNF788	zinc finger family member 788	-1,86	1,52E-05
ANKH	ankylosis, progressive homolog (mouse)	-1,86	4,76E-03
ZNF559	zinc finger protein 559	-1,86	4,70E-04
SOD3	superoxide dismutase 3, extracellular	-1,87	1,95E-03
ZFP28	zinc finger protein 28 homolog (mouse)	-1,87	5,92E-03
HKR1	HKR1, GLI-Kruppel zinc finger family member	-1,87	5,01E-04
ACSL1	acyl-CoA synthetase long-chain family member 1	-1,87	1,97E-03
C11orf70	chromosome 11 open reading frame 70	-1,88	2,84E-04
SLC9A9	solute carrier family 9 (sodium/hydrogen exchanger), member 9	-1,88	2,93E-02
ZNF583	zinc finger protein 583	-1,88	3,39E-02
TBC1D2B	TBC1 domain family, member 2B	-1,88	8,13E-04
GDF5	growth differentiation factor 5	-1,88	2,41E-04
POPDC3	popeye domain containing 3	-1,88	2,37E-02
KANK2	KN motif and ankyrin repeat domains 2	-1,90	1,38E-04
SELM	selenoprotein M	-1,90	5,01E-04
KRTAP1-1	keratin associated protein 1-1	-1,90	6,38E-04

ZNF229	zinc finger protein 229	-1,90	1,38E-05
MFSD6	major facilitator superfamily domain containing 6	-1,90	8,57E-04
HOXA9	homeobox A9	-1,90	2,62E-03
FAM151B	family with sequence similarity 151, member B	-1,91	1,09E-02
IFIT2	interferon-induced protein with tetratricopeptide repeats 2	-1,92	4,90E-02
DKFZp686O24	hypothetical protein DKFZp686O24166	-1,92	1,74E-02
LRP1	low density lipoprotein receptor-related protein 1	-1,92	1,47E-03
RNH1	ribonuclease/angiogenin inhibitor 1	-1,92	9,96E-04
GYPE	glycophorin E (MNS blood group)	-1,92	6,30E-04
FAM176A	family with sequence similarity 176, member A	-1,92	7,43E-03
FAM69A	family with sequence similarity 69, member A	-1,92	2,50E-03
CFB	complement factor B	-1,93	1,97E-03
EHD2	EH-domain containing 2	-1,93	1,21E-03
KITLG	KIT ligand	-1,93	1,57E-02
ASAM	adipocyte-specific adhesion molecule	-1,93	1,61E-02
GSTM3	glutathione S-transferase mu 3 (brain)	-1,93	2,80E-02
SLC46A3	solute carrier family 46, member 3	-1,94	4,90E-02
HEG1	HEG homolog 1 (zebrafish)	-1,94	4,67E-02
ACVRL1	activin A receptor type II-like 1	-1,94	5,31E-03
EFEMP2	EGF-containing fibulin-like extracellular matrix protein 2	-1,94	1,47E-02
C6orf145	chromosome 6 open reading frame 145	-1,94	2,29E-02
PCDHGB4	protocadherin gamma subfamily B, 4	-1,95	2,83E-02
TMEM204	transmembrane protein 204	-1,95	3,74E-03
PTGS2	prostaglandin-endoperoxide synthase 2 (prostaglandin G/H synthase and cyclooxygenase)	-1,96	4,29E-02
AHNAK	AHNAK nucleoprotein	-1,96	7,13E-03
ZNF283	zinc finger protein 283	-1,96	7,05E-04
PSG3	pregnancy specific beta-1-glycoprotein 3	-1,96	3,29E-03
PTGES	prostaglandin E synthase	-1,96	8,18E-05
PTRF	polymerase I and transcript release factor	-1,96	1,30E-02
ZFP3	zinc finger protein 3 homolog (mouse)	-1,97	5,44E-05
RARG	retinoic acid receptor, gamma	-1,97	8,28E-03
NTF3	neurotrophin 3	-1,97	9,76E-04
DAAM2	dishevelled associated activator of morphogenesis 2	-1,98	1,34E-02
ZNF568	zinc finger protein 568	-1,98	3,74E-05
TTC39C	tetratricopeptide repeat domain 39C	-1,99	1,29E-03
TRIM38	tripartite motif-containing 38	-1,99	2,33E-02
KCNMA1	potassium large conductance calcium-activated channel, subfamily M, alpha member 1	-2,00	2,72E-02
ADCY9	adenylate cyclase 9	-2,00	4,02E-04
ZNF595	zinc finger protein 595	-2,00	2,09E-05
B3GNT9	UDP-GlcNAc:betaGal beta-1,3-N-acetylglucosaminyltransferase 9	-2,00	3,47E-05
LRRN4CL	LRRN4 C-terminal like	-2,00	1,70E-02
C21orf7	chromosome 21 open reading frame 7	-2,00	2,86E-02
AVPI1	arginine vasopressin-induced 1	-2,01	1,97E-03
STAT1	signal transducer and activator of transcription 1, 91kDa	-2,01	3,26E-03
PLA2R1	phospholipase A2 receptor 1, 180kDa	-2,02	1,31E-02
PRKD1	protein kinase D1	-2,02	2,05E-02
ADAM33	ADAM metallopeptidase domain 33	-2,02	2,38E-05
REV3L	REV3-like, catalytic subunit of DNA polymerase zeta (yeast)	-2,02	1,09E-02
CTSO	cathepsin O	-2,03	3,33E-02
ADAMTSL1	ADAMTS-like 1	-2,03	4,69E-02
TK2	thymidine kinase 2, mitochondrial	-2,04	3,32E-04
TBC1D2	TBC1 domain family, member 2	-2,04	1,24E-02
STARD5	StAR-related lipid transfer (START) domain containing 5	-2,04	3,98E-03
TWIST2	twist homolog 2 (Drosophila)	-2,04	7,60E-03
LAYN	layilin	-2,04	2,66E-02
PMP22	peripheral myelin protein 22	-2,05	7,87E-03
IFI35	interferon-induced protein 35	-2,05	7,87E-03
TBC1D2B	TBC1 domain family, member 2B	-2,05	3,14E-03
CYP4V2	cytochrome P450, family 4, subfamily V, polypeptide 2	-2,05	8,90E-03
RPS6KA2	ribosomal protein S6 kinase, 90kDa, polypeptide 2	-2,07	3,49E-03
CCPG1	cell cycle progression 1	-2,08	1,50E-02
MAN2B2	mannosidase, alpha, class 2B, member 2	-2,08	2,63E-03
NEGR1	neuronal growth regulator 1	-2,08	5,84E-03
HIST1H2BB	histone cluster 1, H2bb	-2,08	4,10E-03
ZNF135	zinc finger protein 135	-2,09	2,50E-04
NR3C1	nuclear receptor subfamily 3, group C, member 1 (glucocorticoid receptor)	-2,10	1,27E-04
HCFC2	host cell factor C2	-2,10	1,19E-02
TNFSF4	tumor necrosis factor (ligand) superfamily, member 4	-2,10	3,78E-02
CD109	CD109 molecule	-2,10	9,84E-03
SSPN	sarcospan (Kras oncogene-associated gene)	-2,11	9,84E-03
TENC1	tensin like C1 domain containing phosphatase (tensin 2)	-2,11	1,99E-02
SERPINE2	serpin peptidase inhibitor, clade E (nexin, plasminogen activator inhibitor type 1), member 2	-2,12	8,60E-03
SMPD1	sphingomyelin phosphodiesterase 1, acid lysosomal	-2,12	1,11E-02
GEM	GTP binding protein overexpressed in skeletal muscle	-2,13	1,43E-02
ALPK1	alpha-kinase 1	-2,13	1,79E-03
TRIB3	tribbles homolog 3 (Drosophila)	-2,13	2,18E-02
C10orf54	chromosome 10 open reading frame 54	-2,13	8,13E-04
GABRE	gamma-aminobutyric acid (GABA) A receptor, epsilon	-2,13	8,72E-04
NFASC	neurofascin	-2,14	6,36E-03
BEND6	BEN domain containing 6	-2,14	8,92E-04
STS	steroid sulfatase (microsomal), isozyme S	-2,14	2,15E-02
SMARCA2	SWI/SNF related, matrix associated, actin dependent regulator of chromatin, subfamily a, member 2	-2,15	3,22E-03
IL6ST	interleukin 6 signal transducer (gp130, oncostatin M receptor)	-2,15	4,32E-02
CYTH3	cytohesin 3	-2,15	6,74E-04
ADAMTSL5	ADAMTS-like 5	-2,15	2,12E-04
ARHGAP20	Rho GTPase activating protein 20	-2,15	2,74E-03
CREB3L1	cAMP responsive element binding protein 3-like 1	-2,16	7,62E-03
PPIC	peptidylprolyl isomerase C (cyclophilin C)	-2,17	3,44E-03
CRISPLD2	cysteine-rich secretory protein LCCL domain containing 2	-2,17	4,73E-03
ZFP82	zinc finger protein 82 homolog (mouse)	-2,17	4,31E-03
MGLL	monoglyceride lipase	-2,18	4,08E-02
C5orf23	chromosome 5 open reading frame 23	-2,19	7,73E-03
PLCE1	phospholipase C, epsilon 1	-2,19	2,62E-02
PCOLCE	procollagen C-endopeptidase enhancer	-2,19	1,03E-02
GLT8D2	glycosyltransferase 8 domain containing 2	-2,19	3,34E-03
TCP11L2	t-complex 11 (mouse)-like 2	-2,20	5,02E-03
C14orf49	chromosome 14 open reading frame 49	-2,21	2,38E-05
ZNF844	zinc finger protein 844	-2,22	7,55E-05
TIMP2	TIMP metallopeptidase inhibitor 2	-2,22	1,64E-02
GRAMD3	GRAM domain containing 3	-2,22	2,81E-03
IL10RB	interleukin 10 receptor, beta	-2,23	1,67E-03
CD55	CD55 molecule, decay accelerating factor for complement (Cromer blood group)	-2,23	3,99E-02
ARHGAP24	Rho GTPase activating protein 24	-2,23	5,01E-04
EVI2B	ecotropic viral integration site 2B	-2,23	8,20E-04
SNORD113-3	small nucleolar RNA, C/D box 113-3	-2,24	9,97E-04
RCN3	reticulocalbin 3, EF-hand calcium binding domain	-2,24	8,94E-03
EBF3	early B-cell factor 3	-2,24	1,12E-02
TNS1	tensin 1	-2,24	1,51E-02
COL14A1	collagen, type XIV, alpha 1	-2,26	1,64E-02
COL6A1	collagen, type VI, alpha 1	-2,26	2,46E-03
PGCP	plasma glutamate carboxypeptidase	-2,26	1,43E-02
IFI44	interferon-induced protein 44	-2,26	1,95E-02
THBS1	thrombospondin 1	-2,26	1,93E-02
SPATA18	spermatogenesis associated 18 homolog (rat)	-2,27	1,89E-02
ACTA2	actin, alpha 2, smooth muscle, aorta	-2,27	3,29E-02
HOXC9	homeobox C9	-2,27	1,19E-03
LPXN	leupaxin	-2,28	4,69E-02
RBMS3	RNA binding motif, single stranded interacting protein 3	-2,28	4,10E-02
PAMR1	peptidase domain containing associated with muscle regeneration 1	-2,28	2,74E-02
ADAMTS5	ADAM metallopeptidase with thrombospondin type 1 motif, 5	-2,29	4,74E-02
LRRK2	leucine-rich repeat kinase 2	-2,29	2,19E-04
STOM	stomatin	-2,29	2,13E-02
PRKG1	protein kinase, cGMP-dependent, type I	-2,29	6,24E-03
IDUA	iduronidase, alpha-L-	-2,29	1,18E-04
LAMA2	laminin, alpha 2	-2,29	3,43E-02
PTGFR	prostaglandin F receptor (FP)	-2,30	1,10E-02
KCNK15	potassium channel, subfamily K, member 15	-2,30	1,26E-02
VEPH1	ventricular zone expressed PH domain homolog 1 (zebrafish)	-2,30	3,94E-02
CXCL12	chemokine (C-X-C motif) ligand 12	-2,30	2,47E-02
LTBP3	latent transforming growth factor beta binding protein 3	-2,30	7,10E-05
APCDD1	adenomatosis polyposis coli down-regulated 1	-2,30	3,95E-02
ABLIM3	actin binding LIM protein family, member 3	-2,31	2,39E-03
CSF1	colony stimulating factor 1 (macrophage)	-2,31	9,76E-04
PARP3	poly (ADP-ribose) polymerase family, member 3	-2,31	4,02E-04

PSG7	pregnancy specific beta-1-glycoprotein 7 (gene/pseudogene)	-2,32	1,60E-02
RPSAP52	ribosomal protein SA pseudogene 52	-2,32	3,81E-03
GALNTL1	UDP-N-acetyl-alpha-D-galactosamine:polypeptide N-acetylgalactosaminyltransferase-like 1	-2,35	2,57E-03
LYST	lysosomal trafficking regulator	-2,35	8,24E-03
C14orf139	chromosome 14 open reading frame 139	-2,35	2,50E-04
ADAMTS1	ADAM metallopeptidase with thrombospondin type 1 motif, 1	-2,36	3,98E-02
GLRB	glycine receptor, beta	-2,38	1,18E-02
HTRA1	HtrA serine peptidase 1	-2,38	5,28E-03
ACAN	aggrecan	-2,38	7,51E-03
COL6A2	collagen, type VI, alpha 2	-2,39	8,69E-04
BMPER	BMP binding endothelial regulator	-2,39	2,29E-02
ZNF615	zinc finger protein 615	-2,39	3,03E-03
PCGF5	polycomb group ring finger 5	-2,40	3,36E-03
CCDC144A	coiled-coil domain containing 144A	-2,40	1,49E-04
VASN	vasorin	-2,40	3,74E-05
AKR1C3	aldo-keto reductase family 1, member C3 (3-alpha hydroxysteroid dehydrogenase, type II)	-2,40	3,72E-04
PDE1A	phosphodiesterase 1A, calmodulin-dependent	-2,41	1,82E-02
HOXC6	homeobox C6	-2,41	1,92E-03
AEBP1	AE binding protein 1	-2,41	1,46E-03
ARRDC4	arrestin domain containing 4	-2,41	2,11E-02
ECM1	extracellular matrix protein 1	-2,41	1,28E-02
LRRC32	leucine rich repeat containing 32	-2,41	1,85E-02
FKBP9	FK506 binding protein 9, 63 kDa	-2,42	1,64E-02
MYLK	myosin light chain kinase	-2,42	1,52E-02
COL12A1	collagen, type XII, alpha 1	-2,42	2,17E-02
TNFSF18	tumor necrosis factor (ligand) superfamily, member 18	-2,43	1,54E-03
COL1A2	collagen, type I, alpha 2	-2,43	2,09E-02
DRAM1	DNA-damage regulated autophagy modulator 1	-2,43	7,31E-03
B2M	beta-2-microglobulin	-2,44	6,23E-03
STOM	stomatin	-2,44	4,90E-02
TLR3	toll-like receptor 3	-2,45	2,55E-03
PRSS12	protease, serine, 12 (neurotrypsin, motopsin)	-2,45	8,79E-03
NAAA	N-acylethanolamine acid amidase	-2,46	5,56E-04
PTGS1	prostaglandin-endoperoxide synthase 1 (prostaglandin G/H synthase and cyclooxygenase)	-2,46	1,46E-03
ZNF93	zinc finger protein 93	-2,46	3,26E-05
PPAP2B	phosphatidic acid phosphatase type 2B	-2,47	8,47E-03
SLC30A4	solute carrier family 30 (zinc transporter), member 4	-2,49	3,43E-02
ZNF350	zinc finger protein 350	-2,50	1,38E-05
MAP1A	microtubule-associated protein 1A	-2,50	2,43E-02
CALHM2	calcium homeostasis modulator 2	-2,50	7,91E-05
PXK	PX domain containing serine/threonine kinase	-2,52	8,35E-04
DOK5	docking protein 5	-2,53	1,31E-03
OXTR	oxytocin receptor	-2,53	4,07E-02
ZFP36	zinc finger protein 36, C3H type, homolog (mouse)	-2,53	6,75E-03
HOXC8	homeobox C8	-2,54	1,24E-02
ZNF585A	zinc finger protein 585A	-2,54	2,39E-06
PDZRN3	PDZ domain containing ring finger 3	-2,54	7,43E-03
SEMA5A	sema domain, seven thrombospondin repeats (type 1 and type 1-like), transmembrane domain (TM) and short cytoplasmic domain, (semap	-2,55	1,19E-02
GBP3	guanylate binding protein 3	-2,55	4,03E-02
SGMS2	sphingomyelin synthase 2	-2,55	2,57E-03
CARD6	caspase recruitment domain family, member 6	-2,56	6,14E-03
VGLL3	vestigial like 3 (Drosophila)	-2,58	8,37E-03
ZNF880	zinc finger protein 880	-2,58	3,26E-05
HSPB7	heat shock 27kDa protein family, member 7 (cardiovascular)	-2,60	1,19E-02
MSRB3	methionine sulfoxide reductase B3	-2,61	6,20E-03
CACNA1C	calcium channel, voltage-dependent, L type, alpha 1C subunit	-2,61	1,03E-03
BTN3A3	butyrophilin, subfamily 3, member A3	-2,61	7,95E-05
ANKRD29	ankyrin repeat domain 29	-2,65	4,95E-03
EMX2	empty spiracles homeobox 2	-2,65	4,14E-04
CLEC2B	C-type lectin domain family 2, member B	-2,66	1,30E-02
SLC16A7	solute carrier family 16, member 7 (monocarboxylic acid transporter 2)	-2,67	2,08E-04
CD68	CD68 molecule	-2,68	4,63E-04
LRRK1	leucine-rich repeat kinase 1	-2,68	2,46E-05
SIM1	single-minded homolog 1 (Drosophila)	-2,68	1,71E-02
AOX1	aldehyde oxidase 1	-2,69	6,13E-04
LBH	limb bud and heart development homolog (mouse)	-2,70	1,10E-03
ZNF542	zinc finger protein 542	-2,71	8,53E-03
PRR16	proline rich 16	-2,72	1,66E-04
KCNQ5	potassium voltage-gated channel, KQT-like subfamily, member 5	-2,72	3,29E-02
KCND3	potassium voltage-gated channel, Shal-related subfamily, member 3	-2,73	2,50E-03
CLIP4	CAP-GLY domain containing linker protein family, member 4	-2,74	7,67E-03
CERCAM	cerebral endothelial cell adhesion molecule	-2,74	3,56E-03
NPAS2	neuronal PAS domain protein 2	-2,76	3,65E-04
MYO1D	myosin ID	-2,76	1,46E-03
ZNF680	zinc finger protein 680	-2,77	6,28E-06
ENG	endoglin	-2,78	1,48E-02
OSR1	odd-skipped related 1 (Drosophila)	-2,79	8,66E-05
PDGFRA	platelet-derived growth factor receptor, alpha polypeptide	-2,79	1,31E-02
ZNF383	zinc finger protein 383	-2,80	4,32E-03
FAM129A	family with sequence similarity 129, member A	-2,81	3,61E-03
GPR124	G protein-coupled receptor 124	-2,81	8,05E-03
LTBP2	latent transforming growth factor beta binding protein 2	-2,81	3,55E-02
CYP1B1	cytochrome P450, family 1, subfamily B, polypeptide 1	-2,81	2,44E-02
ZNF626	zinc finger protein 626	-2,82	1,97E-05
NEK7	NIMA (never in mitosis gene a)-related kinase 7	-2,83	1,49E-03
LRRC15	leucine rich repeat containing 15	-2,83	3,43E-03
LTBR	lymphotoxin beta receptor (TNFR superfamily, member 3)	-2,83	9,46E-04
SQRDL	sulfide quinone reductase-like (yeast)	-2,83	4,88E-02
FMN2	formin 2	-2,85	5,56E-04
DAB2	disabled homolog 2, mitogen-responsive phosphoprotein (Drosophila)	-2,86	2,07E-02
ZNF486	zinc finger protein 486	-2,86	9,28E-03
CA12	carbonic anhydrase XII	-2,87	6,98E-04
TGFBI	transforming growth factor, beta-induced, 68kDa	-2,87	2,12E-02
SYT11	synaptotagmin XI	-2,87	1,98E-02
HOXC10	homeobox C10	-2,88	1,73E-02
NFIC	nuclear factor I/C (CCAAT-binding transcription factor)	-2,91	2,74E-04
SNED1	sushi, nidogen and EGF-like domains 1	-2,91	5,01E-04
XG	Xg blood group	-2,93	7,50E-03
OSMR	oncostatin M receptor	-2,93	4,53E-02
NFIA	nuclear factor I/A	-2,93	1,01E-02
SHOX2	short stature homeobox 2	-2,94	3,86E-05
LOXL4	lysyl oxidase-like 4	-2,95	8,05E-03
ADAMTS2	ADAM metallopeptidase with thrombospondin type 1 motif, 2	-2,96	5,23E-04
CD302	CD302 molecule	-2,96	2,56E-03
LMOD1	leiomodin 1 (smooth muscle)	-2,96	1,94E-04
CMAH	cytidine monophosphate-N-acetylneuraminic acid hydroxylase (CMP-N-acetylneuraminate monooxygenase) pseudogene	-2,98	1,08E-03
SYNC	syncoilin, intermediate filament protein	-2,98	1,14E-04
IRAK3	interleukin-1 receptor-associated kinase 3	-2,99	1,28E-03
KCNE4	potassium voltage-gated channel, Isk-related family, member 4	-3,00	9,95E-03
KLF4	Kruppel-like factor 4 (gut)	-3,01	4,02E-04
CLDN11	claudin 11	-3,02	5,79E-04
ELN	elastin	-3,03	5,56E-03
COL3A1	collagen, type III, alpha 1	-3,03	2,55E-02
SNORD114-2	small nucleolar RNA, C/D box 114-2	-3,04	1,94E-04
LGALS3	lectin, galactoside-binding, soluble, 3	-3,04	2,54E-05
COL15A1	collagen, type XV, alpha 1	-3,04	5,99E-03
KRT34	keratin 34	-3,04	3,12E-05
ADAM12	ADAM metallopeptidase domain 12	-3,06	4,99E-02
LPAR1	lysophosphatidic acid receptor 1	-3,07	5,90E-04
C10orf72	chromosome 10 open reading frame 72	-3,08	1,74E-03
SH2D4A	SH2 domain containing 4A	-3,08	2,19E-04
IL6	interleukin 6 (interferon, beta 2)	-3,09	3,06E-03
NIPAL2	NIPA-like domain containing 2	-3,10	9,76E-04
GALNT5	UDP-N-acetyl-alpha-D-galactosamine:polypeptide N-acetylgalactosaminyltransferase 5 (GalNAc-T5)	-3,10	1,43E-02
RECK	reversion-inducing-cysteine-rich protein with kazal motifs	-3,11	1,90E-03
EPAS1	endothelial PAS domain protein 1	-3,11	8,13E-03
ANGPTL2	angiopoietin-like 2	-3,12	1,42E-03
XAF1	XIAP associated factor 1	-3,12	2,82E-03
DPP4	dipeptidyl-peptidase 4	-3,12	4,82E-02
ZNF841	zinc finger protein 841	-3,15	1,26E-06
C9orf150	chromosome 9 open reading frame 150	-3,17	5,82E-03
VEGFC	vascular endothelial growth factor C	-3,18	4,11E-04
LPPR4	lipid phosphate phosphatase-related protein type 4	-3,19	1,31E-02
AKR1C2	aldo-keto reductase family 1, member C2 (dihydrodiol dehydrogenase 2; bile acid binding protein; 3-alpha hydroxysteroid dehydrogenase,	-3,23	1,18E-04

C7orf58	chromosome 7 open reading frame 58	-3,23	2,11E-02
KRTAP1-5	keratin associated protein 1-5	-3,24	2,29E-03
FIBIN	fin bud initiation factor homolog (zebrafish)	-3,26	6,39E-04
NAALADL2	N-acetylated alpha-linked acidic dipeptidase-like 2	-3,27	5,57E-05
UBA7	ubiquitin-like modifier activating enzyme 7	-3,27	3,63E-05
MKX	mohawk homeobox	-3,29	4,64E-02
NRN1	neuritin 1	-3,29	2,95E-02
VDR	vitamin D (1,25- dihydroxyvitamin D3) receptor	-3,30	5,55E-05
ZNF528	zinc finger protein 528	-3,32	5,97E-06
SPOCK1	sparc/osteonectin, cwcv and kazal-like domains proteoglycan (testican) 1	-3,32	6,01E-03
DPYD	dihydropyrimidine dehydrogenase	-3,33	3,15E-03
CD248	CD248 molecule, endosialin	-3,36	1,34E-02
OMD	osteomodulin	-3,36	9,03E-03
SRGN	serglycin	-3,36	1,97E-02
GSTM5	glutathione S-transferase mu 5	-3,41	2,76E-02
SERPINF1	serpin peptidase inhibitor, clade F (alpha-2 antiplasmin, pigment epithelium derived factor), member 1	-3,42	3,06E-03
SEMA3D	sema domain, immunoglobulin domain (Ig), short basic domain, secreted, (semaphorin) 3D	-3,42	3,90E-03
GBP1	guanylate binding protein 1, interferon-inducible, 67kDa	-3,43	3,95E-02
NOV	nephroblastoma overexpressed gene	-3,48	4,25E-04
CFB	complement factor B	-3,50	2,04E-04
SGCD	sarcoglycan, delta (35kDa dystrophin-associated glycoprotein)	-3,50	3,57E-03
TMEM173	transmembrane protein 173	-3,51	1,24E-03
GPR1	G protein-coupled receptor 1	-3,56	3,12E-04
ECM2	extracellular matrix protein 2, female organ and adipocyte specific	-3,56	8,27E-04
TGFBR3	transforming growth factor, beta receptor III	-3,57	5,85E-03
FBN1	fibrillin 1	-3,58	1,53E-02
ZNF506	zinc finger protein 506	-3,59	9,20E-06
AKR1C1	aldo-keto reductase family 1, member C1 (dihydrodiol dehydrogenase 1; 20-alpha (3-alpha)-hydroxysteroid dehydrogenase)	-3,60	1,38E-05
PTX3	pentraxin 3, long	-3,62	3,40E-02
IL1R1	interleukin 1 receptor, type I	-3,63	4,33E-02
SVEP1	sushi, von Willebrand factor type A, EGF and pentraxin domain containing 1	-3,63	6,38E-04
APOD	apolipoprotein D	-3,64	5,60E-03
ISLR	immunoglobulin superfamily containing leucine-rich repeat	-3,65	2,38E-05
WNT2	wingless-type MMTV integration site family member 2	-3,66	1,51E-02
MOXD1	monooxygenase, DBH-like 1	-3,68	2,32E-03
CCDC80	coiled-coil domain containing 80	-3,69	5,03E-03
SLFN11	schlafen family member 11	-3,69	5,68E-03
AK5	adenylate kinase 5	-3,69	9,12E-04
CCBE1	collagen and calcium binding EGF domains 1	-3,70	1,23E-03
FBLN2	fibulin 2	-3,70	2,43E-05
SECTM1	secreted and transmembrane 1	-3,71	6,16E-04
SAMD9	sterile alpha motif domain containing 9	-3,71	1,76E-02
LOC554202	hypothetical LOC554202	-3,72	9,70E-05
TBX15	T-box 15	-3,72	2,08E-04
ATP8B1	ATPase, aminophospholipid transporter, class I, type 8B, member 1	-3,73	1,08E-03
VTRNA1-1	vault RNA 1-1	-3,75	3,81E-03
ABCA6	ATP-binding cassette, sub-family A (ABC1), member 6	-3,76	5,49E-03
NTN4	netrin 4	-3,76	2,08E-03
COPZ2	coatomer protein complex, subunit zeta 2	-3,76	5,79E-04
SNORD114-26	small nucleolar RNA, C/D box 114-26	-3,78	5,97E-06
DKK1	dickkopf homolog 1 (Xenopus laevis)	-3,80	2,80E-02
ZNF585B	zinc finger protein 585B	-3,80	3,63E-05
ALDH1L2	aldehyde dehydrogenase 1 family, member L2	-3,80	5,22E-04
APOL6	apolipoprotein L, 6	-3,81	1,54E-03
EBF1	early B-cell factor 1	-3,82	5,68E-03
PRELP	proline/arginine-rich end leucine-rich repeat protein	-3,85	1,40E-03
THBS2	thrombospondin 2	-3,88	1,39E-03
PPP1R3C	protein phosphatase 1, regulatory (inhibitor) subunit 3C	-3,88	2,10E-04
SULF1	sulfatase 1	-3,88	5,02E-03
ZNF717	zinc finger protein 717	-3,91	2,43E-05
LMO7	LIM domain 7	-3,96	1,52E-05
PLSCR4	phospholipid scramblase 4	-3,97	1,34E-04
CAT	catalase	-3,98	6,28E-06
GREM2	gremlin 2	-4,01	2,13E-03
KGFLP1	keratinocyte growth factor-like protein 1	-4,04	3,74E-05
TNXB	tenascin XB	-4,09	1,22E-04
MSC	musculin	-4,12	1,74E-03
FAM180A	family with sequence similarity 180, member A	-4,13	5,01E-04
OSR2	odd-skipped related 2 (Drosophila)	-4,13	1,43E-02
PSG5	pregnancy specific beta-1-glycoprotein 5	-4,15	3,01E-03
LUM	lumican	-4,18	1,67E-02
PRRX1	paired related homeobox 1	-4,18	3,44E-03
CASP4	caspase 4, apoptosis-related cysteine peptidase	-4,18	3,52E-06
MEG3	maternally expressed 3 (non-protein coding)	-4,20	3,45E-07
LAMA4	laminin, alpha 4	-4,24	1,89E-02
ENPP2	ectonucleotide pyrophosphatase/phosphodiesterase 2	-4,25	5,59E-03
LY96	lymphocyte antigen 96	-4,29	2,04E-04
MR1	major histocompatibility complex, class I-related	-4,30	6,24E-07
TMEM119	transmembrane protein 119	-4,43	7,87E-05
ANPEP	alanyl (membrane) aminopeptidase	-4,45	1,61E-03
TNFRSF11B	tumor necrosis factor receptor superfamily, member 11b	-4,46	6,13E-04
CYBRD1	cytochrome b reductase 1	-4,49	2,32E-04
SERPINB2	serpin peptidase inhibitor, clade B (ovalbumin), member 2	-4,50	5,59E-03
FMOD	fibromodulin	-4,52	1,60E-04
ZNF737	zinc finger protein 737	-4,54	6,56E-03
POSTN	periostin, osteoblast specific factor	-4,54	1,92E-02
DDR2	discoidin domain receptor tyrosine kinase 2	-4,56	1,65E-03
CRYAB	crystallin, alpha B	-4,56	6,27E-04
CCRL1	chemokine (C-C motif) receptor-like 1	-4,56	4,22E-02
FGF5	fibroblast growth factor 5	-4,57	8,14E-03
FBLN5	fibulin 5	-4,58	6,05E-06
FGF7	fibroblast growth factor 7	-4,59	1,38E-05
PODN	podocan	-4,72	2,39E-06
SLIT3	slit homolog 3 (Drosophila)	-4,80	1,66E-04
DDR2	discoidin domain receptor tyrosine kinase 2	-4,83	6,38E-04
HSPB6	heat shock protein, alpha-crystallin-related, B6	-4,84	1,23E-04
SNORD113-4	small nucleolar RNA, C/D box 113-4	-4,85	5,97E-06
SAMD9L	sterile alpha motif domain containing 9-like	-4,87	2,52E-03
C1R	complement component 1, r subcomponent	-4,88	1,37E-05
C1S	complement component 1, s subcomponent	-4,89	6,41E-06
NUPR1	nuclear protein, transcriptional regulator, 1	-4,90	5,02E-06
CTSK	cathepsin K	-4,91	1,42E-06
COL6A3	collagen, type VI, alpha 3	-4,92	9,88E-05
MFAP4	microfibrillar-associated protein 4	-4,99	4,02E-04
NFIX	nuclear factor I/X (CCAAT-binding transcription factor)	-5,03	1,16E-06
OLFML1	olfactomedin-like 1	-5,04	8,12E-04
ITGBL1	integrin, beta-like 1 (with EGF-like repeat domains)	-5,31	1,37E-05
KIAA1199	KIAA1199	-5,45	9,76E-04
ASPN	asporin	-5,47	3,61E-04
KCNK2	potassium channel, subfamily K, member 2	-5,59	6,41E-06
FAP	fibroblast activation protein, alpha	-5,85	5,02E-06
LOC10028811	hypothetical LOC100288114	-6,04	3,99E-08
GPNMB	glycoprotein (transmembrane) nmb	-6,20	1,04E-05
DCN	decorin	-6,34	1,33E-03
SNORD114-3	small nucleolar RNA, C/D box 114-3	-6,37	3,99E-08
ABI3BP	ABI family, member 3 (NESH) binding protein	-6,71	1,38E-05

Supplemental Table 3. List of genes down-regulated (green) and up-regulated (red) in hiPS-NSCs with respect to hiPSCs (Absolute fold change > 1,5; False discovery rate < 0,05)

Gene symbol	Gene Name	Fold change	False discovery rate
LIN28A	lin-28 homolog A (C. elegans)	-5,73	2,64E-04
CLDN6	claudin 6	-5,54	4,87E-04
TDGF1	teratocarcinoma-derived growth factor 1	-5,26	3,81E-02
EPCAM	epithelial cell adhesion molecule	-5,23	7,18E-03
ANXA3	annexin A3	-5,06	2,61E-04
NANOG	Nanog homeobox	-5,01	1,95E-02
LIN28A	lin-28 homolog A (C. elegans)	-4,96	1,05E-03
ANKRD1	ankyrin repeat domain 1 (cardiac muscle)	-4,92	9,14E-03
LIN28B	lin-28 homolog B (C. elegans)	-4,71	1,69E-04
CST1	cystatin SN	-4,45	2,86E-03
ALPL	alkaline phosphatase, liver/bone/kidney	-4,45	1,74E-03
KRT8	keratin 8	-4,41	4,57E-04
TM4SF18	transmembrane 4 L six family member 18	-4,37	1,61E-02
EPCAM	epithelial cell adhesion molecule	-4,29	9,40E-03
NA	NA	-4,28	1,19E-02
POU5F1	POU class 5 homeobox 1	-4,21	1,05E-02
NANOG	Nanog homeobox	-4,07	2,78E-02
MST4	serine/threonine protein kinase MST4	-4,06	1,29E-02
MYOCD	myocardin	-3,97	2,95E-02
CYP2S1	cytochrome P450, family 2, subfamily S, polypeptide 1	-3,95	2,53E-03
ACTC1	actin, alpha, cardiac muscle 1	-3,92	6,41E-03
MFAP5	microfibrillar associated protein 5	-3,86	3,99E-02
DSP	desmoplakin	-3,83	5,21E-04
VAMP8	vesicle-associated membrane protein 8 (endobrevin)	-3,82	4,80E-05
NA	NA	-3,81	2,72E-02
BAIAP2L1	BAI1-associated protein 2-like 1	-3,80	1,50E-03
DSG2	desmoglein 2	-3,79	1,31E-02
GAL	galanin prepropeptide	-3,79	6,39E-04
IGF2BP1	insulin-like growth factor 2 mRNA binding protein 1	-3,78	6,23E-03
IL1A	interleukin 1, alpha	-3,69	2,11E-02
RGS5	regulator of G-protein signaling 5	-3,67	1,31E-02
USP44	ubiquitin specific peptidase 44	-3,67	3,11E-02
FAM71F1	family with sequence similarity 71, member F1	-3,66	1,35E-02
F2RL1	coagulation factor II (thrombin) receptor-like 1	-3,62	6,23E-03
FLT1	fms-related tyrosine kinase 1 (vascular endothelial growth factor/vascular permeability factor receptor)	-3,60	4,38E-03
EDN1	endothelin 1	-3,58	3,62E-03
POU5F1B	POU class 5 homeobox 1B	-3,58	2,76E-02
TRIML2	tripartite motif family-like 2	-3,53	4,03E-03
DSC2	desmocollin 2	-3,53	2,23E-03
OCIAD2	OCIA domain containing 2	-3,52	3,98E-03
SERPINB9	serpin peptidase inhibitor, clade B (ovalbumin), member 9	-3,45	1,56E-02
ENPP1	ectonucleotide pyrophosphatase/phosphodiesterase 1	-3,45	6,72E-03
PKP2	plakophilin 2	-3,34	1,20E-03
PLAC8	placenta-specific 8	-3,31	3,17E-02
KRT18	keratin 18	-3,25	1,50E-03
KRT19	keratin 19	-3,24	1,65E-03
GCNT4	glucosaminyl (N-acetyl) transferase 4, core 2	-3,23	3,60E-02
NA	NA	-3,20	3,81E-02
SALL4	sal-like 4 (Drosophila)	-3,20	4,62E-03
TAGLN	transgelin	-3,16	1,31E-02
NRK	Nik related kinase	-3,09	2,29E-02
CLDN7	claudin 7	-3,08	4,07E-02
PNP	purine nucleoside phosphorylase	-3,06	9,12E-03
ZNF114	zinc finger protein 114	-3,03	1,09E-02
BNC1	basonuclin 1	-3,03	4,21E-02
NA	NA	-3,02	3,75E-03
PAPPA	pregnancy-associated plasma protein A, pappalysin 1	-2,99	1,39E-02
RAB3B	RAB3B, member RAS oncogene family	-2,98	8,05E-03
LEPREL1	leprecan-like 1	-2,94	7,41E-03
KRT18	keratin 18	-2,90	1,78E-03
KRT18	keratin 18	-2,86	2,53E-03
F11R	F11 receptor	-2,86	5,13E-03
PLS1	plastin 1	-2,83	1,44E-03
FAM83B	family with sequence similarity 83, member B	-2,81	9,12E-03
MARVELD2	MARVEL domain containing 2	-2,79	4,73E-02
FXYD5	FXYD domain containing ion transport regulator 5	-2,77	6,39E-03
PODXL	podocalyxin-like	-2,75	2,23E-03
MAMDC2	MAM domain containing 2	-2,74	4,82E-02
ITGA2	integrin, alpha 2 (CD49B, alpha 2 subunit of VLA-2 receptor)	-2,74	2,72E-02
DPPA4	developmental pluripotency associated 4	-2,73	3,45E-02
NR6A1	nuclear receptor subfamily 6, group A, member 1	-2,72	1,37E-02
ARID3B	AT rich interactive domain 3B (BRIGHT-like)	-2,70	3,77E-03
CNN1	calponin 1, basic, smooth muscle	-2,66	3,18E-03
RBPMS2	RNA binding protein with multiple splicing 2	-2,64	1,40E-02
SPINT1	serine peptidase inhibitor, Kunitz type 1	-2,62	4,23E-02
FST	follistatin	-2,61	2,32E-02
UGT3A2	UDP glycosyltransferase 3 family, polypeptide A2	-2,59	1,44E-02
TFAP2C	transcription factor AP-2 gamma (activating enhancer binding protein 2 gamma)	-2,59	1,72E-02
NA	NA	-2,59	1,97E-02
TRIM71	tripartite motif-containing 71	-2,57	2,25E-02
PMAIP1	phorbol-12-myristate-13-acetate-induced protein 1	-2,54	2,30E-03
EMB	embigin	-2,53	8,43E-03
OCLN	occludin	-2,52	2,56E-02
LAMC2	laminin, gamma 2	-2,52	1,20E-03
MET	met proto-oncogene (hepatocyte growth factor receptor)	-2,48	3,47E-02
MICB	MHC class I polypeptide-related sequence B	-2,46	7,38E-03
LYN	v-yes-1 Yamaguchi sarcoma viral related oncogene homolog	-2,45	2,51E-02
SLC7A5	solute carrier family 7 (cationic amino acid transporter, y+ system), member 5	-2,45	3,72E-02
PLAU	plasminogen activator, urokinase	-2,44	4,82E-02
JUP	junction plakoglobin	-2,42	8,18E-04
USP53	ubiquitin specific peptidase 53	-2,40	2,19E-03
ZNF215	zinc finger protein 215	-2,38	1,30E-02
CORO2A	coronin, actin binding protein, 2A	-2,37	3,80E-02
GATA3	GATA binding protein 3	-2,37	4,36E-02
OLFML3	olfactomedin-like 3	-2,37	3,18E-03
CD177	CD177 molecule	-2,36	3,85E-02
SPINT2	serine peptidase inhibitor, Kunitz type, 2	-2,35	8,74E-03
PDLIM1	PDZ and LIM domain 1	-2,34	1,27E-02
TAF4B	TAF4b RNA polymerase II, TATA box binding protein (TBP)-associated factor, 105kDa	-2,34	3,58E-02
NA	NA	-2,33	1,24E-03
NA	NA	-2,32	1,52E-02
CARD11	caspase recruitment domain family, member 11	-2,31	2,96E-03
C14orf115	chromosome 14 open reading frame 115	-2,28	4,07E-02
HRASLS5	HRAS-like suppressor family, member 5	-2,28	4,78E-02
HELLS	helicase, lymphoid-specific	-2,27	8,89E-03
FGD6	FYVE, RhoGEF and PH domain containing 6	-2,27	1,68E-03
MYL9	myosin, light chain 9, regulatory	-2,26	4,03E-03
EBI3	Epstein-Barr virus induced 3	-2,26	4,75E-02
C21orf105	chromosome 21 open reading frame 105	-2,26	6,70E-03
KDR	kinase insert domain receptor (a type III receptor tyrosine kinase)	-2,26	1,04E-02
ACTA1	actin, alpha 1, skeletal muscle	-2,25	2,54E-02
C3orf64	chromosome 3 open reading frame 64	-2,25	6,80E-03
CYB5R2	cytochrome b5 reductase 2	-2,24	3,77E-03
CRABP2	cellular retinoic acid binding protein 2	-2,24	3,02E-02
PHACTR2	phosphatase and actin regulator 2	-2,22	9,11E-04
CNN2	calponin 2	-2,22	3,42E-04
SFN	stratifin	-2,22	3,85E-02
MTHFD2	methylenetetrahydrofolate dehydrogenase (NADP+ dependent) 2, methenyltetrahydrofolate cyclohydrolase	-2,21	2,94E-03
NUAK2	NUAK family, SNF1-like kinase, 2	-2,21	6,39E-04
APOBEC3B	apolipoprotein B mRNA editing enzyme, catalytic polypeptide-like 3B	-2,20	1,76E-02
HMGA1	high mobility group AT-hook 1	-2,20	1,08E-03
STC2	stanniocalcin 2	-2,18	1,82E-02
IL4R	interleukin 4 receptor	-2,18	8,04E-03
DIAPH3	diaphanous homolog 3 (Drosophila)	-2,17	9,61E-05
FERMT1	fermitin family member 1	-2,16	3,08E-03

STX3	syntaxin 3	-2,14	2,68E-03
SLC35F2	solute carrier family 35, member F2	-2,13	1,33E-02
ARRB1	arrestin, beta 1	-2,12	1,81E-02
MME	membrane metallo-endopeptidase	-2,09	4,54E-02
STIL	SCL/TAL1 interrupting locus	-2,09	6,43E-04
LCP1	lymphocyte cytosolic protein 1 (L-plastin)	-2,09	3,40E-02
PAWR	PRKC, apoptosis, WT1, regulator	-2,08	1,21E-02
SLC38A5	solute carrier family 38, member 5	-2,08	3,68E-03
ARHGEF5	Rho guanine nucleotide exchange factor (GEF) 5	-2,08	1,90E-02
FAR2	fatty acyl CoA reductase 2	-2,08	1,99E-02
RAP1GAP2	RAP1 GTPase activating protein 2	-2,08	1,06E-02
GPR143	G protein-coupled receptor 143	-2,07	2,20E-02
DMKN	dermokine	-2,06	2,88E-02
AURKA	aurora kinase A	-2,06	2,61E-04
SNORD25	small nucleolar RNA, C/D box 25	-2,06	1,99E-02
MYO1D	myosin ID	-2,06	3,04E-02
CCDC88C	coiled-coil domain containing 88C	-2,05	7,98E-03
C3orf59	chromosome 3 open reading frame 59	-2,05	4,65E-03
FAM60A	family with sequence similarity 60, member A	-2,05	4,56E-03
HMMR	hyaluronan-mediated motility receptor (RHAMM)	-2,04	8,99E-04
SOX15	SRY (sex determining region Y)-box 15	-2,03	9,49E-03
UGCG	UDP-glucose ceramide glucosyltransferase	-2,03	1,02E-02
DEPDC1	DEP domain containing 1	-2,03	8,78E-04
LSR	lipolysis stimulated lipoprotein receptor	-2,03	1,75E-02
TTK	TTK protein kinase	-2,02	5,15E-03
EPHA2	EPH receptor A2	-2,01	3,65E-02
MYH9	myosin, heavy chain 9, non-muscle	-2,00	5,42E-03
NFE2L3	nuclear factor (erythroid-derived 2)-like 3	-1,99	1,52E-02
NA	NA	-1,99	2,67E-03
TMEM40	transmembrane protein 40	-1,99	4,64E-02
TPM1	tropomyosin 1 (alpha)	-1,99	1,38E-02
MRS2	MRS2 magnesium homeostasis factor homolog (S. cerevisiae)	-1,98	6,39E-04
ESRP2	epithelial splicing regulatory protein 2	-1,96	3,85E-02
GFPT2	glutamine-fructose-6-phosphate transaminase 2	-1,94	1,54E-02
LRRN4	leucine rich repeat neuronal 4	-1,94	2,03E-02
ALPK3	alpha-kinase 3	-1,93	5,45E-03
FGF16	fibroblast growth factor 16	-1,93	2,65E-02
STAC	SH3 and cysteine rich domain	-1,92	3,80E-02
RAC2	ras-related C3 botulinum toxin substrate 2 (rho family, small GTP binding protein Rac2)	-1,91	1,64E-02
EXPH5	exophilin 5	-1,91	4,09E-02
RAD51	RAD51 homolog (RecA homolog, E. coli) (S. cerevisiae)	-1,91	6,36E-03
SLC38A4	solute carrier family 38, member 4	-1,91	5,96E-03
RPS6KA1	ribosomal protein S6 kinase, 90kDa, polypeptide 1	-1,91	8,87E-03
OR2A9P	olfactory receptor, family 2, subfamily A, member 9 pseudogene	-1,90	1,66E-02
SCARNA9L	small Cajal body-specific RNA 9-like (retrotransposed)	-1,90	3,01E-02
ELL2	elongation factor, RNA polymerase II, 2	-1,90	6,50E-03
CLSPN	claspin	-1,90	3,95E-02
ORC1L	origin recognition complex, subunit 1-like (S. cerevisiae)	-1,89	1,20E-02
TNFRSF10A	tumor necrosis factor receptor superfamily, member 10a	-1,88	1,88E-02
NA	NA	-1,87	1,20E-03
NA	NA	-1,87	3,49E-03
CDC6	cell division cycle 6 homolog (S. cerevisiae)	-1,87	4,82E-02
NA	NA	-1,86	3,68E-02
SLC2A1	solute carrier family 2 (facilitated glucose transporter), member 1	-1,86	5,71E-03
TES	testis derived transcript (3 LIM domains)	-1,86	2,46E-02
NA	NA	-1,86	1,02E-02
RHOF	ras homolog gene family, member F (in filopodia)	-1,86	9,11E-04
CDC20	cell division cycle 20 homolog (S. cerevisiae)	-1,85	1,24E-03
LOC100288413	similar to Uncharacterized protein LP9056	-1,84	2,47E-02
C11orf82	chromosome 11 open reading frame 82	-1,84	2,45E-02
RAB11FIP1	RAB11 family interacting protein 1 (class I)	-1,84	2,59E-03
MTHFD2	methylenetetrahydrofolate dehydrogenase (NADP+ dependent) 2, methenyltetrahydrofolate cyclohydrolase	-1,83	4,09E-03
DSC3	desmocollin 3	-1,83	1,27E-02
C6orf211	chromosome 6 open reading frame 211	-1,83	6,41E-03
BUB1B	budding uninhibited by benzimidazoles 1 homolog beta (yeast)	-1,83	1,18E-03
WDHD1	WD repeat and HMG-box DNA binding protein 1	-1,82	4,55E-02
OSBPL10	oxysterol binding protein-like 10	-1,81	1,31E-02
HMGA2	high mobility group AT-hook 2	-1,80	2,74E-02
CCRN4L	CCR4 carbon catabolite repression 4-like (S. cerevisiae)	-1,80	2,31E-03
ABI3	ABI family, member 3	-1,79	2,47E-02
PERP	PERP, TP53 apoptosis effector	-1,78	3,88E-02
TEAD4	TEA domain family member 4	-1,78	6,14E-03
TJP2	tight junction protein 2 (zona occludens 2)	-1,77	1,82E-02
TUBA1C	tubulin, alpha 1c	-1,77	1,50E-03
SLC39A14	solute carrier family 39 (zinc transporter), member 14	-1,77	1,89E-03
PLBD1	phospholipase B domain containing 1	-1,76	1,70E-02
SKA1	spindle and kinetochore associated complex subunit 1	-1,76	1,44E-03
NUDT15	nudix (nucleoside diphosphate linked moiety X)-type motif 15	-1,75	1,45E-02
TRIP13	thyroid hormone receptor interactor 13	-1,75	1,08E-03
LYPLA1	lysophospholipase I	-1,75	2,32E-03
NA	NA	-1,73	2,00E-02
SNORD22	small nucleolar RNA, C/D box 22	-1,73	2,45E-02
C18orf54	chromosome 18 open reading frame 54	-1,73	8,68E-03
TRIM59	tripartite motif-containing 59	-1,73	9,02E-03
SLC1A5	solute carrier family 1 (neutral amino acid transporter), member 5	-1,73	1,18E-02
TK1	thymidine kinase 1, soluble	-1,72	6,44E-03
NET1	neuroepithelial cell transforming 1	-1,72	1,53E-02
RBPMS	RNA binding protein with multiple splicing	-1,71	9,64E-03
CD2AP	CD2-associated protein	-1,71	2,63E-03
PLCB2	phospholipase C, beta 2	-1,71	3,77E-02
MYBL2	v-myb myeloblastosis viral oncogene homolog (avian)-like 2	-1,71	4,96E-03
MATN3	matrilin 3	-1,71	2,24E-02
SNORD27	small nucleolar RNA, C/D box 27	-1,70	1,53E-02
CNKSR1	connector enhancer of kinase suppressor of Ras 1	-1,69	1,12E-02
SLC17A9	solute carrier family 17, member 9	-1,69	3,98E-03
NA	NA	-1,69	3,04E-02
SPAG5	sperm associated antigen 5	-1,69	6,80E-03
SLC6A6	solute carrier family 6 (neurotransmitter transporter, taurine), member 6	-1,69	5,81E-03
SKA3	spindle and kinetochore associated complex subunit 3	-1,68	2,88E-03
C6orf115	chromosome 6 open reading frame 115	-1,68	6,14E-03
ETS2	v-ets erythroblastosis virus E26 oncogene homolog 2 (avian)	-1,68	2,00E-02
SNORD28	small nucleolar RNA, C/D box 28	-1,67	1,16E-02
CDCA2	cell division cycle associated 2	-1,67	1,50E-03
NA	NA	-1,67	2,50E-02
PRKCH	protein kinase C, eta	-1,67	1,56E-02
NA	NA	-1,67	5,48E-03
PLK1	polo-like kinase 1	-1,67	1,74E-03
SH3RF2	SH3 domain containing ring finger 2	-1,67	1,73E-02
DOCK2	dedicator of cytokinesis 2	-1,67	1,73E-03
C3orf52	chromosome 3 open reading frame 52	-1,67	1,63E-02
BRIP1	BRCA1 interacting protein C-terminal helicase 1	-1,66	3,48E-02
SLC7A7	solute carrier family 7 (cationic amino acid transporter, y+ system), member 7	-1,66	3,59E-02
NA	NA	-1,65	3,39E-02
HIST1H2AJ	histone cluster 1, H2aj	-1,65	3,84E-02
HTR1D	5-hydroxytryptamine (serotonin) receptor 1D	-1,65	8,62E-03
FAM72D	family with sequence similarity 72, member D	-1,65	3,61E-02
AXL	AXL receptor tyrosine kinase	-1,63	8,92E-03
GPR176	G protein-coupled receptor 176	-1,63	2,97E-02
MAD2L1	MAD2 mitotic arrest deficient-like 1 (yeast)	-1,63	2,07E-03
POU5F1P4	POU class 5 homeobox 1 pseudogene 4	-1,63	3,35E-02
TBC1D4	TBC1 domain family, member 4	-1,63	6,99E-03
SLFN13	schlafen family member 13	-1,63	3,35E-02
FANCD2	Fanconi anemia, complementation group D2	-1,63	1,03E-02
SNORD26	small nucleolar RNA, C/D box 26	-1,62	1,67E-02
PLIN2	perilipin 2	-1,62	1,18E-02
DNA2	DNA replication helicase 2 homolog (yeast)	-1,62	9,75E-03
SNORD31	small nucleolar RNA, C/D box 31	-1,62	1,66E-02
PDE6G	phosphodiesterase 6G, cGMP-specific, rod, gamma	-1,61	1,39E-02

NA	NA	-1,61	3,90E-02
BUB1	budding uninhibited by benzimidazoles 1 homolog (yeast)	-1,61	6,47E-03
NANOGP1	Nanog homeobox pseudogene 1	-1,61	4,55E-02
SMS	spermine synthase	-1,60	1,39E-02
CDC25A	cell division cycle 25 homolog A (S. pombe)	-1,60	1,44E-02
C12orf48	chromosome 12 open reading frame 48	-1,60	1,31E-02
KIF23	kinesin family member 23	-1,60	2,64E-03
RFWD3	ring finger and WD repeat domain 3	-1,60	3,70E-03
PHYHD1	phytanoyl-CoA dioxygenase domain containing 1	-1,59	2,27E-03
WWC2	WW and C2 domain containing 2	-1,59	2,11E-03
CAPG	capping protein (actin filament), gelsolin-like	-1,59	4,14E-03
C6orf150	chromosome 6 open reading frame 150	-1,59	5,45E-03
FLNB	filamin B, beta	-1,59	8,12E-03
PHF17	PHD finger protein 17	-1,59	2,16E-02
NA	NA	-1,59	4,70E-02
SNORA71C	small nucleolar RNA, H/ACA box 71C	-1,59	3,54E-03
CDCA8	cell division cycle associated 8	-1,58	8,18E-04
GSG2	germ cell associated 2 (haspin)	-1,58	2,92E-03
CKS1B	CDC28 protein kinase regulatory subunit 1B	-1,58	5,26E-03
FKBP5	FK506 binding protein 5	-1,58	5,03E-03
CALCR	calcitonin receptor	-1,58	4,47E-02
NA	NA	-1,58	1,84E-02
CDCA5	cell division cycle associated 5	-1,58	4,74E-03
CHRNB1	cholinergic receptor, nicotinic, beta 1 (muscle)	-1,57	1,26E-02
KBTBD8	kelch repeat and BTB (POZ) domain containing 8	-1,57	1,40E-02
EIF4A2	eukaryotic translation initiation factor 4A2	-1,56	8,89E-03
SLC9A3R1	solute carrier family 9 (sodium/hydrogen exchanger), member 3 regulator 1	-1,56	1,40E-02
NA	NA	-1,56	4,03E-02
DEPDC1B	DEP domain containing 1B	-1,56	1,33E-02
KIF20B	kinesin family member 20B	-1,55	1,56E-02
ADM	adrenomedullin	-1,55	4,70E-02
BAG2	BCL2-associated athanogene 2	-1,55	1,25E-02
PCK2	phosphoenolpyruvate carboxykinase 2 (mitochondrial)	-1,55	9,90E-03
DLGAP5	discs, large (Drosophila) homolog-associated protein 5	-1,54	1,46E-02
ARG2	arginase, type II	-1,54	1,06E-02
AURKB	aurora kinase B	-1,53	2,53E-03
CBS	cystathionine-beta-synthase	-1,53	4,52E-03
TCL1B	T-cell leukemia/lymphoma 1B	-1,53	4,13E-02
CCNA2	cyclin A2	-1,53	2,23E-03
C15orf42	chromosome 15 open reading frame 42	-1,53	1,85E-02
SLC25A24	solute carrier family 25 (mitochondrial carrier; phosphate carrier), member 24	-1,53	1,88E-03
CENPW	centromere protein W	-1,52	2,07E-03
SLC38A1	solute carrier family 38, member 1	-1,52	1,61E-02
PIK3CD	phosphoinositide-3-kinase, catalytic, delta polypeptide	-1,52	2,86E-03
LYPLA2	lysophospholipase II	-1,51	2,90E-03
FAM54A	family with sequence similarity 54, member A	-1,51	4,33E-03
GCNT1	glucosaminyl (N-acetyl) transferase 1, core 2	-1,50	2,14E-02
MND1	meiotic nuclear divisions 1 homolog (S. cerevisiae)	-1,50	1,81E-02
RAB6B	RAB6B, member RAS oncogene family	1,50	2,10E-02
BBS5	Bardet-Biedl syndrome 5	1,50	3,48E-02
TMEM59L	transmembrane protein 59-like	1,50	3,22E-02
MAML2	mastermind-like 2 (Drosophila)	1,50	1,33E-02
C1orf194	chromosome 1 open reading frame 194	1,50	1,15E-02
LOC349196	hypothetical LOC349196	1,51	2,08E-02
PCDHA4	protocadherin alpha 4	1,51	1,18E-02
KCNIP4	Kv channel interacting protein 4	1,51	4,82E-02
GLB1L	galactosidase, beta 1-like	1,51	1,00E-02
SHISA4	shisa homolog 4 (Xenopus laevis)	1,51	2,63E-03
ALPK1	alpha-kinase 1	1,52	5,15E-03
AMY2A	amylase, alpha 2A (pancreatic)	1,52	7,33E-03
SLC9A9	solute carrier family 9 (sodium/hydrogen exchanger), member 9	1,52	1,56E-02
BTN3A1	butyrophilin, subfamily 3, member A1	1,52	2,72E-02
TCTN2	tectonic family member 2	1,52	1,69E-02
ADAMTSL1	ADAMTS-like 1	1,52	3,88E-02
GEM	GTP binding protein overexpressed in skeletal muscle	1,53	2,21E-02
UBA7	ubiquitin-like modifier activating enzyme 7	1,53	2,93E-02
ACVR2A	activin A receptor, type IIA	1,53	9,16E-03
LRP4	low density lipoprotein receptor-related protein 4	1,53	6,75E-03
NA	NA	1,54	1,41E-02
B2M	beta-2-microglobulin	1,55	4,24E-02
TOX3	TOX high mobility group box family member 3	1,55	3,64E-02
LPPR2	lipid phosphate phosphatase-related protein type 2	1,55	3,05E-03
SDK1	sidekick homolog 1, cell adhesion molecule (chicken)	1,55	4,09E-03
LOC402778	CD225 family protein FLJ76511	1,55	1,37E-02
C11orf63	chromosome 11 open reading frame 63	1,55	8,72E-03
MMP19	matrix metallopeptidase 19	1,55	7,41E-04
DLX1	distal-less homeobox 1	1,56	4,70E-02
VEGFB	vascular endothelial growth factor B	1,56	1,55E-03
GSN	gelsolin	1,57	3,56E-02
PROS1	protein S (alpha)	1,57	4,75E-02
RAGE	renal tumor antigen	1,57	8,74E-03
CERCAM	cerebral endothelial cell adhesion molecule	1,58	2,32E-02
LOC100132167	hypothetical LOC100132167	1,58	2,26E-02
FZD3	frizzled homolog 3 (Drosophila)	1,58	1,20E-03
SRR	serine racemase	1,58	1,29E-02
TOX	thymocyte selection-associated high mobility group box	1,58	2,23E-02
REV3L	REV3-like, catalytic subunit of DNA polymerase zeta (yeast)	1,58	1,48E-02
GPC1	glypican 1	1,58	2,29E-02
NA	NA	1,59	2,25E-02
APLP1	amyloid beta (A4) precursor-like protein 1	1,59	3,13E-02
GSTM2	glutathione S-transferase mu 2 (muscle)	1,59	3,24E-03
C14orf132	chromosome 14 open reading frame 132	1,59	1,28E-02
LTBP3	latent transforming growth factor beta binding protein 3	1,59	5,48E-03
BTG2	BTG family, member 2	1,59	6,76E-03
FAM196A	family with sequence similarity 196, member A	1,59	2,81E-02
TCTN1	tectonic family member 1	1,59	4,88E-03
C7orf41	chromosome 7 open reading frame 41	1,60	2,92E-03
SEZ6L	seizure related 6 homolog (mouse)-like	1,60	2,37E-02
CPNE8	copine VIII	1,60	1,38E-02
ATXN1	ataxin 1	1,60	4,76E-02
C10orf107	chromosome 10 open reading frame 107	1,60	2,35E-02
LYRM5	LYR motif containing 5	1,61	6,78E-03
EFR3B	EFR3 homolog B (S. cerevisiae)	1,61	3,27E-02
PLCD4	phospholipase C, delta 4	1,61	2,36E-02
PCDH7	protocadherin 7	1,61	1,89E-02
SECTM1	secreted and transmembrane 1	1,62	1,00E-02
METTL7B	methyltransferase like 7B	1,62	5,69E-03
ANKRD6	ankyrin repeat domain 6	1,62	4,52E-03
TRIB2	tribbles homolog 2 (Drosophila)	1,62	3,46E-02
BEX1	brain expressed, X-linked 1	1,62	1,43E-02
CXCL16	chemokine (C-X-C motif) ligand 16	1,62	3,90E-02
SIRT2	sirtuin 2	1,62	7,73E-03
SYNM	synemin, intermediate filament protein	1,62	1,79E-02
ABCD2	ATP-binding cassette, sub-family D (ALD), member 2	1,64	4,23E-02
GALNTL1	UDP-N-acetyl-alpha-D-galactosamine:polypeptide N-acetylgalactosaminyltransferase-like 1	1,64	3,63E-02
ICA1L	islet cell autoantigen 1,69kDa-like	1,65	4,03E-03
GNG2	guanine nucleotide binding protein (G protein), gamma 2	1,65	1,44E-03
C2orf67	chromosome 2 open reading frame 67	1,65	1,65E-03
CADM4	cell adhesion molecule 4	1,66	3,74E-03
PMP22	peripheral myelin protein 22	1,66	2,93E-02
FAM13C	family with sequence similarity 13, member C	1,66	2,46E-02
GMPR	guanosine monophosphate reductase	1,66	1,32E-02
SOX9	SRY (sex determining region Y)-box 9	1,66	2,74E-02
TMEM8B	transmembrane protein 8B	1,66	2,09E-03
SORCS2	sortilin-related VPS10 domain containing receptor 2	1,66	1,08E-03
TET2	tet oncogene family member 2	1,66	1,90E-02
VIT	vitrin	1,66	2,08E-02
B3GAT1	beta-1,3-glucuronyltransferase 1 (glucuronosyltransferase P)	1,67	2,10E-02

B4GALNT4	beta-1,4-N-acetyl-galactosaminyl transferase 4	1,67	6,70E-03
ARHGEF26	Rho guanine nucleotide exchange factor (GEF) 26	1,68	3,50E-02
GRIA3	glutamate receptor, ionotrophic, AMPA 3	1,68	3,50E-02
GAS7	growth arrest-specific 7	1,69	7,45E-03
BCL6	B-cell CLL/lymphoma 6	1,69	3,94E-02
HAS2	hyaluronan synthase 2	1,69	2,74E-02
MEGF11	multiple EGF-like-domains 11	1,69	6,44E-03
MRC2	mannose receptor, C type 2	1,69	3,16E-02
EPB41L3	erythrocyte membrane protein band 4.1-like 3	1,70	2,48E-02
TTYH2	tweety homolog 2 (Drosophila)	1,70	1,44E-02
FXYD6	FXYD domain containing ion transport regulator 6	1,70	2,33E-03
SNORD115-20	small nucleolar RNA, C/D box 115-20	1,70	3,47E-02
PPAP2B	phosphatidic acid phosphatase type 2B	1,70	3,50E-02
ALDH5A1	aldehyde dehydrogenase 5 family, member A1	1,70	8,01E-03
ZNF432	zinc finger protein 432	1,70	8,60E-03
RAPGEF4	Rap guanine nucleotide exchange factor (GEF) 4	1,71	1,51E-02
KIAA1161	KIAA1161	1,71	6,66E-03
NA	NA	1,72	1,38E-02
IDUA	iduronidase, alpha-L-	1,72	1,74E-03
MAP6	microtubule-associated protein 6	1,72	7,28E-03
SDC3	syndecan 3	1,72	2,11E-03
FAM47E	family with sequence similarity 47, member E	1,72	9,88E-03
NME5	non-metastatic cells 5, protein expressed in (nucleoside-diphosphate kinase)	1,72	2,17E-02
ANGPT1	angiopoietin 1	1,73	1,39E-02
PCDHB12	protocadherin beta 12	1,73	1,18E-02
KLHDC1	kelch domain containing 1	1,73	1,24E-02
P2RX7	purinergic receptor P2X, ligand-gated ion channel, 7	1,73	1,07E-02
C5orf36	chromosome 5 open reading frame 36	1,74	1,76E-02
PREX1	phosphatidylinositol-3,4,5-trisphosphate-dependent Rac exchange factor 1	1,74	2,23E-03
C12orf53	chromosome 12 open reading frame 53	1,74	6,44E-03
GABRG3	gamma-aminobutyric acid (GABA) A receptor, gamma 3	1,74	4,37E-02
PGM5P2	phosphoglucomutase 5 pseudogene 2	1,75	4,02E-02
RUNDC3A	RUN domain containing 3A	1,75	4,58E-02
CAMTA1	calmodulin binding transcription activator 1	1,75	2,53E-03
CELSR2	cadherin, EGF LAG seven-pass G-type receptor 2 (flamingo homolog, Drosophila)	1,76	2,48E-03
NA	NA	1,76	1,92E-02
FREM1	FRAS1 related extracellular matrix 1	1,76	1,90E-02
SMARCA2	SWI/SNF related, matrix associated, actin dependent regulator of chromatin, subfamily a, member 2	1,77	5,57E-03
RNF165	ring finger protein 165	1,77	2,03E-02
ZNF704	zinc finger protein 704	1,77	3,46E-02
OPHN1	oligophrenin 1	1,78	3,17E-02
LPHN3	latrophilin 3	1,78	1,18E-02
CLU	clusterin	1,78	5,45E-03
NA	NA	1,78	1,83E-02
ANKRD44	ankyrin repeat domain 44	1,79	3,01E-03
NAALADL2	N-acetylated alpha-linked acidic dipeptidase-like 2	1,79	2,84E-03
SLC22A17	solute carrier family 22, member 17	1,79	3,70E-03
GNG3	guanine nucleotide binding protein (G protein), gamma 3	1,79	4,34E-02
EFHC2	EF-hand domain (C-terminal) containing 2	1,80	1,99E-02
C10orf90	chromosome 10 open reading frame 90	1,81	4,12E-02
PCDHB13	protocadherin beta 13	1,81	1,48E-02
KREMEN1	kringle containing transmembrane protein 1	1,81	2,11E-03
LRRC4B	leucine rich repeat containing 4B	1,82	2,26E-03
ANKS1B	ankyrin repeat and sterile alpha motif domain containing 1B	1,83	3,03E-03
GUCY1B3	guanylate cyclase 1, soluble, beta 3	1,83	4,12E-02
DKK3	dickkopf homolog 3 (Xenopus laevis)	1,83	3,48E-02
SESN3	sestrin 3	1,83	2,92E-03
ARHGAP24	Rho GTPase activating protein 24	1,83	1,74E-03
NRXN3	neurexin 3	1,84	3,15E-02
CHST3	carbohydrate (chondroitin 6) sulfotransferase 3	1,84	1,60E-02
DNAJC12	DnaJ (Hsp40) homolog, subfamily C, member 12	1,84	4,80E-03
CACNB4	calcium channel, voltage-dependent, beta 4 subunit	1,84	7,84E-03
RUFY3	RUN and FYVE domain containing 3	1,84	1,50E-03
CRYL1	crystallin, lambda 1	1,84	4,83E-03
ID4	inhibitor of DNA binding 4, dominant negative helix-loop-helix protein	1,85	2,63E-03
ZDHHC1	zinc finger, DHHC-type containing 1	1,85	4,83E-03
ACCN2	amiloride-sensitive cation channel 2, neuronal	1,85	3,74E-02
CCDC30	coiled-coil domain containing 30	1,85	2,53E-03
NA	NA	1,85	8,99E-04
SNORD115-33	small nucleolar RNA, C/D box 115-33	1,86	2,96E-02
DOK5	docking protein 5	1,86	3,92E-02
LOC349196	hypothetical LOC349196	1,87	2,56E-02
ZNF521	zinc finger protein 521	1,87	1,40E-02
PYGO1	pygopus homolog 1 (Drosophila)	1,88	2,04E-02
ZCCHC12	zinc finger, CCHC domain containing 12	1,88	3,73E-02
IFIT2	interferon-induced protein with tetratricopeptide repeats 2	1,89	4,41E-02
FAM171B	family with sequence similarity 171, member B	1,89	4,09E-03
SPATA6	spermatogenesis associated 6	1,89	7,00E-03
RDH10	retinol dehydrogenase 10 (all-trans)	1,90	3,17E-02
SIAE	sialic acid acetylesterase	1,90	4,50E-03
NA	NA	1,90	9,92E-03
FGFBP2	fibroblast growth factor binding protein 2	1,90	4,97E-02
THSD7A	thrombospondin, type I, domain containing 7A	1,90	3,19E-02
PACRG	PARK2 co-regulated	1,90	5,50E-03
MASP1	mannan-binding lectin serine peptidase 1 (C4/C2 activating component of Ra-reactive factor)	1,91	1,16E-03
ANKRD45	ankyrin repeat domain 45	1,91	4,83E-03
C5orf4	chromosome 5 open reading frame 4	1,91	1,39E-02
C3orf70	chromosome 3 open reading frame 70	1,91	7,88E-03
CCDC146	coiled-coil domain containing 146	1,91	1,34E-02
NA	NA	1,91	6,39E-04
ASTN1	astrotactin 1	1,92	1,37E-02
THRA	thyroid hormone receptor, alpha (erythroblastic leukemia viral (v-erb-a) oncogene homolog, avian)	1,92	2,23E-03
C5	complement component 5	1,92	1,73E-03
ERV3	endogenous retroviral sequence 3	1,92	3,70E-03
NA	NA	1,93	8,80E-03
UNC80	unc-80 homolog (C. elegans)	1,93	1,26E-03
HCFC2	host cell factor C2	1,93	2,27E-02
LONRF2	LON peptidase N-terminal domain and ring finger 2	1,94	5,13E-03
C1S	complement component 1, s subcomponent	1,94	1,20E-03
SEMA5A	sema domain, seven thrombospondin repeats (type 1 and type 1-like), transmembrane domain (TM) and short cytoplasmic domain, (semaphorin) 5A	1,95	8,49E-03
PGM5P2	phosphoglucomutase 5 pseudogene 2	1,95	2,54E-02
PLEKHB1	pleckstrin homology domain containing, family B (evectins) member 1	1,95	4,46E-02
FOXB1	forkhead box B1	1,95	2,31E-02
SOBP	sine oculis binding protein homolog (Drosophila)	1,96	3,77E-03
LRP1B	low density lipoprotein receptor-related protein 1B	1,96	2,57E-02
ABCA2	ATP-binding cassette, sub-family A (ABC1), member 2	1,96	9,28E-04
B4GALNT1	beta-1,4-N-acetyl-galactosaminyl transferase 1	1,96	4,80E-03
SLC4A10	solute carrier family 4, sodium bicarbonate transporter, member 10	1,96	6,23E-03
FEZ1	fasciculation and elongation protein zeta 1 (zygin I)	1,97	1,48E-03
DLL1	delta-like 1 (Drosophila)	1,98	1,65E-02
PDE4B	phosphodiesterase 4B, cAMP-specific	1,98	8,31E-03
SLAIN1	SLAIN motif family, member 1	1,99	8,80E-03
KCNN3	potassium intermediate/small conductance calcium-activated channel, subfamily N, member 3	2,00	1,71E-02
TRIL	TLR4 interactor with leucine rich repeats	2,01	1,89E-03
CRB1	crumbs homolog 1 (Drosophila)	2,01	8,62E-04
FAM5B	family with sequence similarity 5, member B	2,02	2,25E-02
BMPR1B	bone morphogenetic protein receptor, type IB	2,02	1,65E-02
SYBU	syntabulin (syntaxin-interacting)	2,03	1,40E-02
POU3F2	POU class 3 homeobox 2	2,03	1,56E-02
GAB1	GRB2-associated binding protein 1	2,04	6,39E-04
SYT17	synaptotagmin XVII	2,04	1,27E-02
ALK	anaplastic lymphoma receptor tyrosine kinase	2,05	2,23E-02
TNR	tenascin R (restrictin, janusin)	2,05	2,55E-02
MDGA2	MAM domain containing glycosylphosphatidylinositol anchor 2	2,05	3,29E-02
GNG7	guanine nucleotide binding protein (G protein), gamma 7	2,05	1,19E-02
ATP9A	ATPase, class II, type 9A	2,05	1,44E-03
SNCAIP	synuclein, alpha interacting protein	2,05	4,37E-03
NA	NA	2,06	6,39E-04

MAPRE3	microtubule-associated protein, RP/EB family, member 3	2,06	1,22E-02
MGAT4A	mannosyl (alpha-1,3-)-glycoprotein beta-1,4-N-acetylglucosaminyltransferase, isozyme A	2,06	3,91E-02
PAK3	p21 protein (Cdc42/Rac)-activated kinase 3	2,06	3,81E-03
TPPP3	tubulin polymerization-promoting protein family member 3	2,07	8,81E-03
EPHA5	EPH receptor A5	2,07	2,42E-02
TXNIP	thioredoxin interacting protein	2,07	4,15E-02
LIFR	leukemia inhibitory factor receptor alpha	2,08	3,44E-03
SLITRK1	SLIT and NTRK-like family, member 1	2,08	8,93E-03
EYA1	eyes absent homolog 1 (Drosophila)	2,08	4,31E-03
PCDHB11	protocadherin beta 11	2,09	1,44E-03
DNALI1	dynein, axonemal, light intermediate chain 1	2,10	2,92E-03
STK32A	serine/threonine kinase 32A	2,10	1,08E-02
HS3ST3B1	heparan sulfate (glucosamine) 3-O-sulfotransferase 3B1	2,10	1,11E-02
PROX1	prospero homeobox 1	2,10	4,37E-03
GRID1	glutamate receptor, ionotropic, delta 1	2,10	6,39E-04
CCDC102B	coiled-coil domain containing 102B	2,11	7,05E-03
BAI2	brain-specific angiogenesis inhibitor 2	2,11	8,99E-04
PCDHB2	protocadherin beta 2	2,12	1,23E-03
ATP6V1G2	ATPase, H+ transporting, lysosomal 13kDa, V1 subunit G2	2,12	7,67E-03
PDLIM3	PDZ and LIM domain 3	2,12	1,47E-02
PLA2G4A	phospholipase A2, group IVA (cytosolic, calcium-dependent)	2,13	8,87E-03
REEP1	receptor accessory protein 1	2,13	3,77E-02
GPR162	G protein-coupled receptor 162	2,14	3,70E-03
NUPR1	nuclear protein, transcriptional regulator, 1	2,14	1,37E-02
CPNE4	copine IV	2,15	2,72E-02
PRKD1	protein kinase D1	2,15	2,54E-02
BTN3A3	butyrophilin, subfamily 3, member A3	2,15	3,01E-03
MTTP	microsomal triglyceride transfer protein	2,16	4,37E-02
GPM6B	glycoprotein M6B	2,16	2,18E-02
CACNG7	calcium channel, voltage-dependent, gamma subunit 7	2,16	1,16E-02
CACNB2	calcium channel, voltage-dependent, beta 2 subunit	2,17	1,39E-02
SCN2A	sodium channel, voltage-gated, type II, alpha subunit	2,17	1,40E-02
IFI16	interferon, gamma-inducible protein 16	2,17	3,20E-02
GPR17	G protein-coupled receptor 17	2,18	4,36E-02
ARAP2	ArfGAP with RhoGAP domain, ankyrin repeat and PH domain 2	2,19	4,80E-03
ARNT2	aryl-hydrocarbon receptor nuclear translocator 2	2,19	2,93E-02
CTSO	cathepsin O	2,20	2,82E-02
MAPK4	mitogen-activated protein kinase 4	2,20	3,92E-02
ANK2	ankyrin 2, neuronal	2,20	3,70E-03
SLC35F1	solute carrier family 35, member F1	2,21	1,02E-02
LSAMP	limbic system-associated membrane protein	2,21	6,80E-03
TSPAN11	tetraspanin 11	2,21	4,78E-03
UNC80	unc-80 homolog (C. elegans)	2,22	1,69E-04
ITPKB	inositol 1,4,5-trisphosphate 3-kinase B	2,23	5,67E-03
RTN1	reticulon 1	2,23	2,93E-02
PDZRN3	PDZ domain containing ring finger 3	2,23	2,37E-02
TMEM232	transmembrane protein 232	2,23	2,68E-03
LOC100132167	hypothetical LOC100132167	2,23	1,23E-02
WSCD1	WSC domain containing 1	2,23	2,00E-02
ABCA1	ATP-binding cassette, sub-family A (ABC1), member 1	2,24	5,63E-03
KIF5A	kinesin family member 5A	2,24	2,57E-02
MANEAL	mannosidase, endo-alpha-like	2,25	1,28E-03
MMRN1	multimerin 1	2,26	4,70E-02
AKR1C1	aldo-keto reductase family 1, member C1 (dihydrodiol dehydrogenase 1; 20-alpha (3-alpha)-hydroxysteroid dehydrogenase)	2,26	3,14E-03
NA	NA	2,27	2,67E-02
SOX5	SRY (sex determining region Y)-box 5	2,27	1,44E-02
ST8SIA1	ST8 alpha-N-acetyl-neuraminide alpha-2,8-sialyltransferase 1	2,27	3,83E-02
ADCYAP1R1	adenylate cyclase activating polypeptide 1 (pituitary) receptor type I	2,27	1,10E-02
EPHB3	EPH receptor B3	2,28	2,64E-04
ST6GALNAC5	ST6 (alpha-N-acetyl-neuraminyl-2,3-beta-galactosyl-1,3)-N-acetylgalactosaminide alpha-2,6-sialyltransferase 5	2,29	2,44E-02
SEMA5B	sema domain, seven thrombospondin repeats (type 1 and type 1-like), transmembrane domain (TM) and short cytoplasmic domain, (semaphorin) 5B	2,29	8,63E-03
ZFHX4	zinc finger homeobox 4	2,30	1,31E-02
CACNG4	calcium channel, voltage-dependent, gamma subunit 4	2,30	4,80E-03
CNTFR	ciliary neurotrophic factor receptor	2,30	8,10E-03
ZNF25	zinc finger protein 25	2,30	6,20E-03
MEGF10	multiple EGF-like-domains 10	2,31	2,15E-02
WDR17	WD repeat domain 17	2,31	1,20E-03
PCDHB15	protocadherin beta 15	2,32	1,87E-02
FAM134B	family with sequence similarity 134, member B	2,32	1,50E-02
KIF5A	kinesin family member 5A	2,32	2,07E-02
OLFML2B	olfactomedin-like 2B	2,32	3,36E-02
PPIL6	peptidylprolyl isomerase (cyclophilin)-like 6	2,34	3,49E-03
RNF157	ring finger protein 157	2,34	1,44E-03
METTL7A	methyltransferase like 7A	2,34	1,85E-02
PCDHB14	protocadherin beta 14	2,34	1,19E-02
SORCS1	sortilin-related VPS10 domain containing receptor 1	2,36	2,39E-02
ZNF436	zinc finger protein 436	2,36	2,94E-02
PAX6	paired box 6	2,37	7,68E-03
GPR137B	G protein-coupled receptor 137B	2,37	5,03E-03
PLP1	proteolipid protein 1	2,38	1,34E-02
C1R	complement component 1, r subcomponent	2,38	1,18E-02
NTRK3	neurotrophic tyrosine kinase, receptor, type 3	2,38	3,03E-03
ZBTB20	zinc finger and BTB domain containing 20	2,38	3,48E-02
LYST	lysosomal trafficking regulator	2,39	1,96E-02
TMOD2	tropomodulin 2 (neuronal)	2,39	3,49E-03
NA	NA	2,39	2,25E-02
CDH10	cadherin 10, type 2 (T2-cadherin)	2,39	2,68E-02
DPYD	dihydropyrimidine dehydrogenase	2,39	1,88E-02
NCALD	neurocalcin delta	2,40	3,49E-03
PLSCR4	phospholipid scramblase 4	2,40	4,33E-03
NCKAP5	NCK-associated protein 5	2,41	2,99E-02
TRIM9	tripartite motif-containing 9	2,41	2,07E-03
GDPD2	glycerophosphodiester phosphodiesterase domain containing 2	2,42	2,45E-02
HOXA2	homeobox A2	2,42	2,25E-02
PAR4	Prader-Willi/Angelman region gene 4	2,42	4,78E-02
SNAP25	synaptosomal-associated protein, 25kDa	2,42	6,43E-03
PCDHB3	protocadherin beta 3	2,43	8,62E-03
SSPN	sarcospan (Kras oncogene-associated gene)	2,43	7,88E-03
TMEFF2	transmembrane protein with EGF-like and two follistatin-like domains 2	2,43	1,53E-02
RNF180	ring finger protein 180	2,44	1,92E-02
PCDHB18	protocadherin beta 18 pseudogene	2,44	4,90E-02
PPFIA2	protein tyrosine phosphatase, receptor type, f polypeptide (PTPRF), interacting protein (liprin), alpha 2	2,44	4,73E-03
LOC349196	hypothetical LOC349196	2,44	1,03E-02
KCNA2	potassium voltage-gated channel, shaker-related subfamily, member 2	2,45	9,70E-03
CPNE5	copine V	2,45	4,52E-03
PLEKHG1	pleckstrin homology domain containing, family G (with RhoGef domain) member 1	2,45	1,39E-02
NA	NA	2,46	2,92E-02
IGSF11	immunoglobulin superfamily, member 11	2,46	7,41E-04
FGF14	fibroblast growth factor 14	2,49	3,80E-02
GAP43	growth associated protein 43	2,51	3,94E-02
SNORD115-44	small nucleolar RNA, C/D box 115-44	2,51	4,54E-02
TSPAN11	tetraspanin 11	2,52	6,26E-03
LGALS3	lectin, galactoside-binding, soluble, 3	2,52	2,11E-03
NFIB	nuclear factor I/B	2,53	2,63E-03
AKR1C2	aldo-keto reductase family 1, member C2 (dihydrodiol dehydrogenase 2; bile acid binding protein; 3-alpha hydroxysteroid dehydrogenase, type III)	2,53	6,72E-03
GPR158	G protein-coupled receptor 158	2,53	3,20E-02
NA	NA	2,55	6,40E-03
SLC40A1	solute carrier family 40 (iron-regulated transporter), member 1	2,55	1,89E-03
PDE7B	phosphodiesterase 7B	2,55	4,16E-03
C10orf72	chromosome 10 open reading frame 72	2,55	6,38E-03
WDR49	WD repeat domain 49	2,55	3,24E-03
SMOC1	SPARC related modular calcium binding 1	2,55	1,52E-02
GATM	glycine amidinotransferase (L-arginine:glycine amidinotransferase)	2,56	1,73E-02
IFI44	interferon-induced protein 44	2,56	9,90E-03
FOXP2	forkhead box P2	2,56	1,11E-02
PCDHB4	protocadherin beta 4	2,56	8,62E-03
GLT25D2	glycosyltransferase 25 domain containing 2	2,59	8,89E-03
MAB21L2	mab-21-like 2 (C. elegans)	2,59	6,63E-03

GNG2	guanine nucleotide binding protein (G protein), gamma 2	2,60	4,78E-03
RRAGD	Ras-related GTP binding D	2,60	1,40E-02
C20orf103	chromosome 20 open reading frame 103	2,60	2,86E-02
KCNE4	potassium voltage-gated channel, Isk-related family, member 4	2,61	1,63E-03
GRIN2B	glutamate receptor, ionotropic, N-methyl D-aspartate 2B	2,62	2,92E-03
C1QTNF3	C1q and tumor necrosis factor related protein 3	2,62	1,90E-03
MEGF9	multiple EGF-like-domains 9	2,62	5,59E-03
ARRDC4	arrestin domain containing 4	2,62	2,50E-02
EGR2	early growth response 2	2,63	2,51E-02
TMEM117	transmembrane protein 117	2,63	3,05E-03
SRGAP3	SLIT-ROBO Rho GTPase activating protein 3	2,64	5,13E-04
NPAS3	neuronal PAS domain protein 3	2,66	3,50E-03
SLC1A2	solute carrier family 1 (glial high affinity glutamate transporter), member 2	2,66	2,07E-03
RHOBTB3	Rho-related BTB domain containing 3	2,66	2,07E-02
NRCAM	neuronal cell adhesion molecule	2,67	1,17E-02
PLEKHH2	pleckstrin homology domain containing, family H (with MyTH4 domain) member 2	2,67	2,63E-03
ARHGEF6	Rac/Cdc42 guanine nucleotide exchange factor (GEF) 6	2,67	4,85E-03
FMN2	formin 2	2,67	3,36E-03
NCAM2	neural cell adhesion molecule 2	2,68	1,23E-02
RGS7BP	regulator of G-protein signaling 7 binding protein	2,68	8,17E-03
CNR1	cannabinoid receptor 1 (brain)	2,68	9,13E-03
NKAIN3	Na+/K+ transporting ATPase interacting 3	2,69	1,39E-03
SEPP1	selenoprotein P, plasma, 1	2,70	1,65E-02
MAPT	microtubule-associated protein tau	2,70	1,91E-02
SLITRK3	SLIT and NTRK-like family, member 3	2,70	1,10E-02
ZEB1	zinc finger E-box binding homeobox 1	2,71	4,34E-02
PAX3	paired box 3	2,72	1,53E-02
NOVA1	neuro-oncological ventral antigen 1	2,72	1,51E-03
JAKMIP2	janus kinase and microtubule interacting protein 2	2,73	1,78E-03
TMEM229B	transmembrane protein 229B	2,73	1,47E-02
SCD5	stearoyl-CoA desaturase 5	2,75	3,30E-03
TMTC2	transmembrane and tetratricopeptide repeat containing 2	2,75	5,91E-03
ODZ1	odz, odd Oz/ten-m homolog 1(Drosophila)	2,77	7,54E-03
ABCC9	ATP-binding cassette, sub-family C (CFTR/MRP), member 9	2,77	6,66E-03
JAM2	junctional adhesion molecule 2	2,78	5,50E-03
BHLHE41	basic helix-loop-helix family, member e41	2,79	9,12E-03
B3GALT2	UDP-Gal:betaGlcNAc beta 1,3-galactosyltransferase, polypeptide 2	2,79	4,13E-02
PDE3A	phosphodiesterase 3A, cGMP-inhibited	2,80	4,74E-03
SEPT3	septin 3	2,81	2,64E-02
APCDD1	adenomatosis polyposis coli down-regulated 1	2,81	1,21E-02
ABI3BP	ABI family, member 3 (NESH) binding protein	2,82	2,33E-02
CD302	CD302 molecule	2,82	4,31E-03
LRRC4C	leucine rich repeat containing 4C	2,85	8,99E-04
LPL	lipoprotein lipase	2,85	1,53E-02
KCND3	potassium voltage-gated channel, Shal-related subfamily, member 3	2,86	4,66E-03
MAB21L1	mab-21-like 1 (C. elegans)	2,87	2,84E-02
NLGN1	neuroligin 1	2,87	4,49E-03
GRIK3	glutamate receptor, ionotropic, kainate 3	2,88	4,02E-02
C1QL1	complement component 1, q subcomponent-like 1	2,88	4,31E-03
CTNND2	catenin (cadherin-associated protein), delta 2 (neural plakophilin-related arm-repeat protein)	2,88	7,41E-04
RFX4	regulatory factor X, 4 (influences HLA class II expression)	2,89	1,66E-02
SPOCK2	sparc/osteonectin, cwcv and kazal-like domains proteoglycan (testican) 2	2,90	6,50E-03
ST8SIA5	ST8 alpha-N-acetyl-neuraminide alpha-2,8-sialyltransferase 5	2,91	6,50E-03
SOX6	SRY (sex determining region Y)-box 6	2,92	1,88E-03
SCUBE2	signal peptide, CUB domain, EGF-like 2	2,92	6,39E-04
NDP	Norrie disease (pseudoglioma)	2,92	4,10E-03
NRN1	neuritin 1	2,92	4,52E-02
PCDHB9	protocadherin beta 9	2,92	8,78E-03
C6orf138	chromosome 6 open reading frame 138	2,92	3,53E-02
SLC38A3	solute carrier family 38, member 3	2,92	2,33E-02
LMO3	LIM domain only 3 (rhombotin-like 2)	2,95	4,52E-03
BCAN	brevican	2,95	2,53E-03
NCAM1	neural cell adhesion molecule 1	2,96	4,50E-03
ST8SIA4	ST8 alpha-N-acetyl-neuraminide alpha-2,8-sialyltransferase 4	2,96	8,76E-03
ROBO2	roundabout, axon guidance receptor, homolog 2 (Drosophila)	2,96	2,57E-03
RFTN2	raftlin family member 2	2,96	4,31E-03
GRM3	glutamate receptor, metabotropic 3	2,98	1,05E-03
LAMA2	laminin, alpha 2	2,99	1,50E-03
TAGLN3	transgelin 3	3,03	6,66E-03
ANKFN1	ankyrin-repeat and fibronectin type III domain containing 1	3,05	9,55E-03
SLC6A1	solute carrier family 6 (neurotransmitter transporter, GABA), member 1	3,06	4,21E-02
KAT2B	K(lysine) acetyltransferase 2B	3,07	4,85E-03
CYBRD1	cytochrome b reductase 1	3,07	1,65E-03
BOC	Boc homolog (mouse)	3,10	6,75E-03
APOD	apolipoprotein D	3,10	6,78E-03
SLIT1	slit homolog 1 (Drosophila)	3,11	6,39E-04
KLHDC8A	kelch domain containing 8A	3,11	3,64E-03
ABCA8	ATP-binding cassette, sub-family A (ABC1), member 8	3,13	3,85E-02
C2orf80	chromosome 2 open reading frame 80	3,14	1,58E-03
PPEF1	protein phosphatase, EF-hand calcium binding domain 1	3,16	3,20E-02
MTSS1	metastasis suppressor 1	3,16	8,99E-04
SNORD115-32	small nucleolar RNA, C/D box 115-32	3,16	2,52E-02
FRZB	frizzled-related protein	3,17	4,53E-03
CALCRL	calcitonin receptor-like	3,20	1,09E-02
PLCE1	phospholipase C, epsilon 1	3,21	1,01E-02
ATP10B	ATPase, class V, type 10B	3,21	2,63E-03
MOXD1	monooxygenase, DBH-like 1	3,22	7,32E-03
SLITRK2	SLIT and NTRK-like family, member 2	3,22	1,16E-03
ST8SIA1	ST8 alpha-N-acetyl-neuraminide alpha-2,8-sialyltransferase 1	3,23	4,74E-03
ITGB8	integrin, beta 8	3,24	1,40E-02
PRRX1	paired related homeobox 1	3,26	1,20E-02
TTYH1	tweety homolog 1 (Drosophila)	3,29	7,57E-04
SLC15A2	solute carrier family 15 (H+/peptide transporter), member 2	3,31	1,20E-02
SNTG1	syntrophin, gamma 1	3,31	1,44E-02
SNORD115-11	small nucleolar RNA, C/D box 115-11	3,33	2,80E-02
GPR155	G protein-coupled receptor 155	3,34	2,67E-03
MEIS1	Meis homeobox 1	3,36	1,39E-02
SCG2	secretogranin II	3,38	1,50E-03
KCNJ10	potassium inwardly-rectifying channel, subfamily J, member 10	3,38	7,93E-03
CDH20	cadherin 20, type 2	3,39	4,37E-03
SLC6A11	solute carrier family 6 (neurotransmitter transporter, GABA), member 11	3,39	1,27E-02
CP	ceruloplasmin (ferroxidase)	3,39	1,43E-02
NA	NA	3,39	2,78E-02
ABAT	4-aminobutyrate aminotransferase	3,40	1,90E-03
DCX	doublecortin	3,41	3,29E-02
ITGB4	integrin, beta 4	3,45	5,91E-03
EBF1	early B-cell factor 1	3,46	1,40E-02
SAMD9L	sterile alpha motif domain containing 9-like	3,49	3,01E-02
PCDHB16	protocadherin beta 16	3,49	3,91E-03
C21orf62	chromosome 21 open reading frame 62	3,50	3,85E-02
SCN3A	sodium channel, voltage-gated, type III, alpha subunit	3,50	2,63E-03
AKAP6	A kinase (PRKA) anchor protein 6	3,50	2,61E-04
BCHE	butyrylcholinesterase	3,51	6,27E-03
DCC	deleted in colorectal carcinoma	3,52	4,52E-03
TSHZ2	teashirt zinc finger homeobox 2	3,53	5,13E-04
PCDH15	protocadherin-related 15	3,53	2,67E-02
FAIM2	Fas apoptotic inhibitory molecule 2	3,54	7,93E-04
ERBB4	v-erb-a erythroblastic leukemia viral oncogene homolog 4 (avian)	3,55	4,02E-03
BAI3	brain-specific angiogenesis inhibitor 3	3,57	1,69E-04
ENKUR	enkurin, TRPC channel interacting protein	3,57	1,06E-02
FIBIN	fin bud initiation factor homolog (zebrafish)	3,58	1,05E-03
GFAP	glial fibrillary acidic protein	3,60	1,81E-02
RGS6	regulator of G-protein signaling 6	3,61	9,21E-03
GABBR2	gamma-aminobutyric acid (GABA) B receptor, 2	3,62	4,38E-03
PTN	pleiotrophin	3,63	8,99E-04
NLGN3	neuroligin 3	3,68	8,18E-04
PCDHB10	protocadherin beta 10	3,68	1,55E-03
PCDH9	protocadherin 9	3,73	8,99E-04

MAP2	microtubule-associated protein 2	3,76	1,53E-02
EPHA3	EPH receptor A3	3,78	1,56E-02
SEMA6D	sema domain, transmembrane domain (TM), and cytoplasmic domain, (semaphorin) 6D	3,78	1,22E-02
FAM70A	family with sequence similarity 70, member A	3,79	2,63E-03
C21orf34	chromosome 21 open reading frame 34	3,80	2,19E-03
GRIA1	glutamate receptor, ionotropic, AMPA 1	3,81	4,50E-03
SCN1A	sodium channel, voltage-gated, type I, alpha subunit	3,81	4,80E-03
BAALC	brain and acute leukemia, cytoplasmic	3,82	1,08E-03
NFIX	nuclear factor I/X (CCAAT-binding transcription factor)	3,83	6,39E-04
OGN	osteoglycin	3,94	4,42E-02
SLC1A3	solute carrier family 1 (glial high affinity glutamate transporter), member 3	3,96	1,26E-03
BBOX1	butyrobetaine (gamma), 2-oxoglutarate dioxygenase (gamma-butyrobetaine hydroxylase) 1	3,96	5,48E-03
CACNG5	calcium channel, voltage-dependent, gamma subunit 5	3,97	3,46E-03
MLC1	megalencephalic leukoencephalopathy with subcortical cysts 1	4,05	3,70E-03
SLCO1C1	solute carrier organic anion transporter family, member 1C1	4,06	1,92E-02
KCNIP1	Kv channel interacting protein 1	4,06	2,27E-03
LPPR4	lipid phosphate phosphatase-related protein type 4	4,11	1,51E-03
IFI44L	interferon-induced protein 44-like	4,15	1,38E-02
ZIC1	Zic family member 1 (odd-paired homolog, Drosophila)	4,16	1,83E-02
CA12	carbonic anhydrase XII	4,22	1,29E-04
RPE65	retinal pigment epithelium-specific protein 65kDa	4,23	5,26E-03
NA	NA	4,24	6,39E-04
ATP1B2	ATPase, Na+/K+ transporting, beta 2 polypeptide	4,24	1,74E-03
PI15	peptidase inhibitor 15	4,25	2,60E-02
GPR56	G protein-coupled receptor 56	4,28	3,24E-03
SYT11	synaptotagmin XI	4,30	4,76E-03
GRIA2	glutamate receptor, ionotropic, AMPA 2	4,32	3,49E-03
DCLK2	doublecortin-like kinase 2	4,39	1,08E-03
PHYHIPL	phytanoyl-CoA 2-hydroxylase interacting protein-like	4,40	9,61E-05
LAMA4	laminin, alpha 4	4,40	2,63E-03
ASCL1	achaete-scute complex homolog 1 (Drosophila)	4,44	3,64E-03
ELMOD1	ELMO/CED-12 domain containing 1	4,47	1,08E-03
SLC4A4	solute carrier family 4, sodium bicarbonate cotransporter, member 4	4,52	2,90E-03
LRRTM2	leucine rich repeat transmembrane neuronal 2	4,54	6,39E-04
GPNMB	glycoprotein (transmembrane) nmb	4,55	2,31E-02
NFIA	nuclear factor I/A	4,59	1,73E-03
MGP	matrix Gla protein	4,71	3,91E-02
TFAP2B	transcription factor AP-2 beta (activating enhancer binding protein 2 beta)	4,78	8,62E-04
CHL1	cell adhesion molecule with homology to L1CAM (close homolog of L1)	4,84	5,33E-03
LOC643763	hypothetical LOC643763	4,85	9,94E-04
HLA-DRA	major histocompatibility complex, class II, DR alpha	4,86	1,20E-02
A2M	alpha-2-macroglobulin	5,05	8,74E-03
SCRG1	stimulator of chondrogenesis 1	5,14	1,50E-03
FABP7	fatty acid binding protein 7, brain	5,18	1,69E-04
LHFPL3	lipoma HMGIC fusion partner-like 3	5,19	2,53E-03
PMP2	peripheral myelin protein 2	5,27	4,03E-03
LOC645323	hypothetical LOC645323	5,36	6,39E-04
GPM6A	glycoprotein M6A	5,46	3,89E-06
HLA-DRA	major histocompatibility complex, class II, DR alpha	5,59	1,38E-02
C1orf61	chromosome 1 open reading frame 61	5,63	6,39E-04
SPARCL1	SPARC-like 1 (hevin)	5,66	9,03E-04
S100B	S100 calcium binding protein B	5,84	1,26E-03

Table S4. List of transcription factors and genomic modifiers involved in pluripotency, ESC biology or oncogenesis downregulated in hiPS-NSCs with respect to hiPSCs (fold change < -1.5; False discovery rate < 0.05).

Biological process	Gene symbol	Fold change	False discovery rate	Reference
Pluripotency and embryonic stem cell biology	LIN28A	-5.73	2.64E-04	[1]
	NANOG	-5.01	1.95E-02	[2]
	LIN28B	-4.71	1.69E-04	[1]
	POU5F1	-4.21	1.05E-02	[3]
	SALL4	-3.20	4.62E-03	[4]
	SOX15	-2.03	9.49E-03	[5]
Oncogenesis	HMGA1	-2.20	1.08E-03	[6]
	RAD51	-1.91	6.36E-03	[7]
	HMGA2	-1.80	2.74E-02	[8]
	ARID3B	-2.70	3.77E-03	[9]
	TFAP2C	-2.59	1.72E-02	[10]
	PDLIM1	-2.34	1.27E-02	[11]
	WDHD1	-1.82	4.55E-02	[12]
	TEAD4	-1.78	6.14E-03	[13]

References

1. Shyh-Chang N, Daley GQ. Lin28: primal regulator of growth and metabolism in stem cells. Cell stem cell. 2013;12:395-406.
2. Pan G, Thomson JA. Nanog and transcriptional networks in embryonic stem cell pluripotency. Cell research. 2007;17:42-49.
3. Shi G, Jin Y. Role of Oct4 in maintaining and regaining stem cell pluripotency. Stem Cell Res Ther. 2010;1:39.
4. Yamaguchi YL, Tanaka SS, Kumagai M et al. Sall4 is essential for mouse primordial germ cell specification by suppressing somatic cell program genes. Stem Cells. 2015;33:289-300.
5. Maruyama M, Ichisaka T, Nakagawa M et al. Differential roles for Sox15 and Sox2 in transcriptional control in mouse embryonic stem cells. The Journal of biological chemistry. 2005;280:24371-24379.
6. Pang B, Fan H, Zhang IY et al. HMGA1 expression in human gliomas and its correlation with tumor proliferation, invasion and angiogenesis. Journal of Neuro-Oncology. 2012;106:543-549.
7. Wiegmans AP, Yap PY, Ward A et al. Differences in Expression of Key DNA Damage Repair Genes after Epigenetic-Induced BRCAness Dictate Synthetic Lethality with PARP1 Inhibition. Mol Cancer Ther. 2015;14:2321-2331.

8. Kaur H, Hutt-Cabezas M, Weingart MF et al. The chromatin-modifying protein HMGA2 promotes atypical teratoid/rhabdoid cell tumorigenicity. Journal of neuropathology and experimental neurology. 2015;74:177-185.
9. Kobayashi K, Jakt LM, Nishikawa SI. Epigenetic regulation of the neuroblastoma genes, Arid3b and Mycn. Oncogene. 2013;32:2640-2648.
10. Gabriely G, Yi M, Narayan RS et al. Human glioma growth is controlled by microRNA-10b. Cancer Research. 2011;71:3563-3572.
11. Ahn BY, Saldanha-Gama RF, Rahn JJ et al. Glioma invasion mediated by the p75 neurotrophin receptor (p75/CD271) requires regulated interaction with PDLIM1. Oncogene. 2015.
12. Sato N, Koinuma J, Fujita M et al. Activation of WD repeat and high-mobility group box DNA binding protein 1 in pulmonary and esophageal carcinogenesis. Clin Cancer Res. 2010;16:226-239.
13. Lim B, Park JL, Kim HJ et al. Integrative genomics analysis reveals the multilevel dysregulation and oncogenic characteristics of TEAD4 in gastric cancer. Carcinogenesis. 2014;35:1020-1027.

Table S5. Summary of hiPSC clones obtained after reprogramming of human fibroblasts from patients affected by Metachromatic Leukodystrophy (MLD). Patient-derived juvenile (MLD1) or fetal (MLD2) fibroblasts were transduced at MOI=1 or MOI=3 with LV.OSK. Reprogramming efficiency was calculated as the percentage of primary hiPSC colonies on the number of plated fibroblasts. The copies of integrated LV genome/host genome (vector copy number, VCN) is expressed as the mean ± SEM, n=3 experiments in triplicates. hiPSC clones highlighted in grey were discarded due to the presence of abnormal karyotype.

Patient's fibroblasts	LV.OSK MOI	Reprogramming efficiency	hiPSC clones	LV.OSK VCN
MLD1	1	0.012%	MLD1.1	3.1 ± 0.1
			MLD1.2	3.1 ± 0.4
			MLD1.3	3.4 ± 0.6
MLD2	3	0.247%	MLD2.1	1.7 ± 0.3
			MLD2.2	0.7 ± 0.1
			MLD2.3	1.5 ± 0.2
			MLD2.4	0.8 ± 0.1

Table S6. Summary of stable ARSA-overexpressing MLD hiPSC lines. Two LV constructs were used to transfer a functional human *ARSA* gene in MLD 1.1, MLD 1.2 and MLD 2.1 hiPSC clones: i) a lab grade/scale bidirectional LV coding for the *hARSA* gene (tagged with HA peptide) and the GFP gene (bdLV.hARSA.GFP); ii) a monodirectional LV carrying a codon optimized *hARSA* coding sequence [30](LV.hARSA). The copies of integrated LV genome/host genome (vector copy number, VCN) are expressed as the mean±SEM, n=3 experiments in triplicates. The percentages of GFP^+ cells in the bdLV.hARSA.GFP-transduced bulk population (pre-sorting) and after sorting for GFP^+ expression are reported. ARSA enzymatic activity [69] was measured in hiPSCs transduced with the two LV constructs as well as in untransduced MLD (MLD1 and MLD2) and ND hiPSCs (ND1 and ND2) and is expressed as mean±SEM, n=2-8 samples/group. N.A., not applicable.

Donors	hiPSC clones	Vector	GFP^+ cells (%)		VCN	ARSA activity (nmol/h*mg)
			pre-sorting	post-sorting		
MLD1	MLD1.1	bdLV.hARSA.GFP	15.4	71.7	1.3 ± 0.3	2,407.9 ± 774.7
		LV.hARSA	N.A.	N.A.	0.4 ± 0.1	4,915.1 ± 1,229.0
	MLD1.2	bdLV.hARSA.GFP	8.9	76.6	16.5 ± 2.0	2,340.3 ± 1,836.5
MLD2	MLD2.1	LV.hARSA	N.A.	N.A.	3.0 ± 0.2	10,918.4 ± 2,192.5
MLD		N.A.	N.A.		N.A.	55.6 ± 43.3
ND						643.0 ± 328.1

Table S7. Experimental groups. The table reports the number and the age at treatment (PND, postnatal days) of immunodeficient MLD mice (Rag$^{-/-}$;γ-chain$^{-/-}$;As$^{-/-}$) and WT littermates (Rag$^{-/-}$;γ-chain$^{-/-}$;As$^{+/+}$) in the different experimental groups, and their use for different assessments. UT, untreated; Tx, transplanted. Histology/immunofluorescence (IF) includes assessment of: cell engraftment, distribution and proliferation (hNuclei, hMito, cell lineage markers, Ki67); tissue sulfatides (Alcian Blue staining).

**Experimental groups**				**Nr. of mice**		
**Mice**	**Treatment**	**Transplanted cells**	**Age at transplant**	**Histology/ IF**	**ARSA activity**	**Total**
Rag$^{-/-}$;γ-chain$^{-/-}$;As$^{+/+}$	UT	na	na	8	4	**12**
Rag$^{-/-}$;γ-chain$^{-/-}$;As$^{-/-}$		na	na	8	3	**11**
Rag$^{-/-}$;γ-chain$^{-/-}$;As$^{-/-}$	Tx	hfNSC	PND60	4	0	**4**
		ND hiPS-NSC	PND60	6	6	**12**
		ARSA-MLD hiPS-NSC	PND60	3	6	**9**
		ND hiPS-NSC	PND1	3	3	**6**
		ARSA-MLD hiPS-NSC	PND1	3	3	**6**

Table S8. List of primary and secondary antibodies used in immunofluorescence analyses.

Primary antibodies		
**Antigen**	**Host species (company, catalog number)**	**Dilution**
Octamer-binding transcription factor 4 (OCT4)	Mouse monoclonal (Santa Cruz, SC5279)	1:200
Homeobox protein NANOG (NANOG)	Rabbit polyclonal (Abcam, AB21624)	1:200
TRA 1-60	Mouse monoclonal (Millipore, MAB4360)	1:100
Chemokin C-X-C motif receptor 4 (CXCR4)	Rat monoclonal (BD bioscience, 551966)	1:100
α-Smooth Muscle Actin (α-SMA)	Mouse monoclonal (Sigma, C6198)	1:400
Hemagglutinin (HA)	Rat monoclonal (Roche, 11867423001)	1:100
Green fluorescent protein (GFP)	Chicken polyclonal (Abcam, ab-13970)	1:500
Arylsulfatase A (ARSA)	Goat polyclonal (R&D system, AF2485)	1:50
β-tubulin III	Mouse monoclonal (Babco, MMS-435P)<br>Rabbit polyclonal (Babco, PRB-435P)	1:1,000<br>1:500
Glial fibrillary acidic protein (GFAP)	Mouse monoclonal (Millipore, MAB3402)<br>Rabbit polyclonal (DAKO, ZO334)	1:1,000<br>1:1,000
Nestin	Mouse monoclonal (Millipore, MAB353)	1:200
Polysialylated-neural cell adhesion molecule (PSA-NCAM)	Mouse monoclonal (Vector Lab, AbC0019)	1:2,000
Antigen Ki67 (Ki67)	Rabbit polyclonal (Novocastra, NCL-Ki67-p)	1:1,000
Neuronal nuclei (NeuN)	Mouse monoclonal (Chemicon, MAB377)	1:300
TH	Rabbit polyclonal (Imun. sciences AB10312)	1:200
GABA	Rabbit polyclonal (Sigma Aldrich A2052)	1:200
Chat	Rabbit polyclonal (Chemicon AB143)	1:100
Somatostatin	Rabbit polyclonal (Chemicon MAB354)	1:200
Calbindin	Rabbit polyclonal (Swant CB-38A)	1:500
A2B5	Mouse monoclonal (Chemicon MAB312)	1:1,000
NG2	Rabbit polyclonal (Chemicon MAB5320)	1:300
OLIG2	Rabbit polyclonal (Chemicon AB9610)	1:500
APC	Mouse monoclonal (Calbiochem OP80)	1:500
CNPase	Mouse monoclonal (Chemicon MAB326R)	1:500

hMITO	Mouse monoclonal (Chemicon MAB1273)	1:300
hNUCLEI	Mouse monoclonal (Millipore, MAB1281)	1:200
MBP	Rat monoclonal (Chemicon MAB386)	1:300
GST[illegible]	Rabbit polyclonal (MBL 312)	1:500
ZO1	Rabbit polyclonal (Invitrogen 61-7300)	1:200
P75-NGFR	APC-conjugated (BD Pharmigen 560326)	1:500
OTX2	Mouse monoclonal (R&D Systems MAB1979)	1:200
**Secondary antibodies**		
**Conjugate**	**Source and product number**	**Dilution**
Alexa 488	Goat anti-mouse IgG (Mol.Probes, A11001)<br>Goat anti-rabbit IgG (Mol.Probes, A11008)<br>Goat anti-chicken IgG (Mol.Probes, A11039)	1:1,000
Alexa 546	Goat anti-mouse IgG (Mol.Probes, A11003)<br>Goat anti-rabbit IgG (Mol.Probes, A11010)<br>Donkey anti-goat IgG (Mol.Probes, A11056)	1:2,000
Cy3	Goat anti-rabbit IgG (Jackson Lab, 111-165-144)<br>Goat anti-rat IgG (Jackson Lab, 112-165-006)	1:2,000

Table S9. List of custom primers annealing the bisulfite converted endogenous promoters of NANOG and OCT4 genes.

Promoter	Primer Sequence
NANOG	For 5'- TGG TTA GGT TGG TTT TAA ATT TTT G -3'
	Rev 5'- ATT AAC TCT TAA ATA TTC CCA CCC AA -3'
OCT4	For 5'-GAG GTT GGA GTA GAA GGA TTG TTT TGG TTT- 3'
	Rev 5'- AAT CCA CCA CCT CCA CTA CCC AAT CCC CCC -3'

Table S10. List of primers used in RT-PCR analyses. Primers designed to recognize exclusively the endogenous KLF4 and SOX2 are tested for their specificity by using LV.OSK plasmid as template observing no amplification of PCR products.

Gene	Primer Sequence
Endogenous KLF4	For 5'-ACCCACACAGGTGAGAAACCTT-3'
	Rev 5'-GTTGGGAACTTGACCATGATTG-3'
Endogenous SOX2	For 5'-TTACCTCTTCCTCCCACTCCAG-3'
	Rev 5'-GGGTTTTCTCCATGCTGTTTCT-3'
NANOG	For 5'- GAT CGG GCC CGC CAC CAT GAG TGT GGA TCC AGC TTG-3'
	Rev 5'- GAT CGA GCT CCA TCT TCA CAC GTC TTC AGG TTG-3'
GAPDH	For 5'- CAG CCT CAA GAT CAT CAG CA -3'
	Rev 5'- TGC TGT AGC CAA ATT CGT TG -3'
SOX17	For 5'-TTCATGGTGTGGGCTAAGGAC-3'
	Rev 5'-AGTTGGGGTGGTCCTGCATG-3'
CD146	For 5'-AAGGCAACCTCAGCCATGTC-3'
	Rev 5'-AGAAGATGCGCTCGTCTTGG-3'
CXCR4	For 5'- CAG CAG GTA GCA AAG TGA CG -3'
	Rev 5'- TTG TCC GTC ATG CTT CTC AG -3'
PDGFRβ	For 5'-AGACACGGGAGAATACTTTTGC-3'
	Rev 5'-AGTTCCTCGGCATCATTAGGG-3'
α-SMA	For 5'-GTGTTGCCCCTGAAGAGCAT-3'
	Rev 5'-GCTGGGACATTGAAAGTCTCA-3'
PAX6	For 5'- AAC AGA CAC AGC CTT CAC AAA CA-3'
	Rev 5'- CGG GAA CTT GAA CTG GAA CTG AC-3'
MAP2	For 5'- AAG AAG GTC GCC ATC ATA CG -3'
	Rev 5'- GGG CTT TAG CAT GCT CTC TG -3
HA	For 5'- CAA GGA CCC TGG TGA GAA CT -3'
	Rev 5'- GAA CCC GGG GTA CCT CAA -3'
GFP	For 5'- AAG TCG TGC TGC TTC ATG TG -3'
	Rev 5'- ACG TAA ACG GCC ACA AGT TC -3'
NESTIN	For 5'- TCC AGG AAC GGA AAA TCA AG -3'
	Rev 5'- TAG AGA CCT CCG TCG CTG TT -3'
PDGFRα	For 5'- ACA TTG ACC CTG TCC CTG AG -3'
	Rev 5'- TTT CTG AAC GGG ATC CAG AG -3'
PLP-DM20	For 5'- GGC CAC TGG ATT GTG TTT CT -3'
	Rev 5'- AGG TGG TCC AGG TGT TGA AG -3'
GFAP	For 5'- ACA TCG AGA TCG CCA CCT AC -3'
	Rev 5'- CGG AGC AAC TAT CCT GCT TC -3'
β-tubulin III	For 5'- CTT CGG CCA GAT CTT CAG AC-3'
	Rev 5'- ACA GGG CCT CGT TAT CAA TG -3'

Table S11. List of primers used in Sybr Green qRT-PCR analyses.

Gene	Primer Sequence
GAD65	For 5’- ATC CTC ACG ACT CAG CTC CC -3’
	Rev 5’- GAG CTT TAA AAG AGA CCG GGA CT -3’
TH	For 5’- GAG TAC ACC GCC GAG GAG ATT G -3’
	Rev 5’- GCG GAT ATA CTG GGT GCA CTG G -3’
vGLUT	For 5’- GGT CGT GCT CTC GGG AAG -3’
	Rev 5’- ATG CCA AAG CTG ATG CAG AA -3’
SLC18A3	For 5’- TGC TCT ATG CTC CCG TCT TG -3’
	Rev 5’- CAC CGC ATC GTA CAG ACC TT -3’
TpH2	For 5’- AAA TAG GTC TGG CGT CTC TGG -3’
	Rev 5’- AGG GCG TGC TTT AAT TCT CC -3’
PET1	For 5’- CAG AAA GGC AGC GGA CAG -3’
	Rev 5’- TTC ATG TTG GGC TTG CTC TT -3’
NANOG	For 5’- ATG CCT CAC ACG GAG ACT GT -3’
	Rev 5’- AAG TGG GTT GTT TGC CTT TG -3’
LIN28	For 5’- GAA GCG CAG ATC AAA AGG AG -3’
	Rev 5’- GCT GAT GCT CTG GCA GAA GT -3’
OCT4	For 5’- TCG AGA ACC GAG TGA GAG G -3’
	Rev 5’- GAA CCA CAC TCG GAC CAC A -3’
PAX6	For 5'- AGT GAA TCA GCT CGG TGG TGT CTT -3'
	Rev 5'- TGC AGA ATT CGG GAA ATG TCG C -3'
DCX	For 5’- TGG ATG AAA ATG AAT GCC GAG T -3’
	Rev 5’- AGC TGG AGA CTT GCT TCG G -3’
ASCL1	For 5’- TCC CCC AAC TAC TCC AAC GA -3’
	Rev 5’- GCG ATC ACC CTG CTT CCA AA -3’
GAPDH	For 5'- TCA AGA AGG TGG TGA AGC AGG -3'
	Rev 5'- ACC AGG AAA TGA GCT TGA CAA A -3'
